# Synthesis, Characterization, Antimicrobial and Anticancer Evaluation of Novel Heterocyclic Diazene Compounds Derived from 8-Quinolinol

**DOI:** 10.3390/ph19010004

**Published:** 2025-12-19

**Authors:** Ion Burcă, Alexandra-Mihaela Diaconescu, Valentin Badea, Francisc Péter

**Affiliations:** 1 Department of Applied Chemistry and Organic and Natural Compounds Engineering, Politehnica University Timisoara, Vasile Pârvan 6 Blvd., 300223 Timisoara, Romania; ion.burca2@student.upt.ro (I.B.); alexandra.diaconescu@student.upt.ro (A.-M.D.); francisc.peter@upt.ro (F.P.); 2 Renewable Energy Research Institute-ICER, Politehnica University Timișoara, Gavril Musicescu Str. 138, 300501 Timișoara, Romania

**Keywords:** azene compound, 8-quinolinol, amino pyrazole, Hep-G2 cell line, antimicrobial, anticancer

## Abstract

**Background**: 8-Quinolinol and its derivatives are drawing significant attention across various disciplines due to their remarkable versatility. These compounds are well-known for their exceptional chelating ability, forming stable metal complexes via their nitrogen and oxygen electron donor atoms. This main characteristic determines their broad utility. Biological activity can also be explained by the chelating capacity, which allows 8-quinolinol to bind to essential metal ions such as Fe, Zn, Cu, and others. This chelation disrupts metal-dependent biological processes in target cells or organisms, leading to a range of effects, including antimicrobial, anticancer, antifungal, and neuroprotective activities. On the other hand, the biological activity of pyrazole derivatives is attributed to their heterocyclic structure, which allows for interactions with biological targets that can lead to enzyme inhibition, receptor antagonism, radical scavenging, and other effects. **Objective:** This work aimed to synthesize and characterize novel diazene compounds derived from 8-quinolinol or 2-methyl-8-quinolinol and pyrazole amines, and to evaluate their antimicrobial and anticancer activities. **Methods:** The compounds have been synthesized by coupling diazonium salts obtained from the diazotization of heterocyclic amines with 8-quinolinol and its derivative, 2-methyl-8-quinolinol. The careful selection of reaction conditions enabled the synthesis of high-purity products. The compounds were characterized by 1D and 2D NMR, FT-IR spectroscopy, UV-Vis spectroscopy, and LC-HRMS analysis. The biological activity of the newly synthesized compounds was evaluated following the protocols of EU-OPENSCREEN, a European Research Infrastructure Consortium (ERIC) initiative dedicated to supporting early drug discovery. **Results:** By combining diazonium salts obtained from 3-methyl-1*H*-pyrazol-5-amine and ethyl 5-amino-3-methyl-1*H*-pyrazole-4-carboxylate with the aforementioned coupling agents, four novel 8-quinolinol derivatives were synthesized. The further hydrolysis of the ethoxy carbonyl functional group allowed its conversion to a carboxylic functional group, thus expanding the series of new compounds to six members. Several compounds from the series have proven to be biologically active against several human pathogenic microorganisms and the Hep-G2 cancer cell line. **Conclusions:** The combination of two well-known biologically active scaffolds through a classic diazo coupling reaction allowed the synthesis of novel biologically active compounds, which showed promising results as possible antifungal and anticancer agents. These results represent a foundation for future studies, which will include a broader biological screening and in vivo studies.

## 1. Introduction

8-Quinolinol (8-hydroxyquinoline, 8-Hq) and its derivatives have received significant attention from researchers due to their versatile, wide-ranging applications in environmental, medicinal and analytical chemistry. Among the aromatic bicyclic heterocyclic compounds, 8-Hq received special interest due to its versatility to be transformed into derivatives with wide uses in industrial and medical chemistry, including marketed preparations [1,2]. The chelating ability of 8-Hq originates from the nitrogen and oxygen electron donor atoms, creating a bidentate binding site and allowing the formation of stable metal complexes with diverse applications, from chemosensors to biologically active compounds [3,4,5,6,7].

There are several ways in which 8-Hq or its derivatives can influence biological targets. One of them makes direct use of their unique chelating ability, which allows binding to metal ions. Once chelated, the metal ion can be transported closer to the target, thus increasing its therapeutic efficiency. Some 8-Hq derivatives exhibit this property, and it has been proven that they can increase the intracellular copper levels. Through copper-induced toxicity, they can be used in tuberculosis treatment [8]. Another way of hitting biological targets relies on the ability of some 8-Hq derivative complexes with gallium to increase the reactive oxygen species (ROS) concentration in cells. This property was successfully used in targeting colon cancer cells [9]. The same anticancer action, achieved by increasing the concentration of ROS, was confirmed for copper complexes of 8-Hq derivatives [10]. By chelating metal cofactors such as iron or zinc, 8-Hq disrupts metal homeostasis within bacterial and fungal cells, impairing their metabolic functions and growth. Some studies show that certain metal 8-Hq complexes can be more potent than the ligand itself [11].

Pyrazole is a significant five-membered heterocyclic compound with two adjacent nitrogen atoms. Its derivatives are known for a wide range of biological activities, including anticancer, anti-inflammatory, and antimicrobial effects. The mechanism of action often involves targeting key enzymes and signaling pathways within the cell. The anticancer activity of pyrazole derivatives is manifested by targeting specific proteins such as tubulin, epidermal growth factor receptor (EGFR), cyclin-dependent kinases (CDK), and Bruton’s tyrosine kinase (BTK), thereby impeding tumor growth and proliferation [12,13]. Pyrazole derivatives can also prevent biofilm formation, a major factor in antibiotic resistance, which also recommends them as potent antimicrobial agents [14,15]. Anti-inflammatory action of pyrazole containing compounds is manifested in different ways, several of which are inhibition of the Nuclear Factor-kappa B (NF-κB) pathway [16], which is a critical transcription factor that controls the expression of inflammatory genes, lipoxygenase (an enzyme involved in the synthesis of pro-inflammatory mediators) inhibition or lipid peroxidation (a process that contributes to oxidative stress and inflammation) inhibition [17].

According to several SAR studies performed on 8-Hq derivatives, the substituents on the quinoline ring play a crucial role in compounds’ biological activities. For example, in a series of 8-hydroxyquinoline-derived Mannich bases active against some forms of multi-drug-resistant cancer cells (MDR), the presence of halogen atoms (like fluorine or chlorine) in the R5 position on the 8-Hq ring increased both toxicity and selectivity, while alkoxy methyl groups in the R5 position increased selectivity but lowered the compound’s overall toxicity [18]. In another study, SAR studies revealed that the most effective antimicrobial compounds feature a combination of quinoline and other heterocycles or fused rings that likely allow them to bind to and inhibit key microbial targets more effectively [19].

**Figure 1:**
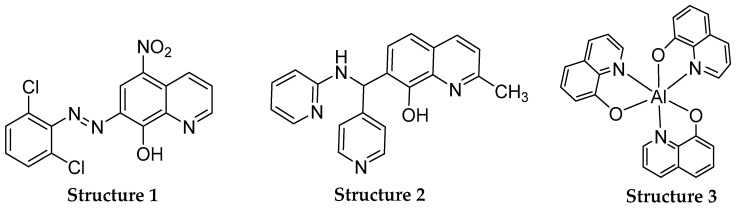
Structures of some biologically active 8-Hq derivatives with demonstrated biological activity.

Examples of 8-Hq derived structures that exhibit biological activity, presented in Figure 1, are 7-(2,6-dichlorophenyl)diazenyl)-5-nitroquinolin-8-ol (Structure 1), which, in combination with metal ions such as Zn, Cu, and Ni, forms complexes with good activity in adenosine deaminase enzyme (ADA) [20]; 7-((pyridin-4-yl)(pyridin-2-ylamino)methyl)-2-methylquinolin-8-ol (Structure 2), which demonstrated promising results in suppressing GLI (Glioma-associated oncogene protein family) mediated transcription [21]; and tris(8-hydroxyquinoline)aluminum (Structure 3), a complex used as a common optic material that also exhibits antibacterial properties, which can be utilized in antimicrobial coatings [22].

Despite the advances in the field, there are still challenges remaining in synthesizing 8-Hq derivatives with enhanced specificity, stability, and multifunctionality. In this paper, we report the synthesis and characterization of new azo 8-Hq derivatives with pyrazole functionalities, as a continuation of our research work in the topic of heterocyclic diazene compounds [23,24]. By using synthetic strategies involving heterocyclic amines and diazotization/diazo coupling reactions, we seek to expand the scope of 8-Hq diazene compounds for possible applications in medicinal chemistry by synthesizing derivatives that contain two powerful biologically active scaffolds. Although the versatility of the quinoline’s scaffold enabled numerous chemical modifications, including the attachment of various N-heterocyclic structures, pyrazoles have not yet been explored in this direction.

## 2. Results

### 2.1. Chemical Synthesis and Characterization of 8-Quinolinol Derivatives with Pyrazole Moieties

**Scheme 1:**
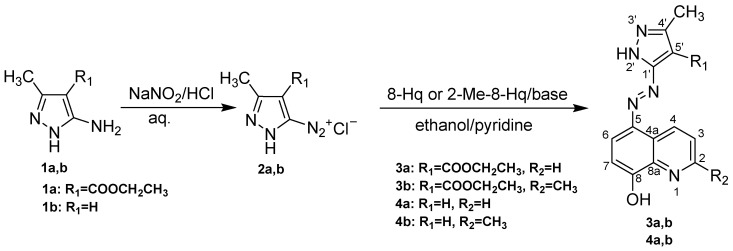
Reaction scheme of diazotization of the heterocyclic amines (**1a**) and (**1b**) and the coupling reaction of the resulting diazonium salts.

**Scheme 2:**
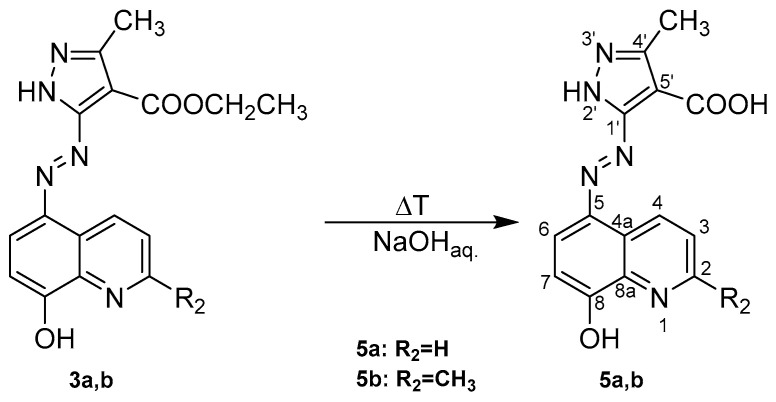
Base-catalyzed hydrolysis reaction of the compounds (**3a**) and (**3b**).

New 8-Hq derivatives with pyrazole moieties attached through a diazene bridge were synthesized using a validated reaction pathway, their structure and purity being confirmed by analytical methods. Two diazonium salts (**2a**) and (**2b**), obtained by the diazotization of the amino groups of the heterocyclic amines ethyl 5-amino-3-methyl-1*H*-pyrazole-4-carboxylate (as hydrochloride) (**1a**) and 3-methyl-1*H*-pyrazol-5-amine (**1b**), were used in diazo coupling reactions with 8-Hq or 2-methyl-8-quinolinol (2-Me-8-Hq). By combining the two diazonium salts and the two coupling agents, the synthesis of four diazene compounds was achieved, as follows: (**3a**) ethyl 5-((8-hydroxyquinolin-5-yl)diazenyl)-3-methyl-1*H*-pyrazole-4-carboxylate, (**3b**) ethyl 5-((8-hydroxy-2-methylquinolin-5-yl)diazenyl)-3-methyl-1*H*-pyrazole-4-carboxylate, (**4a**) 5-((3-methyl-1*H*-pyrazol-5-yl)diazenyl)quinolin-8-ol and (**4b**) 2-methyl-5-((3-methyl-1*H*-pyrazol-5-yl)diazenyl)quinolin-8-ol. The overall reaction path is shown in Scheme 1:

The diazene compounds (**3a**) and (**3b**) bearing ester functional groups were further irreversibly hydrolyzed in alkaline medium under heating, thus resulting two new azo compounds containing a carboxylic acid functional group: (**5a**) 5-((8-hydroxyquinolin-5-yl)diazenyl)-3-methyl-1*H*-pyrazole-4-carboxylic acid and (**5b**) 5-((8-hydroxy-2-methylquinolin-5-yl)diazenyl)-3-methyl-1*H*-pyrazole-4-carboxylic acid, as shown in Scheme 2:

A comprehensive characterization of the synthesized new heterocyclic compounds has been performed by spectroscopic and chromatographic techniques, also to prove their structure and purity. The key structural analysis was accomplished by Two-Dimensional Nuclear Magnetic Resonance (2D NMR), and Table 1, Table 2, Table 3, Table 4, Table 5 and Table 6 summarize these results for each synthesized compound. The main direct and long-range correlations between protons and the ^13^C and ^15^N nuclei were obtained from ^1^H-^13^C Heteronuclear Single Quantum Correlation (HSQC), ^1^H-^13^C Heteronuclear Multiple Bond Correlation (HMBC), ^1^H-^15^N HMBC spectra, while the long-range proton–proton correlations resulted from 2D ^1^H-^1^H Correlation Spectroscopy (COSY). All these correlations enabled an unambiguous assignment of the signals of the protons and the ^13^C nuclei. The long-range indirect correlation spectra between protons and ^15^N nuclei provided, in most cases, the corresponding ^15^N resonances, particularly those of the quinoline 1-N and the pyrazole 3′-N atoms. A more detailed discussion of these results is presented in Section 3.

The FT-IR data (Table S1, Supplementary Materials) reveal the presence of the characteristic bands for the most prominent bonds in the synthesized structures: O-H, N-H, and C-H stretches are observed in the expected absorption ranges. For the compounds which contain a carbonyl functional group, the characteristic stretch is observed at around 1700 cm^−1^, and N-H bending bands are observed at 1400–1500 cm^−1^.

Based on the UV-Vis data (Table S2, Supplementary Materials), the synthesized compounds exhibit absorption in the visible spectrum with a λ_max_ in the range of 385–393 nm. However, their molar absorption coefficients, which are on the order of 10^5^ M^−1^ cm^−1^ (with a maximum value of 30,400 M^−1^ cm^−1^), are not high enough to consider these compounds suitable as diazo dyes.

The Liquid Chromatography-High Resolution Mass Spectrometry (LC-HRMS) analysis (Table S3, Supplementary Materials) confirmed the structures and demonstrated the high purities of the synthesized compounds. The chromatographic analysis showed one single compound peak for all isolated products, and the molecular mass values resulting from the mass spectral analysis were similar to those calculated based on the chemical structures. The MS data, together with NMR and FT-IR results, undoubtedly prove the presumed structures of the obtained products.

### 2.2. Screening of the Biological Activities

All synthesized compounds were investigated for potential biological activity against nine human pathogenic microorganisms and one human hepatic cancer cell line, using the EU-OPENSCREEN protocols (described in Section 4.2.4 and in the Supplementary Materials), designated to allow early-stage drug discovery [25].

Table 7, Table 8, Table 9 and Table 10 present the mean inhibition percentages for each compound at the tested concentrations (50 μM for microorganisms and 10 μM for cancer cell lines) in two test runs, compared with the biological activities for known and widely used anti-bacterial, anti-fungal, and anti-cancer compounds reported in the literature. More detailed biological activity data (including the standard deviations) are presented in Tables S4 and S5, Supplementary Materials.

The biological activity evaluation showed very promising results concerning the anticancer effects, all synthesized compounds (**3a**), (**3b**), (**4a**), (**4b**), (**5a**) and (**5b**) demonstrating significant in vitro antiproliferative activities against the Human Hepatocellular Carcinoma (Hep-G2) Cells, comparable to the activity of the market drug Etoposide. Specifically, the compounds (**4a**) and (**5a**) showed high cytotoxic activity against liver cancer cells, exhibiting excellent IC_50_ values of 2.99 ± 0.3 µM and 2.71 ± 0.3 µM, respectively (Table S4, Supplementary material).

Additionally, the synthesized diazene compounds have been proven to be active with high inhibition percentages against two of the three tested fungi, *C. albicans* and *C. auris*. Against *A. fumigatus*, only compounds (**3a**), (**4a**), and (**5a**) manifested inhibitory activity (defined as an average of the percentage of inhibition ≥ 70), while the compounds that contain a methyl group in position 2 of the 8-quinolinol moiety, (**3b**), (**4b**), and (**5b**), did not.

As concerns the antibacterial activity, it was completely absent against the Gram-negative and low against the Gram-positive microorganisms.

## 3. Discussion

The targeted 8-Hq derivatives were synthesized through diazotization of the appropriate pyrazole amines, followed by coupling with 8-Hq or 2-Me-8-Hq, and hydrolysis in the case of the compounds substituted with ester groups in position 2 of the quinoline moiety. The isolation yields subsequent to all reaction steps were higher than 70%, except for the hydrolysis step producing compound (**5b**), which was performed with 53% yield. Literature studies show that heterocyclic amines can be diazotized in the same way as aromatic amines [34,35,36,37]. The obtained heterocyclic diazo compounds are similarly reactive to diazo compounds synthesized from aromatic amines. The heterocyclic counterparts can also be involved in electrophilic aromatic substitution reactions. An example of such a reaction is the diazo coupling reaction, which usually involves an electrophilic agent and a coupling agent. In the present work, the heterocyclic diazo compounds acted as electrophilic agents, and 8-quinolinol and its derivative, 2-methyl-8-quinolinol, were chosen as the coupling agents. In order to improve the reactivity towards electrophilic substitution reactions, before the diazo coupling reaction, coupling agents were dissolved in an appropriate organic solvent together with a proper quantity of sodium hydroxide. In alkaline medium, the phenolic proton is donated; thus, the 8-quinolinato and 2-methyl-8-quinolinato anions are formed. The electron density for these anions is concentrated in positions 5 and 7, while the pyridine ring of the 8-quinolinol moiety remains relatively unreactive in diazo coupling reactions. In one of our previous studies [23], we proved based on NMR data analysis that the coupling takes place exclusively in position 5 of 8-quinolinol moiety if the azo coupling reaction is performed under the conditions described in the current paper.

The presumed structures and the purity of all synthesized compounds were confirmed by 2D NMR, FT-IR and LC-HRMS analysis. Only for compound (**4b**) recrystallization from methanol was necessary; the others were obtained following the downstream isolation steps in pure form (>99%), appropriate for the biological screening.

The 2D NMR results presented in Table 1, Table 2, Table 3, Table 4, Table 5 and Table 6, based on the spectra included as Supplementary material (Figures S3–S41), allow the identification of the main structural characteristics of the synthesized compounds. The 2D NMR HSQC (^1^H-^13^C) spectra confirm, through the observed cross-peaks, the expected one-bond couplings between the quinolinic protons and their directly attached carbon atoms for compounds **3a**, **4a** and **5a** (2-C-H, 3-C-H, 4-C-H, 6-C-H, 7-C-H), and for compounds **3b**, **4b** and **5b** (3-C-H, 4-C-H, 6-C-H, 7-C-H). Hydrolysis and decarboxylation of compounds **4a** and **4b** are supported by the presence of the pyrazolic 5′-H proton signals at 6.52 ppm and 6.49 ppm, respectively, which show direct coupling to the corresponding pyrazolic 5′-C atoms at 92.5 ppm in the HSQC ^1^H-^13^C spectra.

For all synthesized compounds, the 2D NMR HMBC (^1^H-^13^C) spectra exhibit the characteristic three-bond correlations between the methyl carbons 4′-C-CH_3_ and the pyrazolic carbon 5′-C. The HMBC spectra also reveal two-bond couplings between the methyl protons 2-C-CH_3_ and the quinolinic carbon 2-C.

The HMBC ^1^H-^15^N spectra further highlight two-bond correlations between the quinolinic nitrogen 1-N and proton 2-H in compounds **3a** and **4a**. Additionally, in compounds **3b**, **4b**, and **5b**, three-bond correlations are observed between the quinolinic nitrogen 1-N and the methyl protons 2-C-CH_3_.

The 2D COSY (^1^H-^1^H) spectra confirm the expected spin–spin couplings: between 2-H and 3-H in compounds **3a**, **4a** and **5a**, between 3-H and 4-H in **3b**, **4b** and **5b**, and between 6-H and 7-H across all synthesized compounds.

In the FT-IR spectra (supplementary material, Figures S42–S47), the O-H stretching bands (ν_OH_) for all compounds appear between 3300–3500 cm^−1^. The ester carbonyl stretching vibrations (ν_C = O_) in compounds **3a** and **3b** are observed at 1697 cm^−1^ and 1694 cm^−1^, respectively. For compounds **3a**, **4a** and **5a**, additional N-H stretching bands (ν_NH_) associated with the pyrazolic NH groups are detected between 3210–3272 cm^−1^. All synthesized compounds also display the characteristic in-plane N-H deformation bands (δ_NH_) of the pyrazolic NH groups in the 1504–1567 cm^−1^ region.

### Biological Activity Evaluation

The synthesized diazene compounds, derived from 8-quinolinol or 2-methyl-8-quinolinol and pyrazole amines, were screened against various human pathogens and HepG2 cells through the EU-OPENSCREEN ERIC initiative, using the high-throughput screening (HTS) methodology commonly employed in early-stage drug discovery. HTS is a rapid, automated method used in drug discovery and molecular biology to screen large libraries of chemical or biological compounds against a specific target. It is a fast and efficient way to identify “hits” or compounds that have a desired effect on a biological process. Compounds that show a significant effect—based on predefined criteria (e.g., a certain percentage of growth inhibition)—are identified as hits [38,39,40,41].

The most important outcome of this research is provided by the excellent results of the preliminary in vitro evaluation of the synthesized compounds as antiproliferative agents against hepatocellular carcinoma (HepG2) cells. Cytotoxic properties were reported for several 8-Hq derivatives. Sokal et al. synthesized 8-Hq derivatives with 1,4-naphthoquinone and observed higher cytotoxicity of these hybrids compared to cisplatin against lung and breast cancer cell lines [42]. Zieba et al. obtained several 8-hydroxyquinoline-5-sulfonamides and 8-methoxyquinoline-5-sulfonamides, concluding that the anticancer activities of these compounds are related to the presence of a free phenolic group in position 8 of the quinoline pharmacophore [43]. Our results show that compounds with 8-Hq and pyrazine moieties display strong antineoplastic activity against hepatocellular carcinoma cells, with lower IC_50_ values compared to the commercial drug etoposide. Obviously, these are preliminary results demonstrating the potential of these 8-Hq derivatives. Further studies are needed to investigate the mechanisms as possible multi-target agents and better explain the biological activity, also against other cancer types, as well as to examine their potential use as co-drugs with existing anticancer agents.

Several structure-activity relationship (SAR) conclusions can be drawn from the provided biological activity data, particularly concerning the antifungal activity. Compounds (**4a**) and (**4b**), which lack substituents at the 5′-C position of the pyrazole ring, exhibited the highest inhibition against *C. albicans*. Conversely, compound (**3b**), with a bulky ethoxy carbonyl substituent at the 5′-C position, showed the lowest inhibition against *C. albicans*, suggesting that this substituent reduces antifungal activity. Biological activity against *A. fumigatus* correlates with the presence of a methyl substituent at the 2-C position of the quinoline ring; thus, only compounds (**3a**), (**4a**), and (**5a**) exhibit activity against this fungus. The substituent at the 5′-C position of the pyrazole ring does not affect the activity against *A. fumigatus*. Inhibitory activities against *C. auris* are similar, regardless of substituents at the 5′-C or 2-C positions.

The anticancer activity also correlates with the presence of substituents at 5′-C or 2-C positions. Compound (**5a**), with a carboxylic substituent at the 5′-C position and no substituent at the 2-C position, demonstrated the highest cancer cell growth inhibition, whereas compound (**3b**), bearing an ethoxy carbonyl group at the 5′-C position and a methyl group at the 2-C position of the quinoline ring, exhibited the weakest anticancer activity.

The absence of antibacterial activity is probably due to the presence of the pyrazole scaffold, regardless of the substituents. Rbaa et al. synthesized 8-Hq derivatives with different substituents in the 5-position and reported antibacterial activities for Gram-positive and Gram-negative microorganisms higher than Penicillin G, particularly for 5-((2-hydroxyethyl)thiomethyl)quinolin-8-ol, containing a substituted thiomethyl group [44].

A dual, anticancer and antimicrobial activity was reported for several classes of compounds, including 8-hydroxyquinoline derivatives [11]. A relationship between the anticancer and antimicrobial activity is mainly based on targeting cellular processes that are characteristic and essential for both rapidly dividing cancer cells and proliferating microbial cells. Such dual activity could be o solution for the problem of bacterial infections in cancer patients and will represent an important goal of future studies.

## 4. Materials and Methods

### 4.1. Materials

All reagents used in the chemical syntheses were purchased from Merck (Darmstadt, Germany) and used as received, except for the 5-amino-3-methyl-1*H*-pyrazole-4-carboxylate hydrochloride, which was synthesized in our laboratory following modified literature procedures [45,46]. The microbial strains and Hep-G2 cancer cell line used for the assessment of the biological activities were acquired by the institutions involved in the EU-OPENSCREEN ERIC initiative.

### 4.2. Methods

#### 4.2.1. Spectroscopy and Chromatographic Analysis

NMR spectra were recorded on a Bruker Avance III 500 MHz spectrometer (Bruker Daltonics, Billerica, MA, USA). Chemical shifts (δ) were measured in ppm, and the coupling constants (J) were measured in Hz. TMS was used as an internal standard. The samples were dissolved in Py-*d5* or DMSO-*d6*.

Melting points were assessed using a Böetius PHMK apparatus (Veb Analytik, Dresden, Germany) and were not corrected.

UV–Vis spectra were recorded on a Jasco V-530 spectrometer (Jasco Corporation, Tokyo, Japan), and the samples were dissolved in 96% ethanol.

IR spectra were recorded on a Jasco FT/IR-410 spectrophotometer (Jasco Corporation, Tokyo, Japan) in KBr pellets.

LC-HRMS analysis was performed on an AGILENT 1290 LCTOF (Agilent, Santa Clara, CA, USA) system with the following components: Multisampler G7167B, Flex pump G7104A, High speed pump G7120A, DAD WR G7115A, Isopump G7110B, TOF: G6230B.

#### 4.2.2. General Procedure for the Synthesis of Diazonium Salts (**2a**) and (**2b**)

The proper amount of ethyl 5-amino-3-methyl-1*H*-pyrazole-4-carboxylate hydrochloride (0.545 g; 2.625 mmol) or 3-methyl-1*H*-pyrazol-5-amine (0.263 g; 2.625 mmol) was dissolved in hydrochloric acid solution (1:5 or 1:3 *v*/*v*) under stirring. The resulting orange-yellow solutions were cooled down in ice water baths.

Sodium nitrite solution (0.192 g; 2.756 mmol) in 1 mL of distilled water was prepared by dissolving the salt under stirring. The obtained solution was also cooled in an ice water bath.

Once the temperatures of the solutions reached below 5 °C, sodium nitrite solutions were added dropwise to the acidic heterocyclic amine solutions, under stirring. Stirring was maintained for 15–30 min, while the temperature of the reaction mixture was kept under 5 °C. The diazonium salts were not isolated and were used in the further steps as obtained.

#### 4.2.3. General Procedure of the Diazo Coupling and Hydrolysis Reactions for the Synthesis of Compounds (**3a**), (**3b**), (**4a**), and (**4b**)

8-Quinolinol (0.367 g; 2.5 mmol) or 2-methyl-8-quinolinol (0.410 g; 2.5 mmol) and sodium hydroxide (0.315 g; 7.875 mmol) were dissolved in a proper amount of 96% ethanol/pyridine solution. The proportions varied depending on the synthesis and ranged from 1:1 to 1:3 (*v*/*v*). Both solutions were cooled down to a temperature below 5 °C before advancing to the next step.

Diazonium salt solutions were added dropwise to the coupling agents’ solutions, maintaining vigorous stirring and cooling. The pH was adjusted to 7–8, using 30% NaOH solution. The cooling and stirring were maintained for 5–7 h.

After the reaction was completed, the pH of reaction media was brought to a value in the 5.5–6.5 range using 99.8% acetic acid. This pH range was found to be optimal for precipitation of the azo compounds. In order to ensure complete precipitation, the obtained water suspensions were poured over distilled water (200–400 mL).

The precipitate was then separated via vacuum filtration. After several washes with water on filter paper, the products were left to air dry.

The esters (**5a**) and (**5b**) were hydrolyzed in 10% aqueous sodium hydroxide at reflux, according to a procedure previously reported [23].

**Ethyl 5-((8-hydroxyquinolin-5-yl)diazenyl)-3-methyl-1*H*-pyrazole-4-carboxylate (3a)**

Orange solid, yield: 75.6%, m.p.: 218–219 °C; ^1^H NMR: δ_H_ (Py-*d5*, 500 MHz, ppm): 9.45 (d, 1H, *J* = 8.41 Hz, 4-H), 8.97 (dd, 1H, *J* = 4.00 Hz, 1H, 2-H), 8.49 (d, 1H, *J* = 8.56 Hz, 6-H), 7.52 (d, 1H, *J* = 8.56 Hz, 7-H), 7.47 (dd, 1H, *J*_1_ = 8.56 Hz, *J*_2_ = 4.07 Hz, 3-H), 4.47 (q, 2H, *J* = 7.13 Hz, -O-CH_2_-CH_3_), 2.73 (s, 3H, -CH_3_), 1.32 (t, 3H, *J =* 7.13 Hz, -O-CH*_2_*-CH*_3_*); ^13^C NMR: δ_C_ (Py-*d5*, 125 MHz, ppm): 164.8 (-C=O), 160.6 (8-C), 159.4 (4′-C), 150.7 (1′-C), 149.6 (2-C), 140.8 (8a-C), 139.7 (4a-C), 133.3 (4-C), 128.6 (5-C), 123.6 (3-C), 118.9 (6-C), 113.0 (7-C), 108.1 (5′-C), 60.8 (-O-CH_2_-CH_3_), 15.1 (-O-CH_2_-CH_3_), 13.6 (-CH_3_); ^15^N NMR: δ_N_ (Py-*d5*, 50.66 MHz, ppm): 298.8 (1-N); FT-IR (cm^−1^): 3501 (ν_OH_), 3272 (ν_NH_), 2982 (ν_Car-H_), 1697(ν_C = O_), 1553 (ν_Car. = Car._), 1505 (δ_NH_), 1472, 1455 (δ_CH_); UV-Vis (96% ethanol): λ_max_ = 393 nm, ε(λ_max)_ = 11,502 M^−1^ cm^−1^; LC-HRMS (*m*/*z*): [M + H]^+^ for C_16_H_15_N_5_O_3_ calcd. 326.1248, found 326.1245, [M + Na]^+^ for C_16_H_15_N_5_O_3_ calcd. 348.1067, found 348.1065.

All spectra are shown in the Supplementary Materials (Figures S1–S7, S42, S48 and S54).

**Ethyl 5-((8-hydroxy-2-methylquinolin-5-yl)diazenyl)-3-methyl-1*H*-pyrazole-4-carboxylate (3b)**

Orange-brown solid, yield: 83.4%, m.p.: 184–186 °C; ^1^H NMR: δ_H_ (Py-*d5*, 500 MHz, ppm): 9.36 (d, 1H, *J* = 8.64 Hz, 4-H); 8.42 (d, 1H, *J* = 8.50 Hz, 6-H); 7.50 (d, 1H, *J* = 8.50 Hz, 7-H); 7.33 (d, 1H, *J* = 8.64 Hz, 3-H); 4.47 (q, 2H, *J* = 7.09 Hz, -CH_2_-CH_3_); 2.74 (s, 3H, 4′-C-CH_3_); 2.56 (s, 3H, 2-C-CH_3_); 1.33 (t, 3H, *J* = 7.09 Hz,-CH_2_-CH_3_); ^13^C NMR: δ_C_ (Py-*d5*, 125 MHz, ppm): 164.3 (-C=O); 159.3 (8-C); 157.9 (2-C); 148.8 (4′-C); 140.5 (1′-C); 138.6 (8a-C); 133.1 (4-C); 126.1 (4a-C); 124.3 (3-C); 123.8 (5-C); 117.8 (6-C); 112.3 (7-C); 107.6 (5′-C); 60.6 (-CH_2_-CH_3_); 24.6 (2-C-CH_3_); 14.6 (CH_2_-CH_3_); 13.1 (4′-C-CH_3_); ^15^N NMR: δ_N_ (Py-*d5*, 50.66 MHz, ppm): 291.6 (1-N); FT-IR (cm^−1^): 3352 (ν_OH_), 3071 (ν_Car-H_), 2983 (ν_Car-H_), 2931 (ν_C-H_), 1694 (ν_C = O_), 1557 (ν_Car. = Car._), 1507 (δ_NH_), 1477, 1407 (δ_CH_); UV-Vis (96% ethanol): λ_max_ = 393 nm, ε(λ_max)_ = 16,148 M^−1^ cm^−1^; LC-HRMS (*m*/*z*): [M + H]^+^ for C_17_H_17_N_5_O_3_ calcd. 340.1404, found 340.1402, [M + Na]^+^ for C_17_H_17_N_5_O_3_ calcd. 362.1224, found 362.1216.

All spectra are shown in the Supplementary Materials (Figures S8–S14, S43, S49 and S55).

**5-((3-Methyl-1*H*-pyrazol-5-yl)diazenyl)quinolin-8-ol (4a)**

Orange-yellow solid, yield: 80.3%, m.p.: 242–243 °C; ^1^H NMR: δ_H_ (DMSO-*d6*, 500 MHz, ppm): (DMSO-*d6*, 500 MHz, ppm): 13.12 (s, 1H, NH), 10.8 (s.l., 1H, OH), 9.25 (d, 1H, *J* = 8.2 Hz, 4-H), 9.00 (dd, 1H, *J*_1_ = 4.0 Hz, *J*_2_ = 1.4 Hz, 2-H), 7.86 (d, 1H, *J* = 8.6 Hz, 6-H), 7.75 (dd, 1H, *J*_1_ = 8.5 Hz, *J*_2_ = 4.1 Hz, 3-H), 7.24 (d, 1H, *J* = 8.3 Hz, 7-H), 6.52 (s, 1H, 5′-H), 2.33 (s, 3H, 4′-C-CH_3_); ^13^C NMR: δ_C_ (DMSO-*d6*, 125 MHz, ppm): 164.6 (1′-C), 157.0 (8-C), 148.8 (2-C), 140.1 (4′-C), 138.7 (8a-C), 137.8 (4a-C), 131.7 (4-C), 127.1 (5-C), 122.9 (3-C), 114.2 (6-C), 111.6 (7-C), 92.5 (5′-C), 15.1 (4′-C-CH_3_); ^15^N NMR: δ_N_ (DMSO-*d6*, 50.66 MHz, ppm): 299.0 (1-N), 209.2 (2′-N); FT-IR (cm^−1^): 3099 (ν_Car-H_), 1572 (δ_NH_), 1504 (δ_C-NH_), 1475 (δ_CH_); UV-Vis (96% ethanol): λ_max_ = 386 nm, ε(λ_max)_ = 20,691 M^−1^ cm^−1^; LC-HRMS (*m*/*z*): [M + H]^+^ for C_13_H_11_N_5_O calcd. 254.1036, found 254.1031, [M + Na]^+^ for C_13_H_11_N_5_O calcd. 276.0856, found 276.0839.

All spectra are shown in the Supplementary Materials (Figures S15–21, S44, S50 and S56).

**2-Methyl-5-((3-methyl-1*H*-pyrazol-5-yl)diazenyl)quinolin-8-ol (4b)**

Orange-yellow solid, yield: 78%, m.p.: 190–192 °C; ^1^H NMR: δ_H_ (DMSO-*d6*, 500 MHz, ppm): 13.1 (s, 1H, NH), 9.15 (d, 1H, *J* = 7.2 Hz, 4-H), 7.87 (d, 1H, *J* = 8.5 Hz, 6-H), 7.62 (d, 1H, *J* = 8.5 Hz, 3-H), 7.20 (d, 1H, *J* = 8.3 Hz, 7-H), 6.49 (s, 1H, 5′-H), 2.76 (s, 3H, 2-C-CH_3_), 2.33 (s, 3H, 4′-C-CH_3_); ^13^C NMR: δ_C_ (DMSO-*d6*, 125 MHz, ppm): 164.7 (1′-C), 157.5 (2-C), 156.2 (8-C), 140.2 (4′-C), 139.2 (5-C), 137.2 (8a-C), 131.7 (4-C), 125.4 (4a-C), 123.7 (3-C), 113.2 (6-C), 111.4 (7-C), 92.5 (5′-C), 24.6 (2-C-CH_3_), 10.6 (4′-C-CH_3_); ^15^N NMR: δ_N_ (Py-*d5*, 50.66 MHz, ppm): 292.8 (1-N); 208.9 (2′-N); FT-IR (cm^−1^): 3418 (ν_OH_), 3201 (ν_Car-H_), 3137 (ν_Car-H_), 2978 (ν_CH_), 1567 (δ_NH_), 1476 (δ_CH_); UV-Vis (96% ethanol): λ_max_ = 387 nm, ε(λ_max)_= 30,400 M^−1^ cm^−1^; LC-HRMS (*m*/*z*): [M + H]^+^ for C_14_H_13_N_5_O calcd. 268.1193, found 268.1187, [M + Na]^+^ for C_14_H_13_N_5_O calcd. 290.1012, found 290.0998.

All spectra are shown in the Supplementary Materials (Figures S22–S29, S456, S51 and S57).

**5-((8-Hydroxyquinolin-5-yl)diazenyl)-3-methyl-1*H*-pyrazole-4-carboxylic acid (5a)**

Orange-brown solid, yield: 70%, m.p.: >300 °C; ^1^H NMR: δ_H_ (DMSO-*d6*, 500 MHz, ppm): 12.79 (s, 1H, -COOH); 9.15 (d, 1H, *J =* 8.2 Hz, 2-H); 8.69 (s, 1H, 4-H); 7.95 (d, 1H, *J* = 8.9 Hz, 7-H); 7.55–7.53 (m, 1H, 3-H); 6.74–6.73 (m, 1H, 6-H); 6.32 (s, 1H, -OH); 2.26 (s, 3H, -CH_3_); ^13^C NMR: δ_C_ (DMSO-*d6*, 125 MHz, ppm): 163.9 (-COOH); 146.5 (4-C); 144.7 (1′-C); 143.4 (4′-C); 141.0 (8-C); 132.9 (8a-C); 131.3 (2-C); 129.2 (4a-C); 124.01 (5-C); 122.3 (3-C); 117.3 (7-C); 113.9 (6-C); 93.0 (5′-C); 11.5 (-CH_3_); FT-IR (cm^−1^): 3415 (ν_OH_), 3201 (ν_NH_), 2836 (ν_Car-H_), 1600 (ν_Car. = Car._), 1536 (δ_NH_), 1550 (ν_Car. = Car._), 1465, 1431 (δ_CH_); UV-Vis (96% ethanol): λ_max_ = 385.5 nm, ε(λ_max)_ = 15,469 M^−1^ cm^−1^; LC-HRMS (*m*/*z*): [M + H]^+^ for C_14_H_11_N_5_O_3_ calcd. 298.0935, found 298.0934, [M + Na]^+^ for C_14_H_11_N_5_O_3_ calcd. 320.0754, found 320.0757.

All spectra are shown in the Supplementary Materials (Figures S30–S35, S46, S52 and S58).

**5-((8-Hydroxy-2-methylquinolin-5-yl)diazenyl)-3-methyl-1*H*-pyrazole-4-carboxylic acid (5b)**

Yellow-brown solid, yield: 53%, m.p.: >300 °C; ^1^H NMR: δ_H_ (Py-*d5*, 500 MHz, ppm): 9.37 (d, 1H, *J* = 8.59 Hz, 4-H), 8.33 (d, 1H, *J* = 8.34 Hz, 6-H), 7.47 (m, 2H, 7-H, 3-H), 6.84 (s, 1H, -OH), 2.60 (s, 3H, 2-C-CH_3_), 2.41 (s, 3H, 4′-C-CH_3_); ^13^C NMR: δ_C_ (Py-*d5*, 125 MHz, ppm): 165.4 (-COOH), 157.9 (8-C), 157.8 (2-C), 150.2 (4′-C), 140.3 (1′-C), 138.5 (4a-C), 135.8 (8a-C), 132.8 (4-C), 126.4 (5-C), 123.8 (7-C), 114.7 (6-C), 112.0 (3-C), 94.6 (5′-C), 24.7 (2-C-CH_3_), 11.6 (4′-C-CH_3_); ^15^N NMR: δ_N_ (Py-*d5*, 50.66 MHz, ppm): 291.1 (1-N); FT-IR (cm^−1^): 3451 (ν_OH_), 3210 (ν_NH_), 2983, 2869 (ν_CH_), 1564 (δ_NH_), 1479 (ν_C-C_), 1403 (δ_CH_); UV-Vis (96% ethanol): λ_max_ = 385.5 nm, ε(λ_max)_ = 24,818 M^−1^ cm^−1^; LC-HRMS (*m*/*z*): [M + H]^+^ for C_15_H_13_N_5_O_3_ calcd. 312.1091, found 312.1060, [M + Na]^+^ for C_15_H_13_N_5_O_3_ calcd. 334.0911, found 334.0928.

All spectra are shown in the Supplementary Materials (Figures S36–S41, S47, S53 and S59).

#### 4.2.4. Evaluation of the Biological Activities

Antifungal activity was tested against *Candida albicans* (ATCC 64124), *Aspergillus fumigatus* (ATCC 46645), and *Candida auris* (DSM21092), while antibacterial activity was evaluated against *Enterococcus faecalis* (ATCC 29212), *Staphylococcus aureus* MSSA (ATCC 29213), *Pseudomonas aeruginosa*, *Escherichia coli* (ATCC 25922), *Klebsiella pneumoniae* (DSM681), and *Acinetobacter baumannii* (DSM30007). The experiments were conducted in 384-well plates with standardized microbial suspensions, incubated at 37 °C for 24 h, and growth inhibition was measured by absorbance (or fluorescence for *A. fumigatus*) using an EnVision Multilabel Reader (PerkinElmer, Shelton, CT, USA) or a Synergy HT Multi-Mode Reader (Agilent, Santa Clara, CA, USA). The compounds were classified as active (≥70% or ≥50% inhibition, depending on the assay), inconclusive (50–70% inhibition), or inactive (<50% inhibition).

Cytotoxicity was assessed on HepG2 cells using an ATP-based cell viability assay with luminescence detection via a PHERAstar FS reader (BMG Labtech, Ortenberg, Germany) at 10 μM, with compounds showing ≥30% inhibition considered active. Data were processed using Genedata Screener V18 or normalized against controls for reliable results.

The experiments were conducted at least in duplicate in independent experiments (in triplicate for the cell viability ATP quantification assays with HepG2 cells). The reported data represent the mean value of two or more independent assays, with standard deviations ranging between ±5–10%. More details regarding all testing procedures can be found in the Supplementary Materials.

## 5. Conclusions

This study reports the successful synthesis and detailed characterization of a novel series of heterocyclic azene compounds featuring pyrazole and 8-Hq moieties. Building upon our previous research, through a strategic synthetic approach involving diazo coupling reactions, we managed to synthesize four diazene derivatives (**3a**), (**3b**), (**4a**), and (**4b**), two of which were further hydrolyzed to their corresponding carboxylic acids (**5a**) and (**5b**). The proposed structures of all synthesized compounds were confirmed through a comprehensive analysis using 1D and 2D NMR spectroscopy, FT-IR spectroscopy, and high-resolution mass spectrometry (LC-HRMS). Although UV-Vis data demonstrated absorption in the visible spectrum, the relatively low values of the calculated molar absorption coefficients suggest that these compounds are not suitable for application as conventional diazo dyes.

The preliminary biological evaluations demonstrated excellent anticancer and antifungal activities. All synthesized compounds exhibited efficacy against the Hep-G2 cancer cells. The compounds also showed notable activity against the human pathogens *C. albicans* and *C. auris*. A key structure-activity relationship was identified concerning their activity against A*. fumigatus*: only the compounds lacking a methyl group at the 2-C position of the 8-Hq moiety (**3a**), (**4a**), and (**5a**) demonstrated inhibitory effects, while the methylated derivatives (**3b**), (**4b**), and (**5b**) were inactive. This finding highlights the importance of this specific structural feature for the antifungal properties of these compounds. The promising results of this screening warrant further investigation into the medicinal chemistry potential of this new class of azene derivatives.

The results validate the chosen strategic approach of synthesizing biologically active compounds by coupling known active moieties through an accessible and cost-effective diazo coupling process. Furthermore, the synthesized compounds demonstrate potential for use as ligands in the creation of metallic complexes, which could theoretically exhibit enhanced biological activity. Future studies will involve computational chemistry methods to further elucidate the mechanisms of their biological activity.

## Figures and Tables

**Table 1:** Structural analysis of compound (**3a**) by 2D NMR, indicating the correlations which demonstrate the proposed structure (chemical shifts are reported in ppm).

HSQC ^1^H-^13^C	HMBC ^1^H-^13^C	HMBC ^1^H-^15^N	COSY ^1^H-^1^H
9.45→133.3 (4-H) (4-C)	4.47→164.8 (-CH_2_-) (-C=O)	8.97→298.8 (2-H) (1-N)	9.45→7.47 (4-H) (3-H)
8.97→149.6 (2-H) (2-C)	8.49→160.6 (6-H) (8-C)	7.52→298.8 (7-H) (1-N)	8.97→7.47 (2-H) (3-H)
8.49→118.9 (6-H) (6-C)	7.52→160.6 (7-H) (8-C)		8.56→7.52 (6-H) (7-H)
7.52→113.0 (7-H) (7-C)	9.45→149.6 (4-H) (2-C)		
7.47→123.6 (3-H) (3-C)	7.47→149.6 (3-H) (2-C)		
	8.49→140.8 (6-H) (8a-C)		
	7.52→140.8 (7-H) (8a-C)		
	9.45→139.7 (4-H) (4a-C)		
	8.97→139.7 (2-H) (4a-C)		
	7.52→139.7 (7-H) (4a-C)		
	8.97→133.3 (2-H) (4-C)		
	8.49→133.3 (6-H) (4-C)		
	8.97→128.9 (2-H) (5-C)		
	8.49→128.9 (6-H) (5-C)		
	7.47→128.9 (3-H) (5-C)		
	2.73→159.4 (-CH_3_) (4′-C)		

**Table 2:** Structural analysis of compound (**3b**) by 2D NMR, indicating the correlations which demonstrate the proposed structure (chemical shifts are reported in ppm).

HSQC ^1^H-^13^C	HMBC ^1^H-^13^C	HMBC ^1^H-^15^N	COSY ^1^H-^1^H
9.36→133.1 (4-H) (4-C)	9.36→138.6 (4-H) (8a-C)	7.50→291.1 (7-H) (1-N)	9.36→7.33 (4-H) (3-H)
8.42→117.8 (6-H) (6-C)	9.36→157.9 (4-H) (2-C)	7.33→291.1 (3-H) (1-N)	8.42→7.50 (6-H) (7-H)
7.50→112.3 (7-H) (7-C)	8.42→126.1 (6-H) (4a-C)	2.56→291.1 (2-C-CH_3_) (1-N)	
7.33→124.3 (3-H) (3-C)	8.42→159.3 (6-H) (8-C)		
2.74→13.1 (4′-C-CH_3_) (4′-C-CH_3_)	7.50→138.6 (7-H) (8a-C)		
2.56→24.6 (2-C-CH_3_) (2-C-CH_3_)	7.50→159.3 (7-H) (8-C)		
	7.33→126.1 (3-H) (4a-C)		

**Table 3:** Structural analysis of compound (**4a**) by 2D NMR, indicating the correlations which demonstrate the proposed structure (chemical shifts are reported in ppm).

HSQC ^1^H-^13^C	HMBC ^1^H-^13^C	HMBC ^1^H-^15^N	COSY ^1^H-^1^H
9.25→131.7 (4-H) (4-C)	9.25→138.7 (4-H) (8a-C)	9.00→299.0 (2-H) (1-N)	9.25→7.75 (4-H) (3-H)
9.00→148.8 (2-H) (2-C)	9.25→148.8 (4-H) (2-C)	7.75→299.0 (3-H) (1-N)	9.00→7.75 (2-H) (3-H)
7.86→114.2 (6-H) (6-C)	9.00→122.9 (2-H) (3-C)	2.33→209.2 (CH_3_) (3′-N)	7.86→7.24 (6-H) (7-H)
7.75→122.9 (3-H) (3-C)	9.00→131.7 (2-H) (4-C)		
7.24→111.6 (7-H) (7-C)	9.00→137.8 (2-H) (4a-C)		
6.52→92.5 (5′-H) (5′-C)	7.86→127.1 (6-H) (5-C)		
2.33→15.1 (CH_3_) (CH_3_)	7.86→137.8 (6-H) (4a-C)		
	7.75→127.1 (3-H) (5-C)		
	7.75→148.8 (3-H) (2-C)		
	7.24→138.7 (7-H) (8a-C)		
	2.33→140.1 (CH_3_) (4′-C)		
	2.33→92.5 (CH_3_) (5′-C)		

**Table 4:** Structural analysis of compound (**4b**) by 2D NMR, indicating the correlations which demonstrate the proposed structure (chemical shifts are reported in ppm).

HSQC ^1^H-^13^C	HMBC ^1^H-^13^C	HMBC ^1^H-^15^N	COSY ^1^H-^1^H
9.15→157.5 (4-H) (2-C)	7.87→125.4 (6-H) (4a-C)	2.76→292.8 (2-C-CH_3_) (1-N)	9.15→7.62 (4-H) (3-H)
7.87→113.2 (6-H) (6-C)	7.87→139.2 (6-H) (5-C)	2.33→208.9 (4′-C-CH_3_) (2′-N)	7.87→7.20 (6-H) (7-H)
7.62→123.7 (3-H) (3-C)	7.87→156.2 (6-H) (8-C)		
7.20→111.4 (7-H) (7-C)	7.62→125.4 (3-H) (4a-C)		
6.49→92.5 (5′-H) (5′-C)	7.62→157.5 (3-H) (2-C)		
2.76→24.6 (2-C-CH_3_) (2-C-CH_3_)	7.20→137.2 (7-H) (8a-C)		
2.33→10.6 (4′-C-CH_3_) (4′-C-CH_3_)	7.20→139.2 (7-H) (5-C)		
	7.20→156.2 (7-H) (8-C)		
	2.76→123.7 (2-C-CH_3_) (3-C)		
	2.76→157.5 (2-C-CH_3_) (2-C)		
	2.33→92.5 (4′-C-CH_3_) (5′-C)		
	2.33→140.2 (4′-C-CH_3_) (4′-C)		

**Table 5:** Structural analysis of compound (**5a**) by 2D NMR, indicating the correlations which demonstrate the proposed structure (n.d.—not determined) (chemical shifts are reported in ppm).

HSQC ^1^H-^13^C	HMBC ^1^H-^13^C	HMBC ^1^H-^15^N	COSY ^1^H-^1^H
9.15→131.3 (2-H) (2-C)	9.15→132.9 (2-H) (8a-C)	n.d.	9.15→7.55 (2-H) (3-H)
8.69→146.5 (4-H) (4-C)	9.15→141.0 (2-H) (8-C)		7.95→6.74 (7-H) (6-H)
7.95→117.3 (7-H) (7-C)	8.69→131.3 (4-H) (2-C)		
7.55→122.3 (3-H) (3-C)	8.69→122.3 (4-H) (3-C)		
6.74→113.9 (6-H) (6-C)	7.55→129.2 (3-H) (4a-C)		
2.26→11.5 (CH_3_) (CH_3_)	6.74→129.2 (6-H) (4a-C)		
	2.26→93.0 (CH_3_) (5′-C)		

**Table 6:** Structural analysis of compound (**5b**) by 2D NMR, indicating the correlations which demonstrate the proposed structure (chemical shifts are reported in ppm).

HSQC ^1^H-^13^C	HMBC ^1^H-^13^C	HMBC ^1^H-^15^N	COSY ^1^H-^1^H
9.37→132.8 (4-H) (4-C)	9.37→138.5 (4-H) (4a-C)	2.60→291.1 (2-C-CH_3_) (1-N)	9.37→7.47 (4-H) (3-H)
8.33→114.7 (6-H) (6-C)	8.33→126.4 (4-H) (5-C)		8.33→7.47 (6-H) (7-H)
7.47→123.8 (7-H) (7-C)	7.47→138.5 (3-H) (4a-C)		
7.47→112.0 (3-H) (3-C)	2.60→157.8 (2-C-CH_3_) (2-C)		
2.60→24.7 (2-C-CH_3_) (2-C-CH_3_)	2.41→94.6 (4′-C-CH_3_) (5′-C)		

**Table 7:** Mean values of the activities of the synthesized compounds against selected fungi.

Microorganism	Compound Inhibition [%]						
	Amphotericin B [26,27,28]	(3a)	(3b)	(4a)	(4b)	(5a)	(5b)
*C. albicans*	95 ± 3%	94.63	82.35	96.71	96.84	94.90	94.45
*C. auris*	90 ± 4%	99.10	97.01	98.37	98.74	97.92	99.48
*A. fumigatus*	85 ± 4%	102.07	13.71	102.14	69.65	102.54	45.10

**Table 8:** Mean values of the activities of the synthesized compounds against selected Gram-positive microorganisms.

Microorganism	Compound Inhibition [%]						
	Vancomycin [29,30]	(3a)	(3b)	(4a)	(4b)	(5a)	(5b)
*E. faecalis*	95 ± 3%	4.77	1.34	−0.97	15.51	3.97	2.05
*S. aureus*	90 ± 4%	9.35	16.50	30.99	23.42	51.29	15.20

**Table 9:** Mean values of the activities of the synthesized compounds against selected Gram-negative microorganisms.

Microorganism	Compound Inhibition [%]						
	Ciprofloxacin [31,32]	(3a)	(3b)	(4a)	(4b)	(5a)	(5b)
*P. aeruginosa*	60 ± 6%	−0.27	−13.20	7.62	1.46	6.04	−21.90
*E.coli*	80 ± 5%	−7.90	−12.16	−0.27	−2.39	−0.71	−12.16
*K. pneumoniae*	65 ± 6%	−6.40	−26.00	37.00	−24.00	36.00	7.40
*A. baumannii*	40 ± 7%	−22.85	−22.10	−15.72	−11.24	−7.34	−5.02

**Table 10:** Mean values of the activities of the synthesized compounds against Hep-G2 cells.

Cell Line	Compound Inhibition [%]						
	Etoposide [33]	(3a)	(3b)	(4a)	(4b)	(5a)	(5b)
*Cell line permanent Hep-G2*	61.7 ± 6%	65.70	46.80	69.60	59.60	71.80	52.50

## Data Availability

The original contributions presented in this study are included in the article and supplementary material. Further inquiries can be directed to the corresponding author.

## Supplementary Materials

The following supporting information can be downloaded at https://www.mdpi.com/article/10.3390/ph19010004/s1. Figure S1: ^1^H NMR spectrum of the compound (**3a**), Figure S2: ^13^C NMR spectrum of the compound (**3a**), Figure S3: COSY ^1^H-^1^H spectrum of the compound (**3a**), Figure S4: ^13^C DEPT135 spectrum of the compound (**3a**), Figure S5: HSQC ^1^H-^13^C spectrum of the compound (**3a**), Figure S6: HMBC ^1^H-^13^C spectrum of the compound (**3a**), Figure S7: HMBC ^1^H-^15^N spectrum of compound (**3a**), Figure S8: ^1^H spectrum of the compound (**3b**), Figure S9: ^13^C NMR spectrum of the compound (**3b**), Figure S10: COSY ^1^H-^1^H spectrum of the compound (**3b**), Figure S11: ^13^C DEPT 135 spectrum of the compound (**3b**), Figure S12: ^1^H-^13^C HSQC spectrum of the compound (**3b**), Figure S13: ^1^H-^13^C HMBC spectrum of the compound (**3b**), Figure S14: ^1^H-^15^N HMBC spectrum of the compound (**3b**), Figure S15: ^1^H NMR spectrum of the compound (**4a**), Figure S16: ^13^C NMR spectrum of the compound (**4a**), Figure S17: ^13^C DEPT 135 spectrum of the compound (**4a**), Figure S18: ^1^H-^1^H COSY spectrum of the compound (**4a**), Figure S19: ^1^H-^13^C HSQC spectrum of the compound (**4a**), Figure S20: ^1^H-^13^C HMBC spectrum of the compound (**4a**), Figure S21: ^1^H-^15^N HMBC spectrum of the compound (**4a**), Figure S22: ^1^H NMR spectrum of the compound (**4b**), Figure S23: ^13^C NMR spectrum of the compound (**4b**), Figure S24: ^13^C DEPT135 spectrum of the compound (**4b**), Figure S25: ^1^H-^1^H COSY spectrum of the compound (**4b**), Figure S26: ^13^C DEPT 135 spectrum of the compound (**4b**), Figure S27: ^1^H-^13^C HSQC spectrum of the compound (**4b**), Figure S28: ^1^H-^13^C HMBC spectrum of the compound (**4b**), Figure S29: ^1^H-^15^N HMBC spectrum of the compound (**4b**), Figure S30: ^1^H NMR spectrum of the compound (**5a**), Figure S31: ^13^C NMR spectrum of the compound (**5a**), Figure S32: ^13^C DEPT135 spectrum of the compound (**5a**), Figure S33: ^1^H-^1^H COSY spectrum of the compound (**5a**), Figure S34: ^1^H-^13^C HSQC spectrum of the compound (**5a**), Figure S35: ^1^H-^13^C HMBC spectrum of the compound (**5a**), Figure S36: ^1^H NMR spectrum of the compound (**5b**), Figure S37: ^13^C NMR spectrum of the compound (**5b**), Figure S38: ^1^H-^1^H COSY spectrum of the compound (**5b**), Figure S39: ^1^H-^13^C HSQC spectrum of the compound (**5b**), Figure S40: ^1^H-^13^C HMBC spectrum of the compound (**5b**), Figure S41: ^1^H-^15^N HMBC spectrum of the compound (**5b**), Figure S42: IR spectrum of the compound (**3a**), Figure S43: IR spectrum of the compound (**3b**), Figure S44: IR spectrum of the compound (**4a**), Figure S45: IR spectrum of the compound (**4b**), Figure S46: IR spectrum of the compound (**5a**), Figure S47: IR spectrum of the compound (**5b**), Figure S48: UV-Vis spectrum of the compound (**3a**), Figure S49: UV-Vis spectrum of the compound (**3b**), Figure S50: UV-Vis spectrum of the compound (**4a**), Figure S51: UV-Vis spectrum of the compound (**4b**), Figure S52: UV-Vis spectrum of the compound (**5a**), Figure S53: UV-Vis spectrum of the compound (**5b**), Figure S54: LC-HRMS data for compound (**3a**), Figure S55: LC-HRMS data for compound (**3b**), Figure S56: LC-HRMS data for compound (**4a**), Figure S57: LC-HRMS data for compound (**4b**), Figure S58: LC-HRMS data for compound (**5a**), Figure S59: LC-HRMS data for compound (**5b**), Table S1: Summary IR data for the synthesized compounds, Table S2: Summary UV-Vis data for the synthesized compounds, Table S3: Summary MS data for the synthesized compounds, Table S4: Antifungal, antibacterial and anticancer activities, assessed according to the OPENSCREEN protocols, Table S5: Biological activity values, ordered for the individual compounds.
