# Supplementary material for: Synthesis, Characterization, Antimicrobial and Anticancer Evaluation of Novel Heterocyclic Diazene Compounds Derived from 8-Quinolinol
Source: Pharmaceuticals (Basel). 2025 Dec 19;19(1):4. doi: 10.3390/ph19010004 (PMC12844814; doi:10.3390/ph19010004)
Supplement: Supplementary file 1 [file pharmaceuticals-19-00004-s001.zip › pharmaceuticals-4030684-supplementary.pdf]

Synthesis, Characterization, Antimicrobial and Anticancer Evaluation of Novel Heterocyclic Diazene Compounds Derived from 8-Quinolinol

Ion Burcă [1], Alexandra-Mihaela Diaconescu [1], Valentin Badea [1,*] and Francisc Péter [1,2]

[1] Department of Applied Chemistry and Organic and Natural Compounds Engineering, Politehnica University Timisoara, Vasile Pârvan 6 Blvd., 300223 Timisoara, Romania; ion.burca2@student.upt.ro (I.B.); alexandra.diaconescu@student.upt.ro (A.-M.D.); francisc.peter@upt.ro (F.P.)

[2] Renewable Energy Research Institute-ICER, Politehnica University Timișoara, Gavril Musicescu Str. 138, 300501 Timișoara, Romania

* Correspondence: valentin.badea@upt.ro; Tel.: +40-742-044-969

Supplementary material

Contents

1. Detailed description of the biological activity assay methods used by the OPENSCREEN platform (freely available for the users) ……………………………………………………………………………4

2. Spectroscopy and chromatography data

1D and 2D NMR spectra for compounds (3a), (3b), (4a), (4b), (5a), (5b) 13

IR spectra for compounds (3a), (3b), (4a), (4b), (5a), (5b) 33

UV-Vis spectra for compounds (3a), (3b), (4a), (4b), (5a), (5b) 39

LC-HRMS data for compounds (3a), (3b), (4a), (4b), (5a), (5b) 43

3. Biological activity evaluation data for compounds (3a), (3b), (4a), (4b), (5a), (5b) 47

Figures

Figure S1 ^{1}H NMR spectrum of the compound **(3a)** 13
Figure S2 ^{13}C NMR spectrum of the compound **(3a)** 14
Figure S3 COSY ^{1}H-^{1}H spectrum of the compound **(3a)** 15
Figure S4 13C DEPT135 spectrum of the compound **(3a)** 16
Figure S5 HSQC ^{1}H-^{13}C spectrum of the compound **(3a)** 17
Figure S6 HMBC ^{1}H-^{13}C spectrum of the compound **(3a)** 18
Figure S7 HMBC 1H-15N spectrum of compound **(3a)** 18
Figure S8 ^{1}H spectrum of the compound **(3b)** 19
Figure S9 ^{13}C NMR spectrum of the compound **(3b)** 19
Figure S10 COSY ^{1}H-^{1}H spectrum of the compound **(3b)** 19
Figure S11 ^{13}C DEPT 135 spectrum of the compound **(3b)** 20
Figure S12 ^{1}H-^{13}C HSQC spectrum of the compound **(3b)** 20
Figure S13 ^{1}H-^{13}C HMBC spectrum of the compound **(3b)** 20
Figure S14 ^{1}H-^{15}N HMBC spectrum of the compound **(3b)** 21
Figure S15 ^{1}H NMR spectrum of the compound **(4a)** 21
Figure S16 ^{13}C NMR spectrum of the compound **(4a)** 22
Figure S17 ^{13}C DEPT 135 spectrum of the compound **(4a)** 22
Figure S18 ^{1}H-^{1}H COSY spectrum of the compound **(4a)** 22
Figure S19 ^{1}H-^{13}C HSQC spectrum of the compound **(4a)** 23
Figure S20 ^{1}H-^{13}C HMBC spectrum of the compound **(4a)** 23
Figure S21 ^{1}H-^{15}N HMBC spectrum of the compound **(4a)** 24
Figure S22 ^{1}H NMR spectrum of the compound **(4b)** 24
Figure S23 ^{13}C NMR spectrum of the compound **(4b)** 25
Figure S24 ^{13}C DEPT135 spectrum of the compound **(4b)** 25
Figure S25 ^{1}H-^{1}H COSY spectrum of the compound **(4b)** 25
Figure S26 ^{13}C DEPT 135 spectrum of the compound **(4b)** 26
Figure S27 ^{1}H-^{13}C HSQC spectrum of the compound **(4b)** 26
Figure S28 ^{1}H-^{13}C HMBC spectrum of the compound **(4b)** 26
Figure S29 ^{1}H-^{15}N HMBC spectrum of the compound **(4b)** 27
Figure S30 ^{1}H NMR spectrum of the compound **(5a)** 27
Figure S31 ^{13}C NMR spectrum of the compound **(5a)** 28
Figure S32 ^{13}C DEPT135 spectrum of the compound **(5a)** 28
Figure S33 ^{1}H-^{1}H COSY spectrum of the compound **(5a)** 29
Figure S34 ^{1}H-^{13}C HSQC spectrum of the compound **(5a)** 29
Figure S35 ^{1}H-^{13}C HMBC spectrum of the compound **(5a)** 30
Figure S36 ^{1}H NMR spectrum of the compound **(5b)** 31
Figure S37 ^{13}C NMR spectrum of the compound **(5b)** 31
Figure S38 ^{1}H-^{1}H COSY spectrum of the compound **(5b)** 31
Figure S39 ^{1}H-^{13}C HSQC spectrum of the compound **(5b)** 32
Figure S40 ^{1}H-^{13}C HMBC spectrum of the compound **(5b)** 32
Figure S41 ^{1}H-^{15}N HMBC spectrum of the compound **(5b)** 32
Figure S42 IR spectrum of the compound **(3a)** 33
Figure S43 IR spectrum of the compound **(3b)** 34
Figure S44 IR spectrum of the compound **(4a)** 35
Figure S45 IR spectrum of the compound **(4b)** 36
Figure S46 IR spectrum of the compound **(5a)** 37

Figure S47 IR spectrum of the compound **(5b)** 38
Figure S48 UV-Vis spectrum of the compound **(3a)** 39
Figure S49 UV-Vis spectrum of the compound **(3b)** 40
Figure S50 UV-Vis spectrum of the compound **(4a)** 40
Figure S51 UV-Vis spectrum of the compound **(4b)** 41
Figure S52 UV-Vis spectrum of the compound **(5a)** 41
Figure S53 UV-Vis spectrum of the compound **(5b)** 42
Figure S54 LC-HRMS data for compound **(3a)** 43
Figure S55 LC-HRMS data for compound **(3b)** 43
Figure S56 LC-HRMS data for compound **(4a)** 44
Figure S57 LC-HRMS data for compound **(4b)** 44
Figure S58 LC-HRMS data for compound **(5a)** 44
Figure S59 LC-HRMS data for compound **(5b)** 45

Tables

Table S1 Summary IR data for the synthesized compounds 39
Table S2 Summary UV-Vis data for the synthesized compounds 42
Table S3 Summary MS data for the synthesized compounds 45
Table S4. Antifungal, antibacterial and anticancer activities, assessed according to the OPENSCREEN protocols........ 47
Table S5. Biological activity values, ordered for the individual compounds 48

1. Methods: detailed description of the biological activity assay methods used by the OPENSCREEN platform

1. ***Candida albicans*** **ATCC 64124 Anti-Fungal Assay**

Screening of the 3rd batch of the ACADEMIC Library (1408 compounds) for 1 concentration (50 µM) with 2 replicates against *C. albicans* ATCC64124, according to "N8: Anti-Bacterial and Anti-Fungal Assays Bioprofiling Handbook" and following the protocols optimized during the validation step indicated in that proposal (Under the frame of the EU-OS bioprofiling tender process), to determine the antibacterial properties of these compounds against this human pathogen. Briefly, compounds were tested in a liquid growth medium dispensed in 384 well plates inoculated with a standardized fungal suspension and after overnight incubation at 37ºC the plates were examined for bacterial growth as absorbance readout. The Genedata Screener software (Genedata, Inc., Basel, Switzerland) was used to process and analyze all the data from the screen. The reproducibility and sensitivity were supported by the statistical values derived from all the experiments performed. The activity of the compounds was expressed as a percentage of growth inhibition where 100% represented the inhibition growth of the target microorganism and 0% represented the total growth of target microorganism on the assay.

Assay setup

Assay stage: primary assay

Bioassay: cell growth assay

Bioassay type: functional phenotypic

Bioassay setting: in vitro

BAO tags: high throughput screening, research institute, researcher, screening lab investigator, antifungal drug

Screening site: Fundación MEDINA - Screening and target validation, Microbiology and Chemistry

Detection method

Physical detection method: absorbance

Detection instrument: EnVision Multilabel Reader

Target type: Organism

Activities

Concentration unit: micromolar

Activity determination method: "Value" column is the activity of the compound calculated as an average of the percentage of inhibition. "Active" is defined as a value equal or greater than 70 (≥ 70). "Inconclusive" is defined as a value less than 70 (< 70) and equal or greater than 50 (≥ 50). "Inactive" is defined as a value less than 50 (< 50). A column with the calculated value of the standard deviation has been added to roughly estimate the reliability of the data .

2. ***Aspergillus fumigatus*** **ATCC 46645 Anti-Fungal Assay**

Screening of the 3rd batch of the ACADEMIC Library (1408 compounds) for 1 concentration (50 µM) with 2 replicates against A. fumigatus ATCC46645, according to "N8: Anti-Bacterial and Anti-Fungal Assays Bioprofiling Handbook" and following the protocols optimized during the validation step

indicated in that proposal (Under the frame of the EU-OS bioprofiling tender process), to determine the antifungal properties of these compounds against this human pathogen. Briefly, compounds were tested in a liquid growth medium dispensed in 384 well plates inoculated with a standardized fungal suspension and after overnight incubation at 37ºC the plates were examined for fungal growth as fluorescence readout. The Genedata Screener software (Genedata, Inc., Basel, Switzerland) was used to process and analyze all the data from the screen. The reproducibility and sensitivity were supported by the statistical values derived from all the experiments performed. The activity of the compounds was expressed as a percentage of growth inhibition where 100% represented the inhibition growth of the target microorganism and 0% represented the total growth of target microorganism on the assay.

Assay setup

Assay stage: primary assay

Bioassay: cell growth assay

Bioassay type: functional phenotypic

Bioassay setting: in vitro

BAO tags: high throughput screening, research institute, researcher, screening lab investigator, antifungal drug

Screening site: Fundación MEDINA - Screening and target validation, Microbiology and Chemistry

Detection method

Physical detection method: absorbance

Detection instrument: EnVision Multilabel Reader

Target type: Organism

Activities

Concentration unit: micromolar

Activity determination method: "Value" column is the activity of the compound calculated as an average of the percentage of inhibition. "Active" is defined as a value equal or greater than 70 (≥ 70). "Inconclusive" is defined as a value less than 70 (< 70) and equal or greater than 50 (≥ 50). "Inactive" is defined as a value less than 50 (< 50). A column with the calculated value of the standard deviation has been added to roughly estimate the reliability of the data .

3. *Candida auris* DSM21092 Anti-Fungal Assay

Screening of the 3 rd batch of the Academic Library (1408 compounds) for 1 concentration (50 µM) with 2 replicates against C. auris DSM21092, according to "N8: Anti-Bacterial and Anti-Fungal Assays Bioprofiling Handbook" and following the protocols optimized during the validation step indicated in that proposal (Under the frame of the EU-OS bioprofiling tender process), to determine the antifungal properties of these compounds against this human pathogen. Briefly, compounds were tested in a liquid growth medium dispensed in 384 well plates inoculated with a standardized fungal suspension and after overnight incubation at 37ºC the plates were examined for fungal growth as absorbance readout. The Genedata Screener software (Genedata, Inc., Basel, Switzerland) was used to process and analyze all the data from the screen. The reproducibility and sensitivity were supported by the statistical values derived from all the experiments performed. The activity of the compounds was

expressed as a percentage of growth inhibition where 100% represented the inhibition growth of the target microorganism and 0% represented the total growth of target microorganism on the assay.

Assay setup

Assay stage: primary assay

Bioassay: cell growth assay

Bioassay type: functional phenotypic

Bioassay setting: in vitro

BAO tags: high throughput screening, research institute, researcher, screening lab investigator, antifungal drug

Screening site: Fundación MEDINA - Screening and target validation, Microbiology and Chemistry

Detection method

Physical detection method: absorbance

Detection instrument: EnVision Multilabel Reader

Target type: Organism

Activities

Concentration unit: micromolar

Activity determination method: "Value" column is the activity of the compound calculated as an average of the percentage of inhibition. "Active" is defined as a value equal or greater than 70 (≥ 70). "Inconclusive" is defined as a value less than 70 (< 70) and equal or greater than 50 (≥ 50). "Inactive" is defined as a value less than 50 (< 50). A column with the calculated value of the standard deviation has been added to roughly estimate the reliability of the data .

4. *Enterococcus faecalis* ATCC 29212 Anti-Bacterial Assay

Screening of the 3 rd batch of the Academic Library (1408 compounds) for 1 concentration (50 μM) with 2 replicates against Enterococcus faecalis ATCC29212, according to "N8: Anti-Bacterial and Anti-Fungal Assays Bioprofiling Handbook" and following the protocols optimized during the validation step indicated in that proposal (Under the frame of the EU-OS bioprofiling tender process), to determine the antibacterial properties of these compounds against this human pathogen. Briefly, compounds were tested in a liquid growth medium dispensed in 384 well plates inoculated with a standardized bacterial suspension (CFU/mL) and after overnight incubation at 37ºC the plates were examined for bacterial growth as absorbance readout. The Genedata Screener software (Genedata, Inc., Basel, Switzerland) was used to process and analyze all the data from the screen. The reproducibility and sensitivity were supported by the statistical values derived from all the experiments performed. The activity of the compounds was expressed as a percentage of growth inhibition where 100% represented the inhibition growth of the target microorganism and 0% represented the total growth of target microorganism on the assay.

Assay setup

Assay stage: primary assay

Bioassay: cell growth assay

Bioassay type: functional phenotypic

Bioassay setting: in vitro

BAO tags: high throughput screening, research institute, researcher, screening lab investigator, antifungal drug

Screening site: Fundación MEDINA - Screening and target validation, Microbiology and Chemistry

Detection method

Physical detection method: absorbance

Detection instrument: EnVision Multilabel Reader

Target type: Organism

Activities

Concentration unit: micromolar

Activity determination method: "Value" column is the activity of the compound calculated as an average of the percentage of inhibition. "Active" is defined as a value equal or greater than 70 (≥ 70). "Inconclusive" is defined as a value less than 70 (< 70) and equal or greater than 50 (≥ 50). "Inactive" is defined as a value less than 50 (< 50). A column with the calculated value of the standard deviation has been added to roughly estimate the reliability of the data .

5. *Staphylococcus aureus*, MSSA ATCC 29213 Anti-Bacterial Assay

Screening of the 3rd batch of the Academic Library (1408 compounds) for 1 concentration (50 µM) with 2 replicates against methicillin-susceptible Staphylococcus aureus ATCC29213, according to "N8: Anti-Bacterial and Anti-Fungal Assays Bioprofiling Handbook" and following the protocols optimized during the validation step indicated in that proposal (Under the frame of the EU-OS bioprofiling tender process), to determine the antibacterial properties of these compounds against this human pathogen. Briefly, compounds were tested in a liquid growth medium dispensed in 384 well plates inoculated with a standardized bacterial suspension (CFU/mL) and after overnight incubation at 37ºC the plates were examined for bacterial growth as absorbance readout. The Genedata Screener software (Genedata, Inc., Basel, Switzerland) was used to process and analyze all the data from the screen. The reproducibility and sensitivity were supported by the statistical values derived from all the experiments performed. The activity of the compounds was expressed as a percentage of growth inhibition where 100% represented the inhibition growth of the target microorganism and 0% represented the total growth of target microorganism on the assay.

Assay setup

Assay stage: primary assay

Bioassay: cell growth assay

Bioassay type: functional phenotypic

Bioassay setting: in vitro

BAO tags: high throughput screening, research institute, researcher, screening lab investigator, antifungal drug

Screening site: Fundación MEDINA - Screening and target validation, Microbiology and Chemistry

Detection method

Physical detection method: absorbance

Detection instrument: EnVision Multilabel Reader

Target type: Organism

Activities

Concentration unit: micromolar

Activity determination method: "Value" column is the activity of the compound calculated as an average of the percentage of inhibition. "Active" is defined as a value equal or greater than 70 (≥ 70). "Inconclusive" is defined as a value less than 70 (< 70) and equal or greater than 50 (≥ 50). "Inactive" is defined as a value less than 50 (< 50). A column with the calculated value of the standard deviation has been added to roughly estimate the reliability of the data .

6. Growth inhibition *Pseudomonas aeruginosa*

Compound plates were prepared using the acoustic dispenser Echo 550 to transfer 250 nL into each well of a 384 well plate. Assay volume was 60 µL of the bacterial suspension, which was prepared from an overnight culture. Plates were incubated at 37°C for 24h. Growth was followed via determination of the optical density at 600 nm. The values resulting from 24h were analysed.

Assay setup

Assay stage: primary assay

Bioassay: cell growth assay

Bioassay type: functional phenotypic

Bioassay setting: in vitro

Screening site: Helmholtz Centre for Infection Research - Department of Chemical Biology

Detection method

Physical detection method: absorbance

Detection instrument: Synergy HT Multi-Mode Reader (BioTek)

Target type: Organism

Activities

Concentration unit: micromolar

Activity determination method: Optical density was normalized as growth inhibition with respect to control samples. Compounds are active, when they resulted in more than 50% growth inhibition.

7. Inhibition of *Escherichia coli*

The growth inhibitory potential of compounds for *E.coli* ATCC 25922 is detected via the turbidity of the solutions at 600 nm (OD600).

Assay setup

Assay stage: primary assay

Bioassay: cell growth assay

Bioassay type: functional phenotypic

Bioassay setting: in vitro

Screening site: Helmholtz Centre for Infection Research - Department of Chemical Biology

Detection method

Physical detection method: transmittance

Detection instrument: Synergy HT Multi-Mode Reader (BioTek)

Target type: Organism

Activities

Concentration unit: micromolar

Activity determination method: Data are normalised with respect to controls as % growth inhibition. Compounds are defined as active, when the growth is inhibited to more than 50%.

8. Growth inhibition of *Klebsiella pneumonia*

Klebsiella pneumonia, DSM681 = ATCC 10031, was cultivated in MHB in 384 well plates. 250nL of 10 mM solutions of each compound in DMSO were transferred with the acoustic dispenser Echo 550 to each well. The cultivation volume was 60 µL, plates were incubated at 37°C for 24h. The optical density at 600nm was determined and used as measure for bacterial growth. Data from control wells with medium only were used as 100% inhibition, and data from control wells with bacteria only were set as 0% growth inhibition. 50% growth inhibition was chosen as threshold to identify active compounds.

Assay setup

Assay stage: primary assay

Bioassay: cell growth assay

Bioassay type: functional phenotypic

Bioassay setting: in vitro

Screening site: Helmholtz Centre for Infection Research - Department of Chemical Biology

Detection method

Physical detection method: absorbance

Detection instrument: Synergy HT Multi-Mode Reader (BioTek)

Target type: Organism

Activities

Concentration unit: micromolar

Activity determination method: primary data are single point measurements, and activity is defined as at least 50% growth inhibition after 24h.

9. Growth inhibition of *Acinetobacter baumanii*

Acinetobacter baumanii, DSM30007, was cultivated in TSY in 384 well plates. 250nL of 10 mM solutions of each compound in DMSO were transferred with the acoustic dispenser Echo 550 to each well. The cultivation volume was 60 µL, plates were incubated at 37°C for 24h. The optical density at 600nm was determined and used as measure for bacterial growth. Data from control wells with medium only were used as 100% inhibition, and data from control wells with bacteria only were set as 0% growth inhibition. 50% growth inhibition was chosen as threshold to identify active compounds.

Assay setup

Assay stage: primary assay

Bioassay: cell growth assay

Bioassay type: functional phenotypic

Bioassay setting: in vitro

Screening site: Helmholtz Centre for Infection Research - Department of Chemical Biology

Detection method

Physical detection method: absorbance

Detection instrument: Synergy HT Multi-Mode Reader (BioTek)

Target type: Organism

Activities

Concentration unit: micromolar

Activity determination method: primary data are single point measurements, and activity is defined as at least 50% growth inhibition after 24h.

10. Cell viability ATP quantification assay with HepG2 cells

Plate type: 384-well plate Corning 3764 Black clear bottom. 750 cells /well seeded into the plate. Cell culture medium: DMEM (1.0 g/L glucose, without L-Glutamine, Lonza 12-707F), 10% FBS (Gibco 10270106), 2 mM L-glutamine (Lonza BE17-605E), 2% Pen/Strep (Lonza DE17-602E), + 1% NEAA (Gibco 11140-035) 48 hour incubation time. Cell viability ATP quantification assay with HepG2 cells. Assay kit: CellTiter-Glo® 2.0 Cell Viability Assay - Promega Corporation. 15uL CellTiter-Glo/well added. Screening concentration was 10 uM. Luminescence was detected using Pherastar FS reader. Data was normalized to the low (10 uM Sepantronium bromide - YM155) and high controls (25 nL/well Dimethyl Sulfoxide - DMSO) to generate percent inhibition values. The percent inhibition values uploaded.

Assay setup

Assay stage: primary assay

Bioassay: cell viability ATP quantitation assay

Bioassay type: functional

Bioassay setting: in vitro

Assay organism: Homo sapiens

BAO tags: time unit, hour, screening concentration, normalized data

Screening site: Institute for Molecular Medicine Finland - High Throughput Biomedicine Unit

Detection method

Physical detection method: luminescence method

Detection instrument: BMG PHERAstar FS plate reader (BMG Labtech, Cary NC)

Target

Target type: Cell line

Cell-line type: Permanent

Activities

Concentration unit: micromolar

Activity determination method: The compound was selected for validation if the average Percent Inhibition value (at 10 uM) was equal or greater than 30% in the primary screening.

10. Cell viability ATP quantification assay with HepG2 cells

Validation screen with 207 compounds that showed at least 30% Inhibition in the primary screen. Plate type: 384-well plate Corning 3764 Black clear bottom. 750 cells /well seeded into the plate. Cell culture medium: DMEM (1.0 g/L glucose, without L-Glutamine, Lonza 12-707F), 10% FBS (Gibco 10270106), 2 mM L-glutamine (Lonza BE17-605E), 2% Pen/Strep (Lonza DE17-602E), + 1% NEAA (Gibco 11140-035) 48 hour incubation time. Cell viability ATP quantification assay with HepG2 cells. Assay kit: CellTiter-Glo® 2.0 Cell Viability Assay - Promega Corporation. 15uL CellTiter-Glo/well added. Compounds were tested with 8 doses in triplicates in independent experiments. Screening concentrations were 0.001, 0.01, 0.1, 0.3, 1, 3, 10, 30, 50 uM. Luminescence was detected using Pherastar FS reader. Data was normalized to the low (10 uM Sepantronium bromide - YM155) and high controls (25 nL/well Dimethyl Sulfoxide - DMSO) to generate percent inhibition values. The percent inhibition values uploaded. Curve fitting was done using four parameter hill fitting algorithm. AUC, IC_{50}, low and high asymptote values are reported.

Assay setup

Assay stage: confirmatory assay

Bioassay: cell viability ATP quantitation assay

Bioassay type: functional

Bioassay setting: in vitro

Assay organism: Homo sapiens

BAO tags: time unit, hour, screening concentration, normalized data

Screening site: Institute for Molecular Medicine Finland - High Throughput Biomedicine Unit

Detection method

Physical detection method: luminescence method

Detection instrument: BMG PHERAstar FS plate reader (BMG Labtech, Cary NC)

Target

Target type: Cell line

Cell-line type: Permanent

Activities

Concentration unit: micromolar

Activity determination method: Validation screen with active compounds with average percent inhibition value (at 10 uM concentration) > 30. There were 207 compounds based on the percent inhibition values from the primary screen.

2. Spectroscopy and chromatography data

1D and 2D NMR spectra for compounds (3a), (3b), (4a), (4b), (5a), (5b)

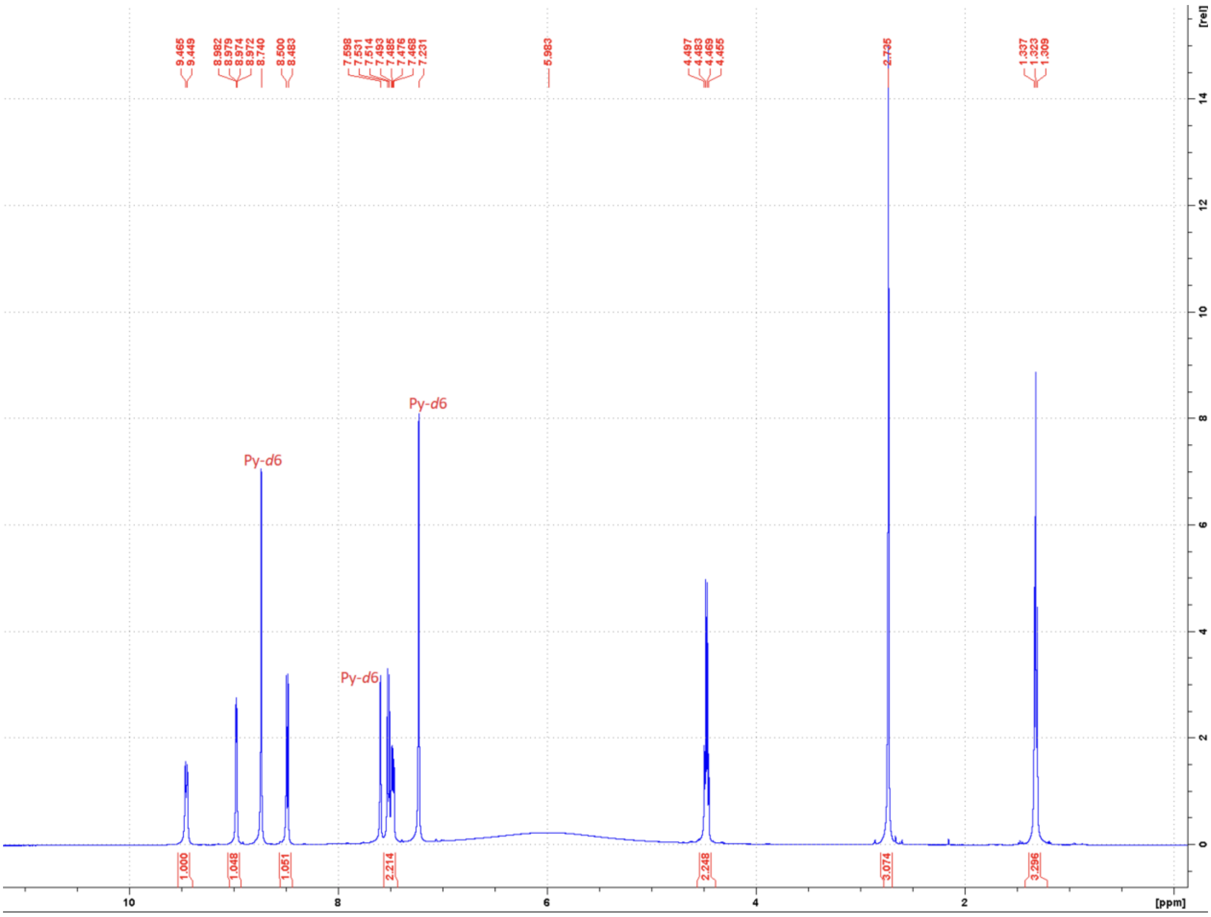

Figure S1. *^{1}H NMR spectrum of the compound* ***(3a)***

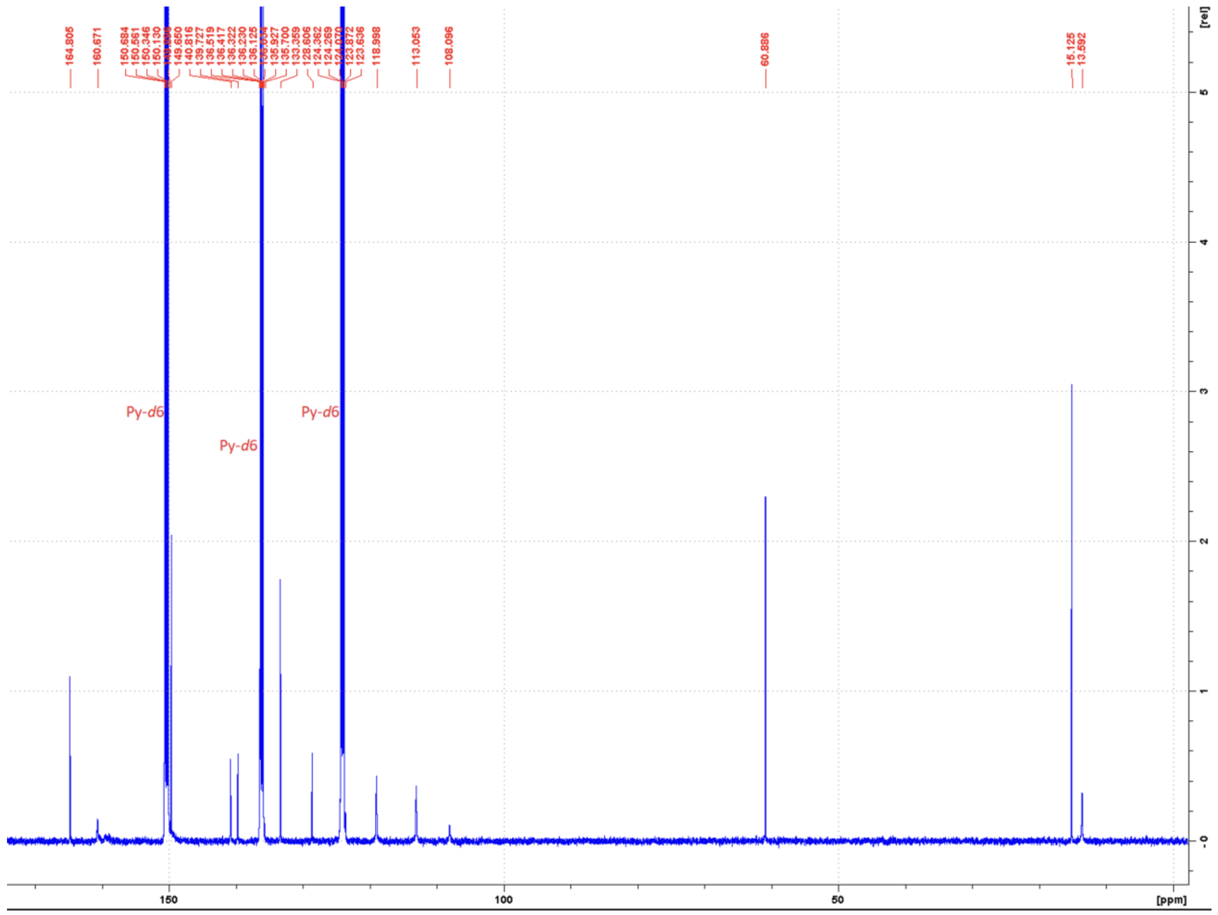

Figure S2. *^{13}C NMR spectrum of the compound* ***(3a)***

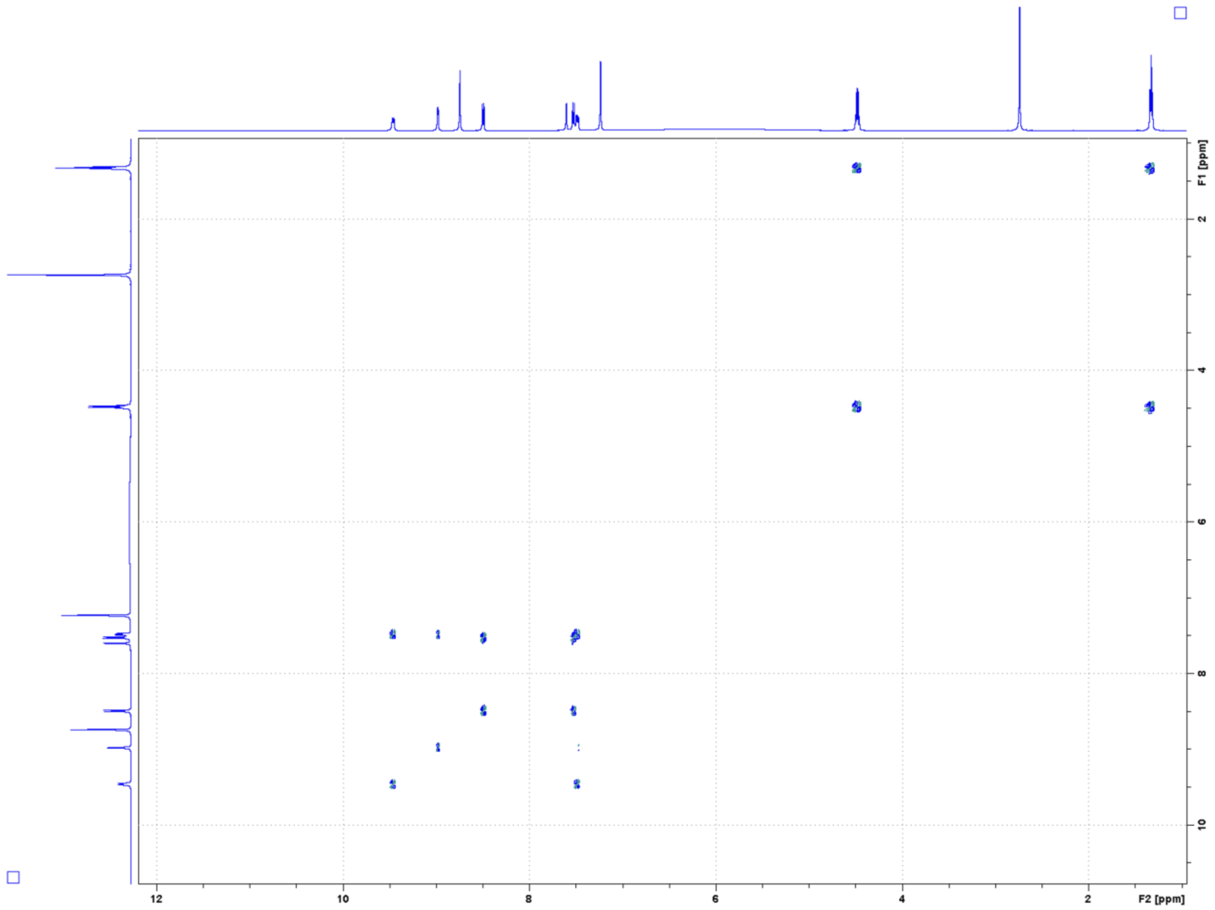

Figure S3. *COSY* ^{1}H-^{1}H *spectrum of the compound* ***(3a)***

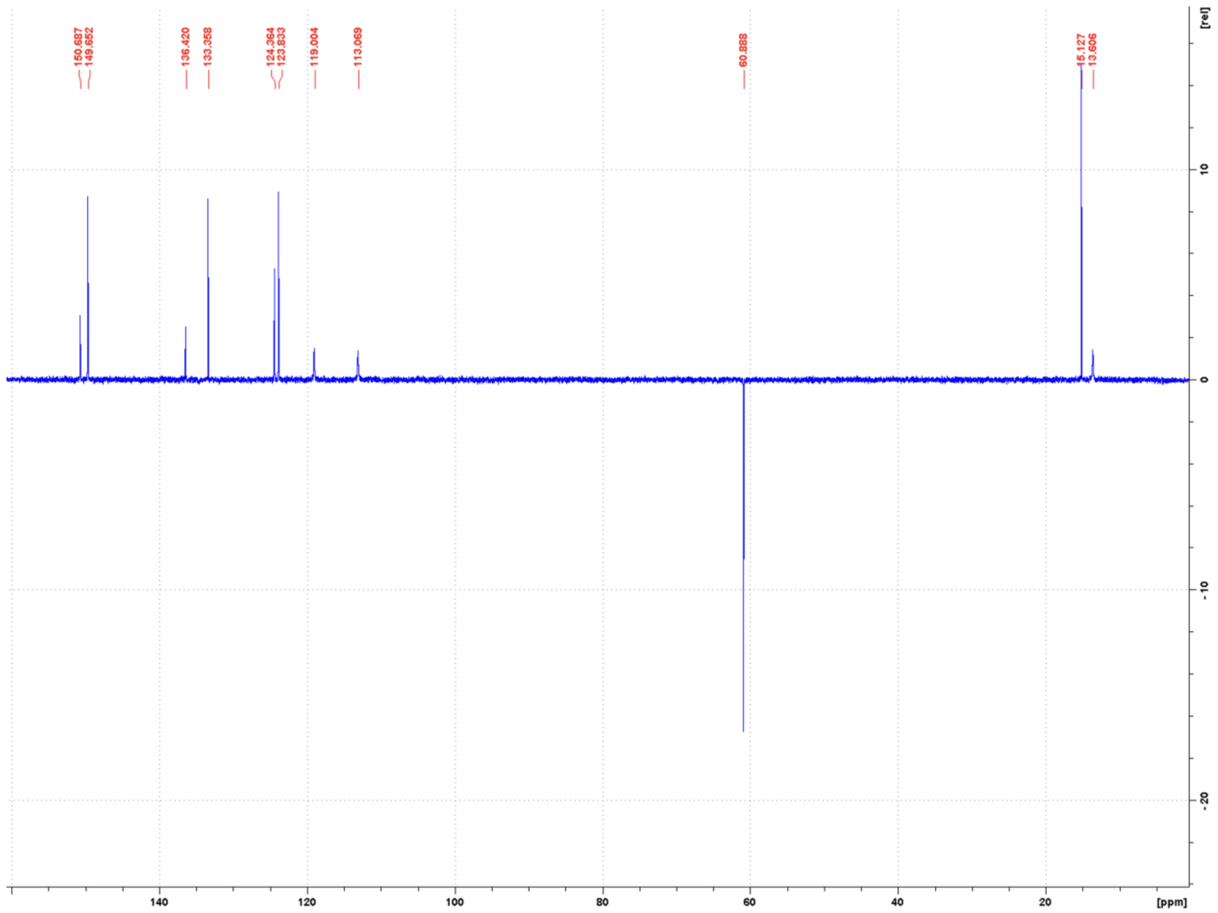

Figure S4. *13C DEPT135 spectrum of the compound* ***(3a)***

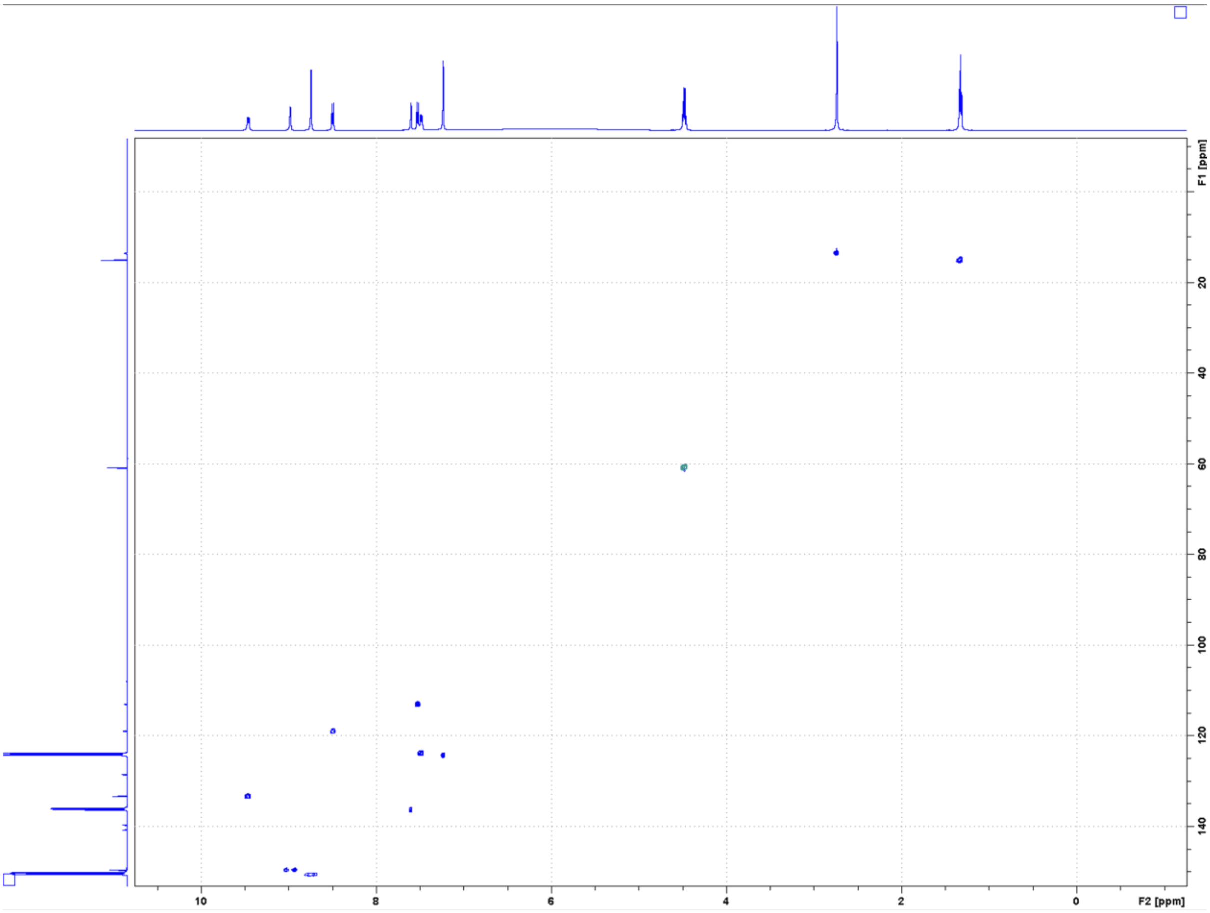

Figure S5. *HSQC* ^{1}H*-*^{13}C *spectrum of the compound* ***(3a)***

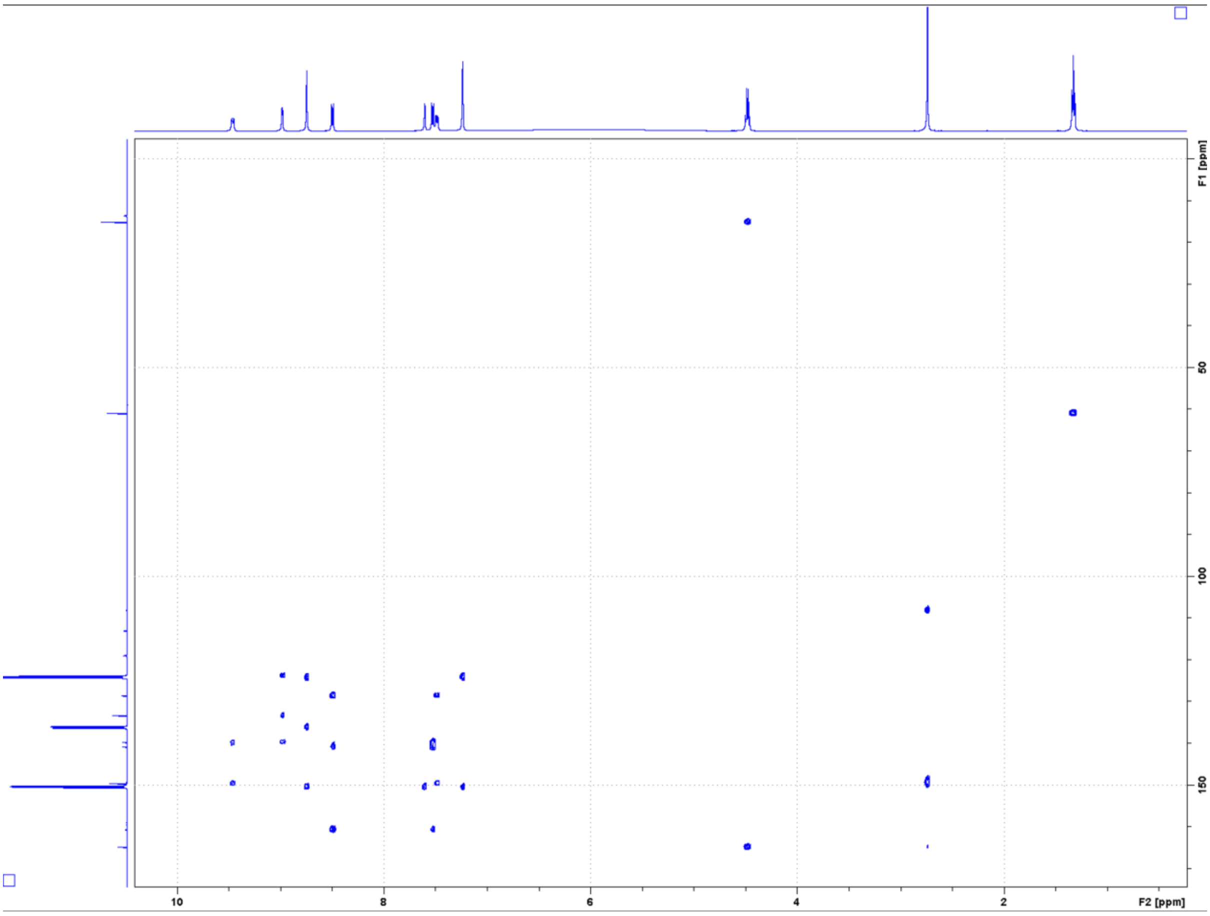

Figure S6. *HMBC ^{1}H-^{13}C spectrum of the compound* ***(3a)***

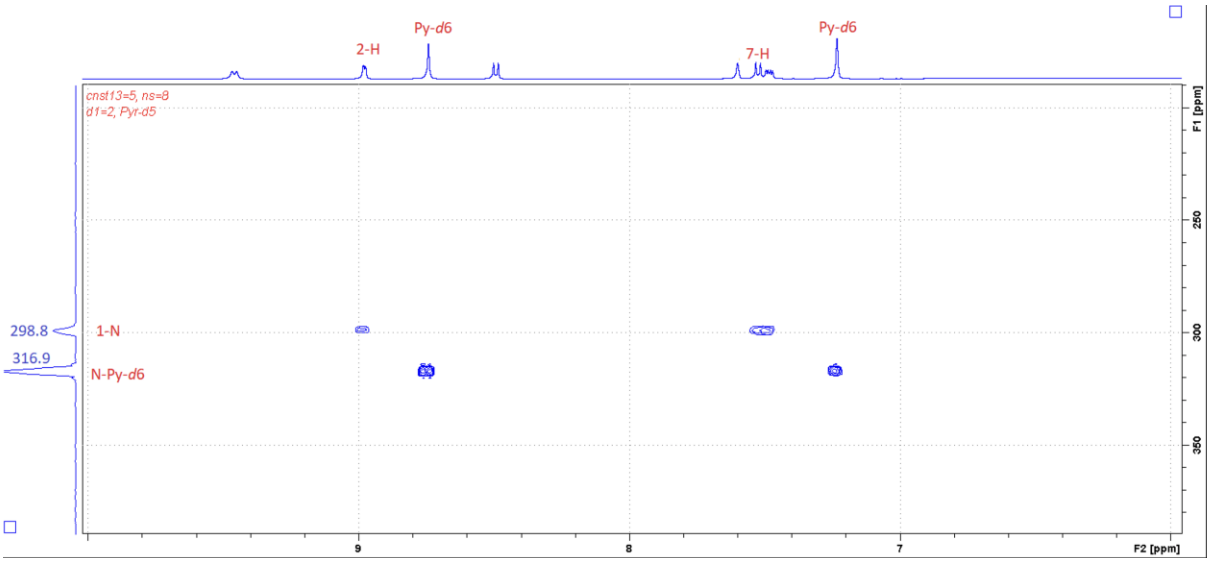

Figure S7. *HMBC 1H-15N spectrum of compound* ***(3a)***

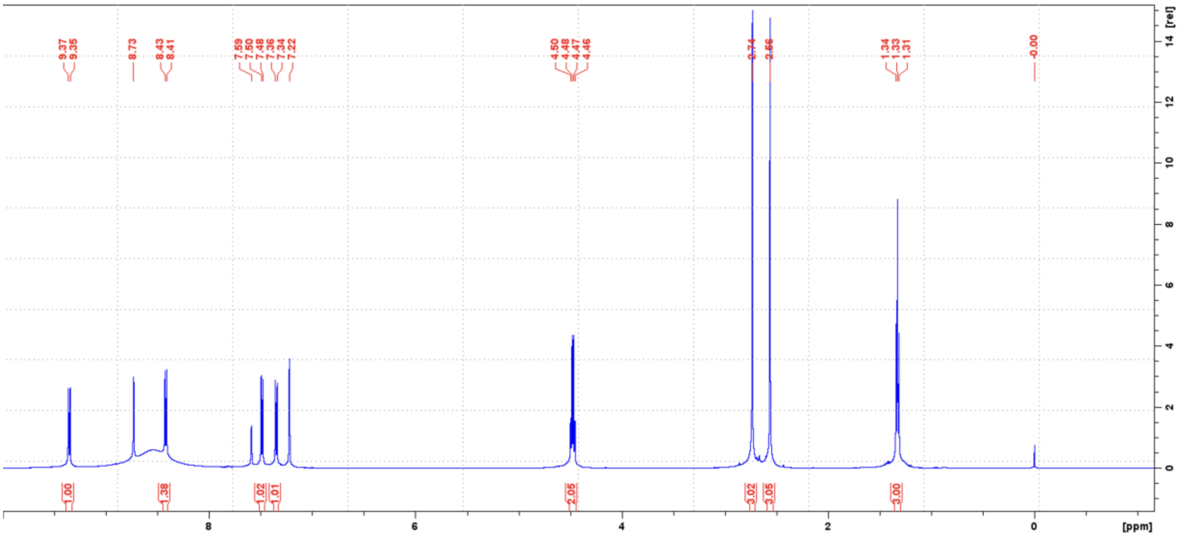

Figure S8. *^{1}H spectrum of the compound* ***(3b)***

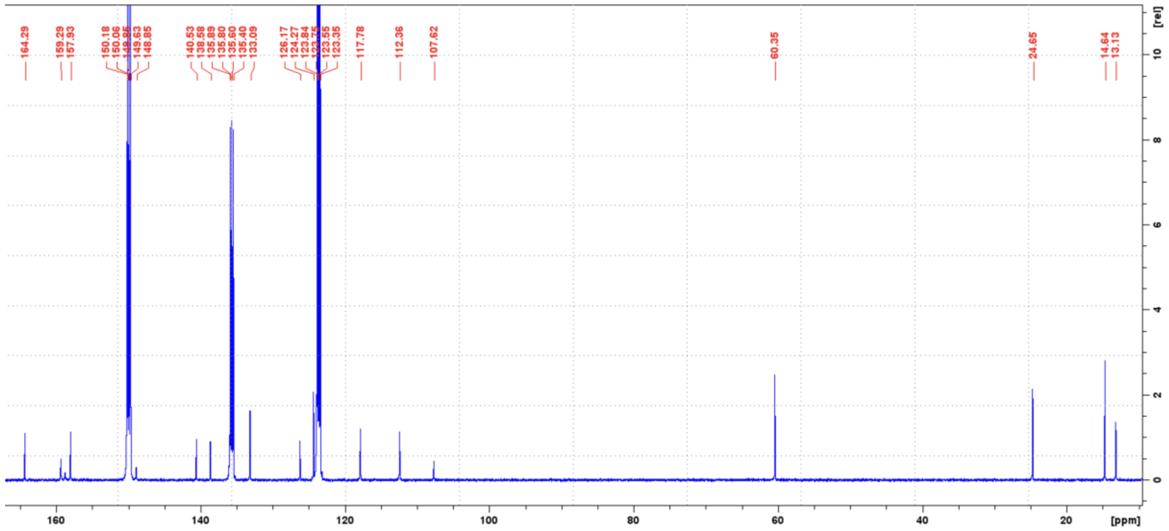

Figure S9. *^{13}C NMR spectrum of the compound* ***(3b)***

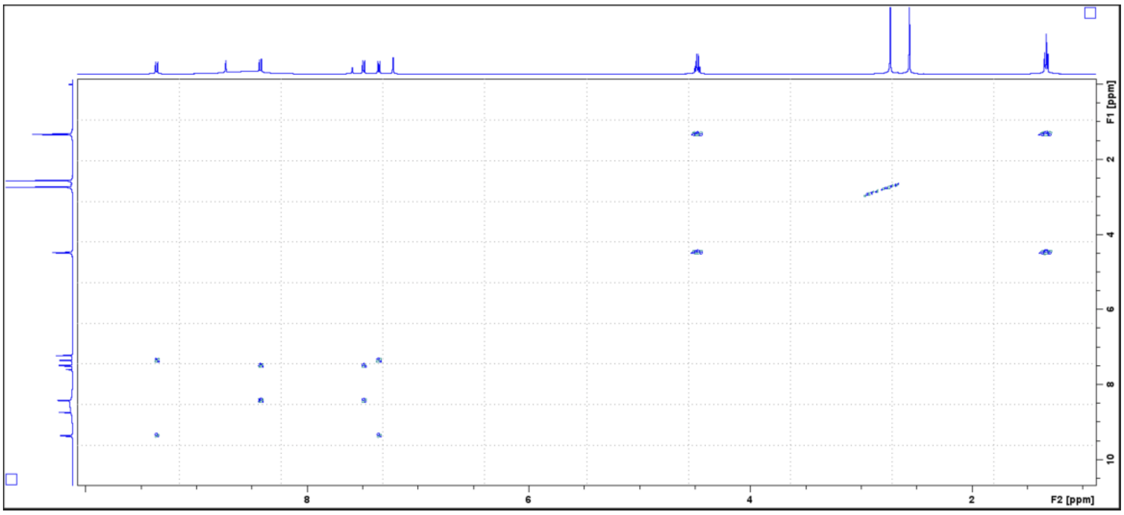

Figure S10. *COSY ^{1}H-^{1}H spectrum of the compound* ***(3b)***

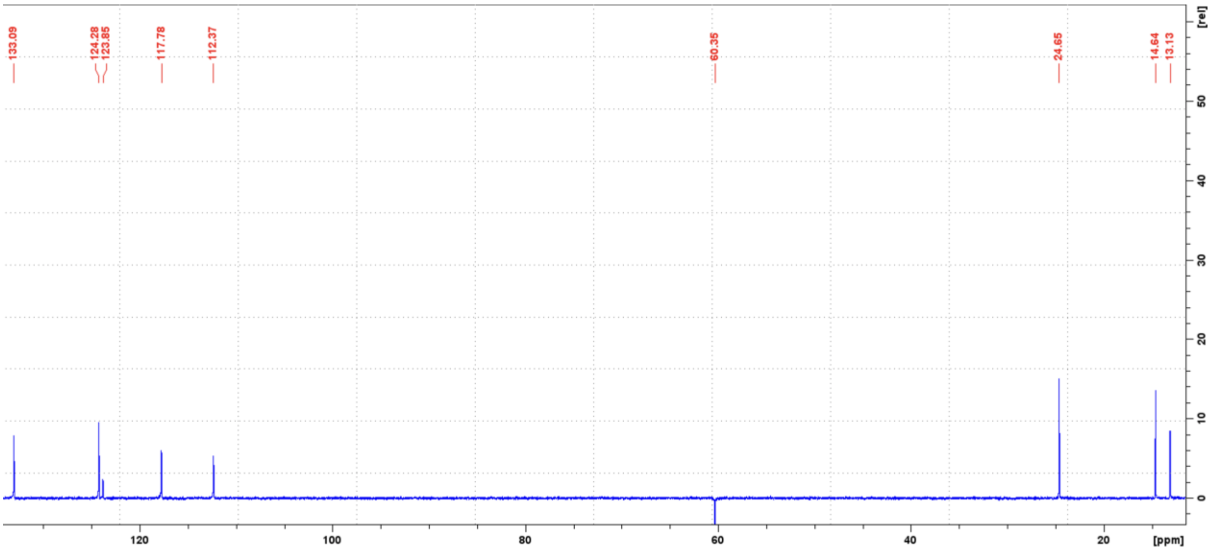

Figure S11. ^{13}C DEPT 135 spectrum of the compound **(3b)**

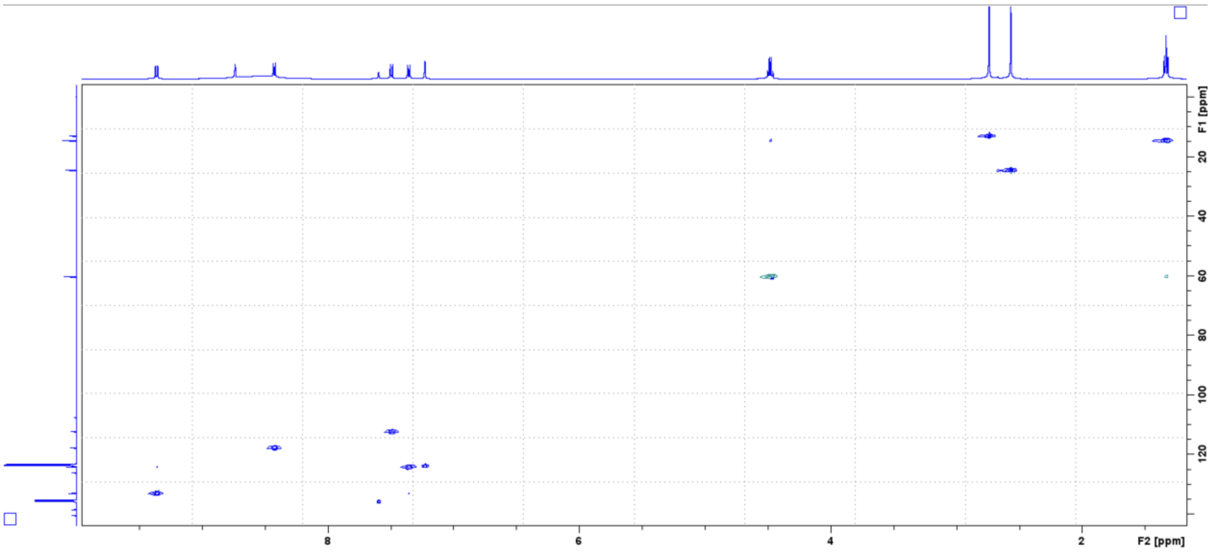

Figure S12. ^{1}H-^{13}C HSQC spectrum of the compound **(3b)**

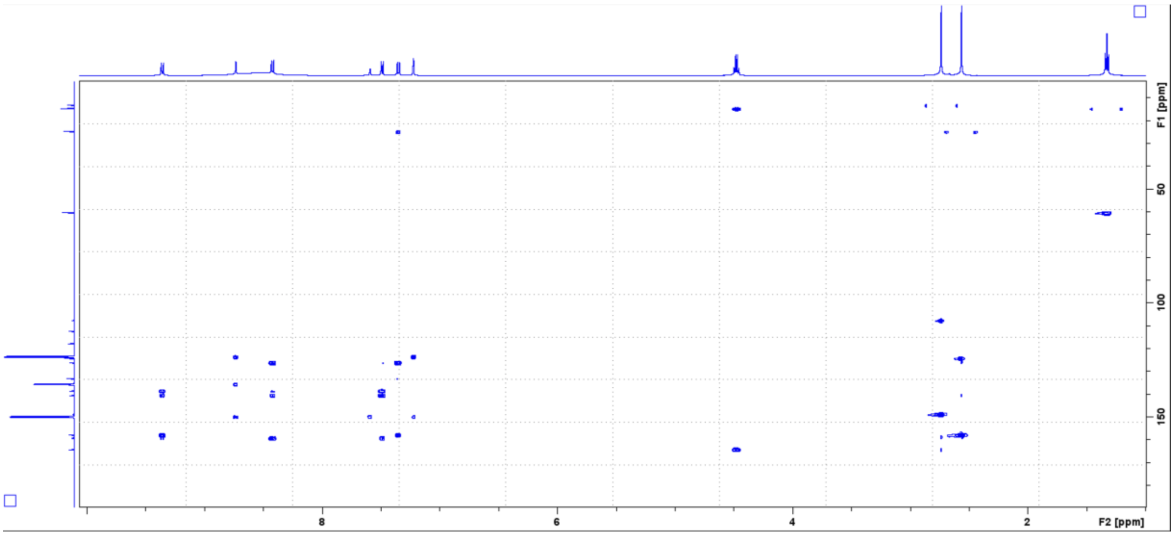

Figure S13. ^{1}H-^{13}C HMBC spectrum of the compound **(3b)**

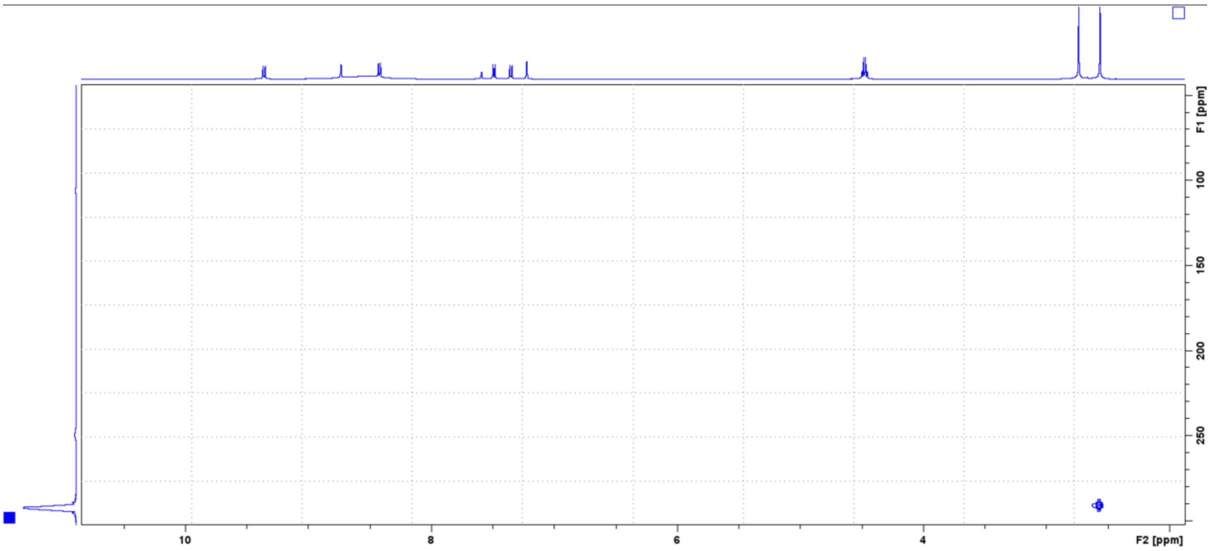

Figure S14. *¹H-¹⁵N HMBC spectrum of the compound* ***(3b)***

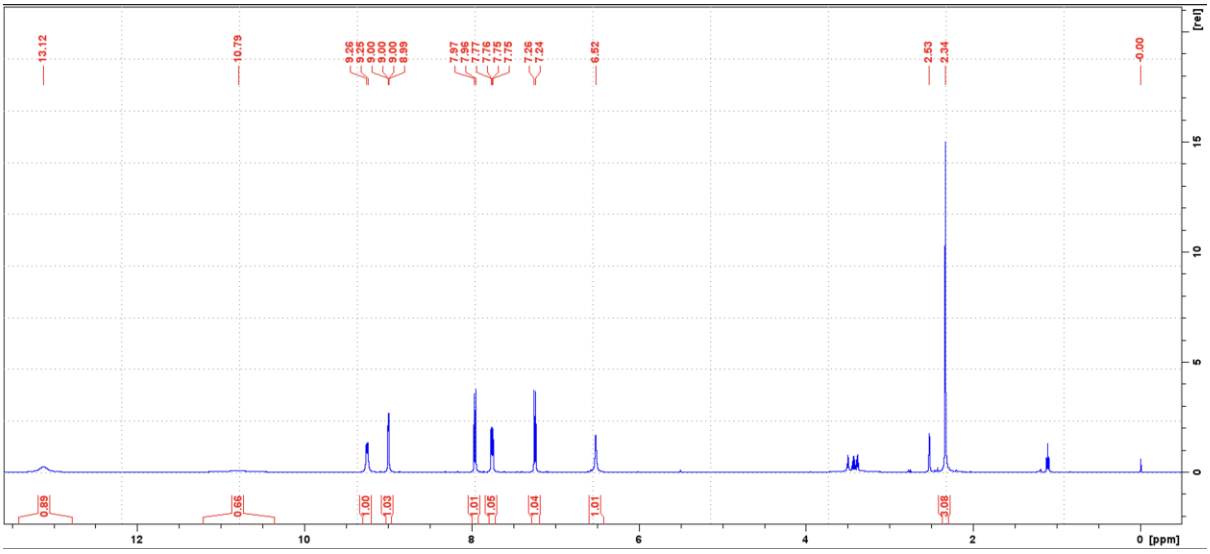

Figure S15. *¹H NMR spectrum of the compound* ***(4a)***

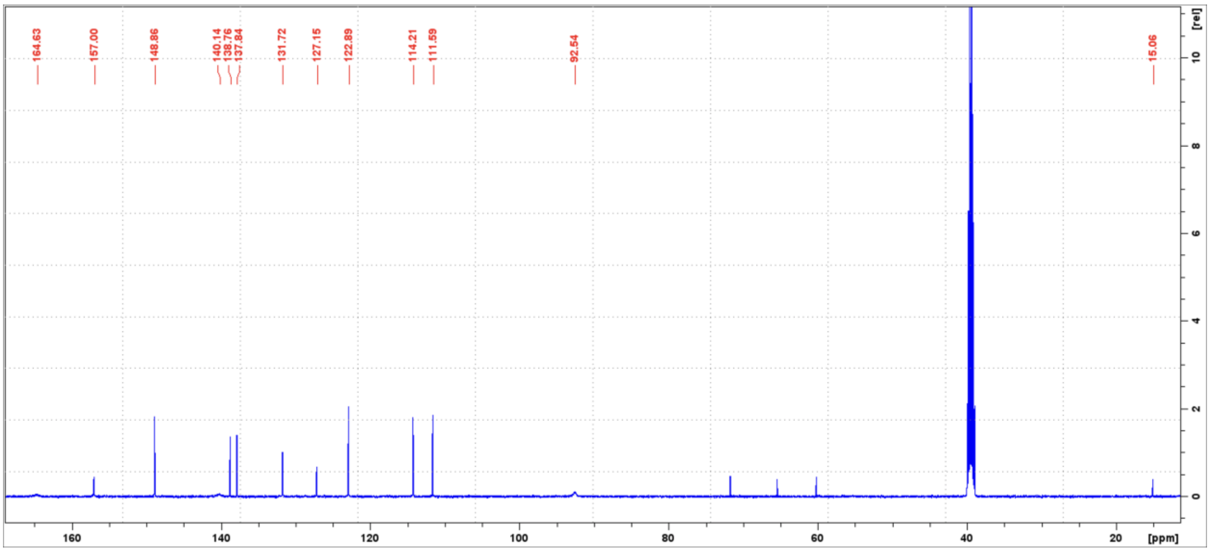

Figure S16. *^{13}C NMR spectrum of the compound* ***(4a)***

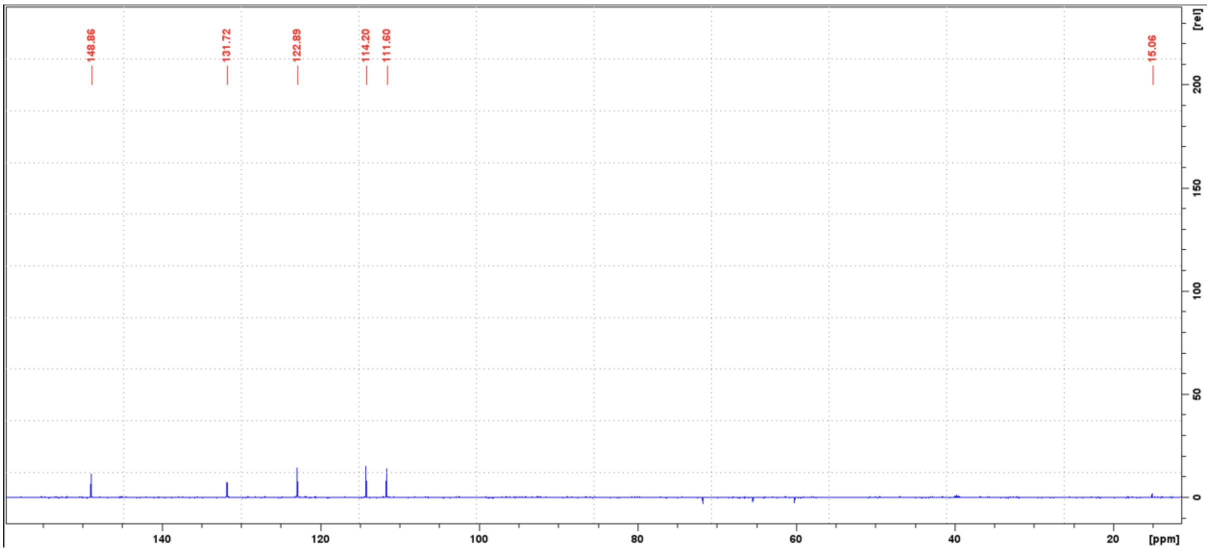

Figure S17. *^{13}C DEPT 135 spectrum of the compound* ***(4a)***

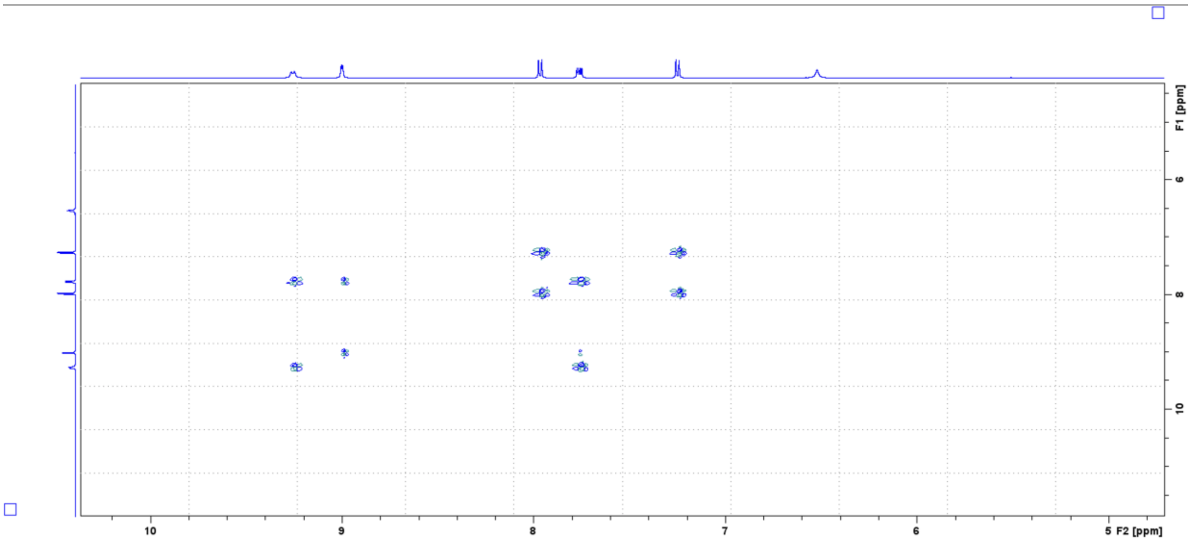

Figure S18. *^{1}H-^{1}H COSY spectrum of the compound* ***(4a)***

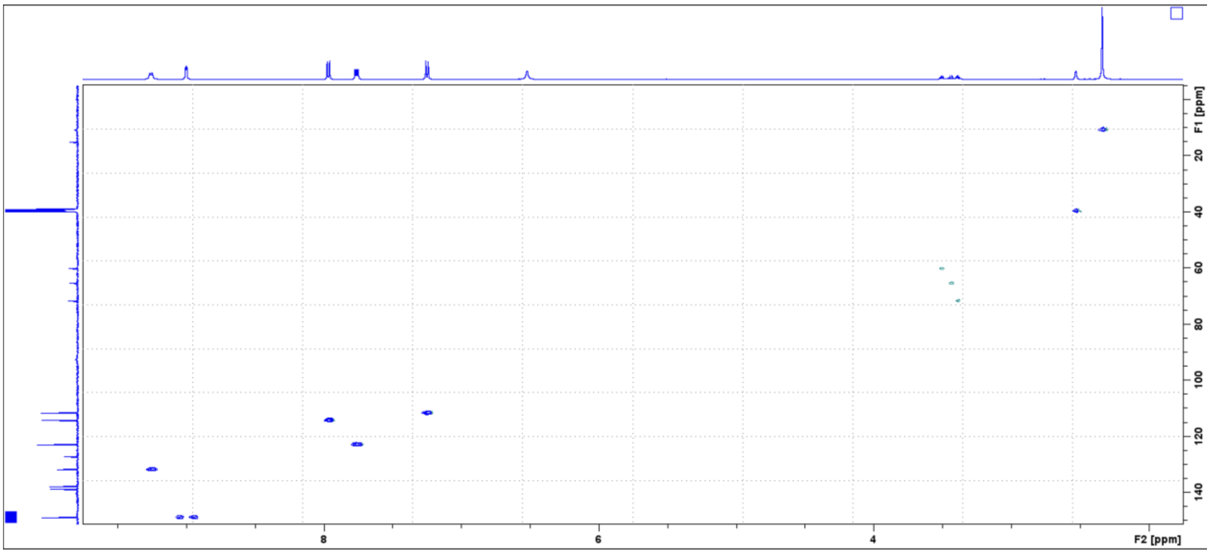

Figure S19. ^{1}H-^{13}C *HSQC spectrum of the compound* ***(4a)***

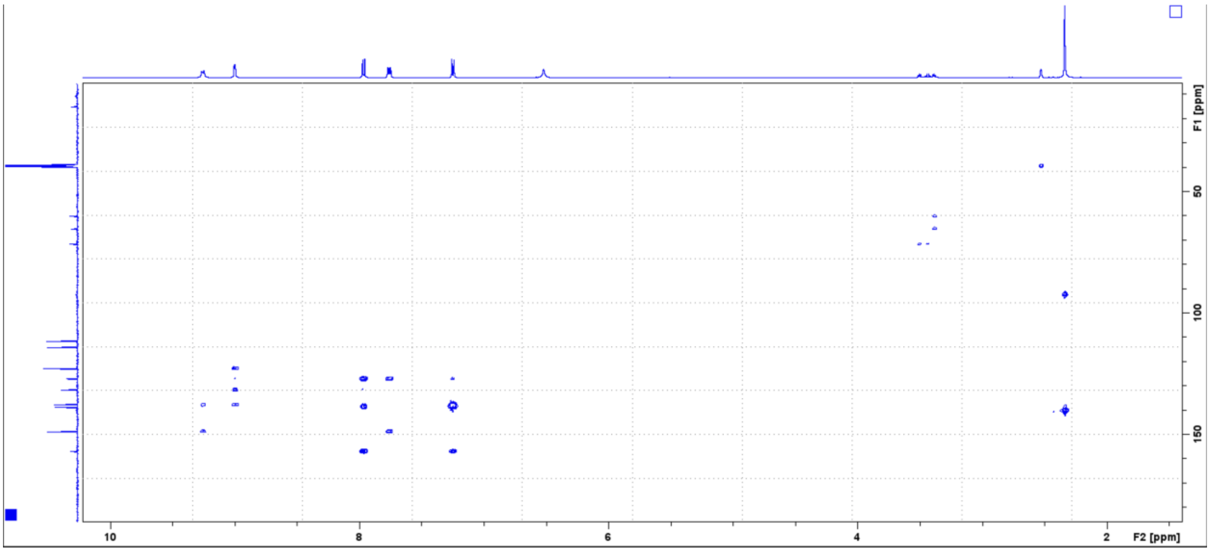

Figure S20. ^{1}H-^{13}C *HMBC spectrum of the compound* ***(4a)***

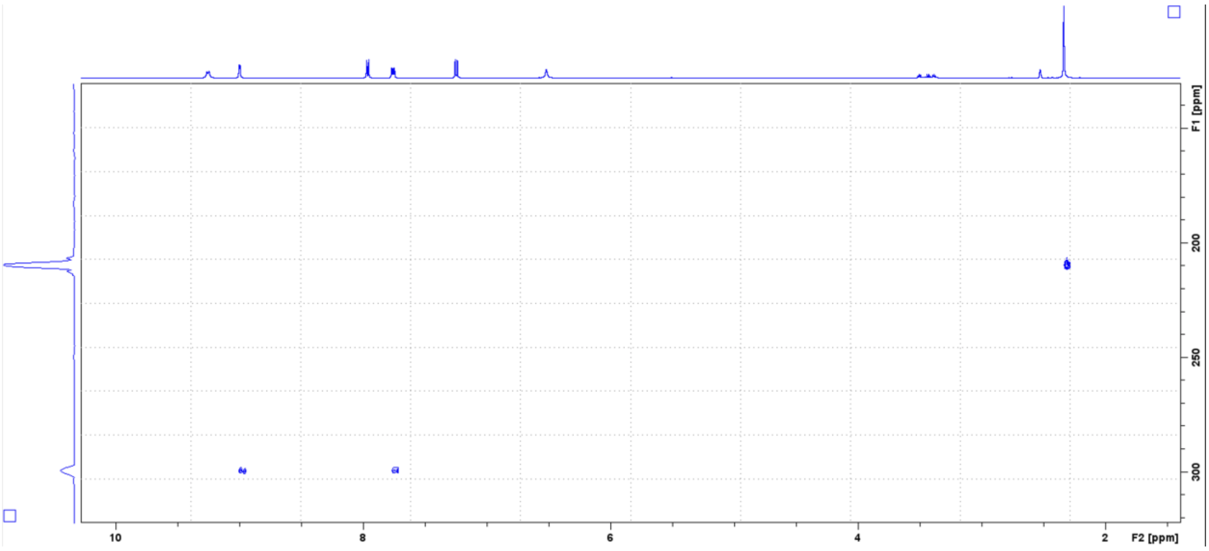

Figure S21. *^{1}H-^{15}N HMBC spectrum of the compound* ***(4a)***

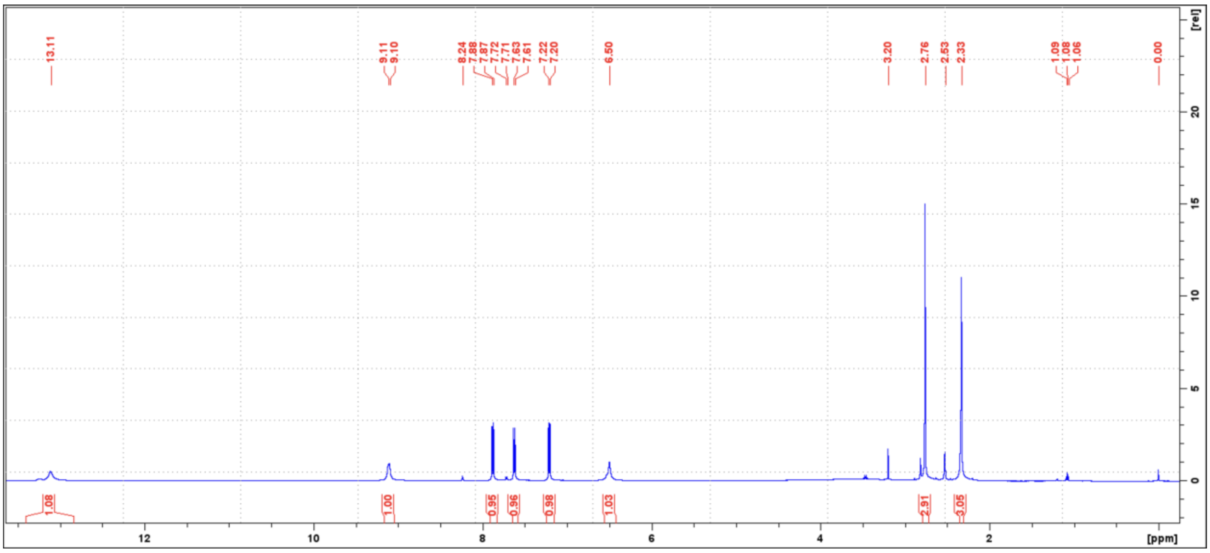

Figure S22. *^{1}H NMR spectrum of the compound* ***(4b)***

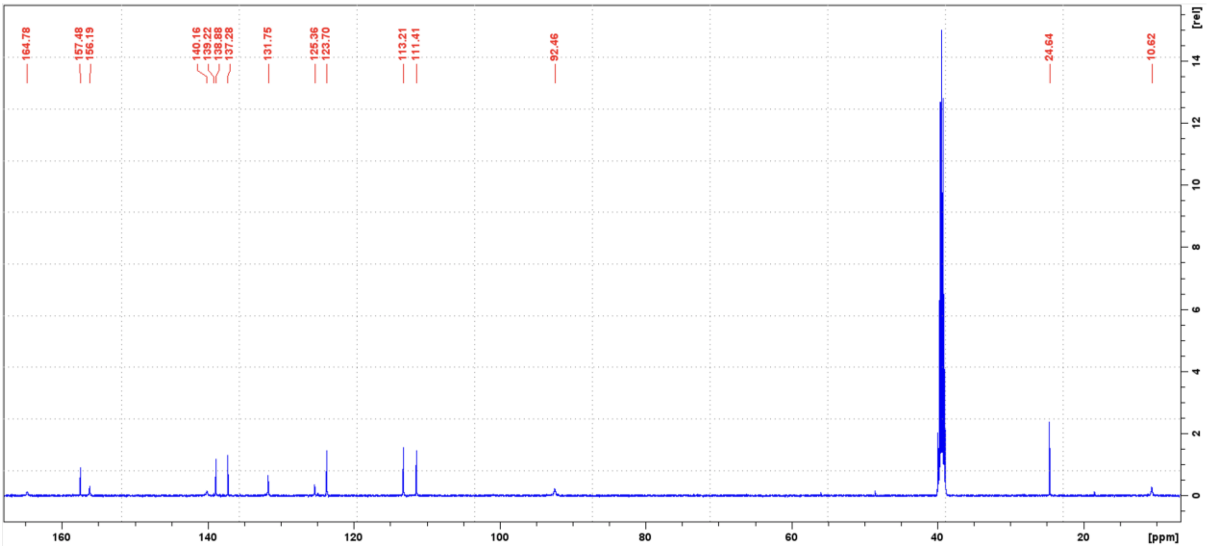

Figure S23. *^{13}C NMR spectrum of the compound* ***(4b)***

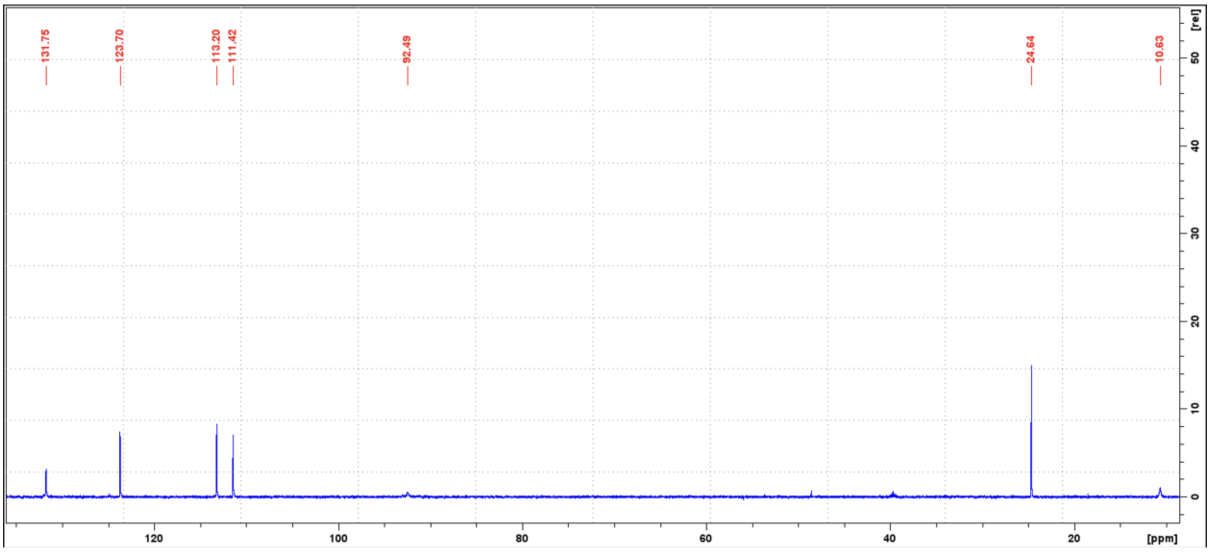

Figure S24. *^{13}C DEPT135 spectrum of the compound* ***(4b)***

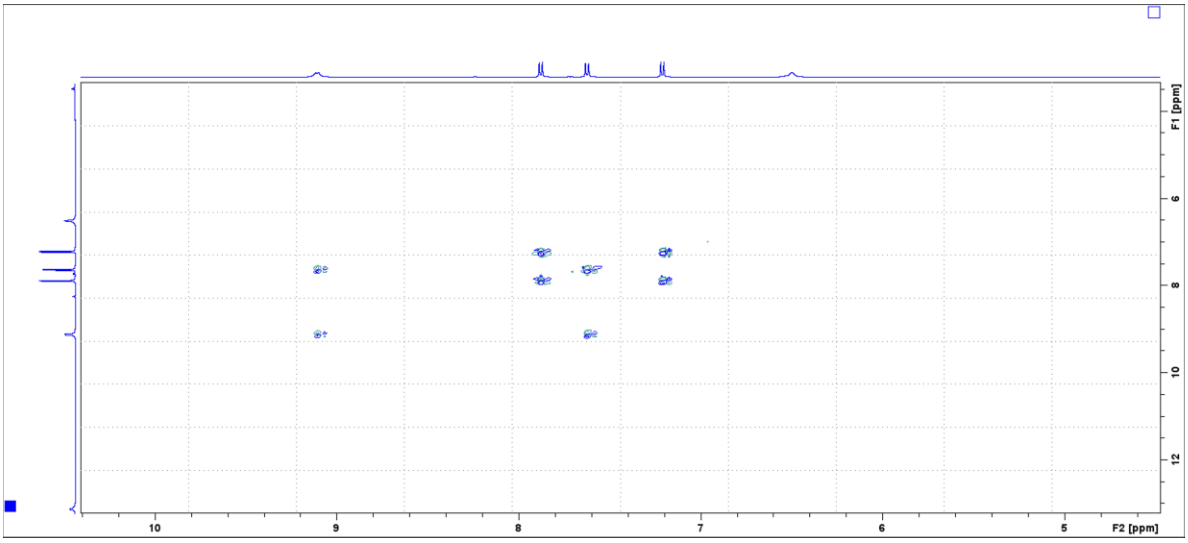

Figure S25. *^{1}H-^{1}H COSY spectrum of the compound* ***(4b)***

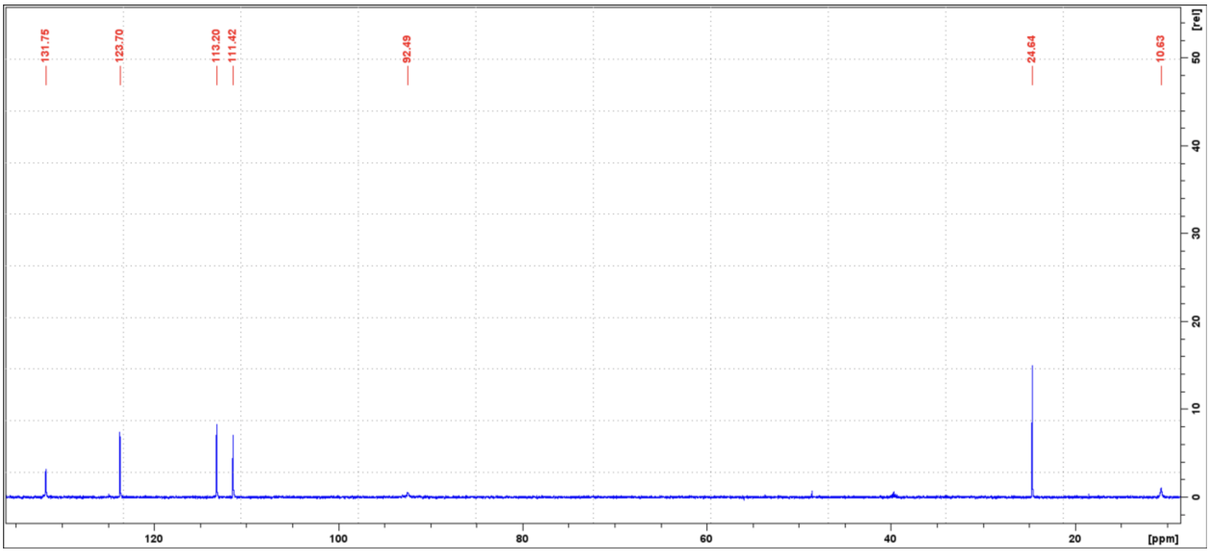

Figure S26. *^{13}C DEPT 135 spectrum of the compound* ***(4b)***

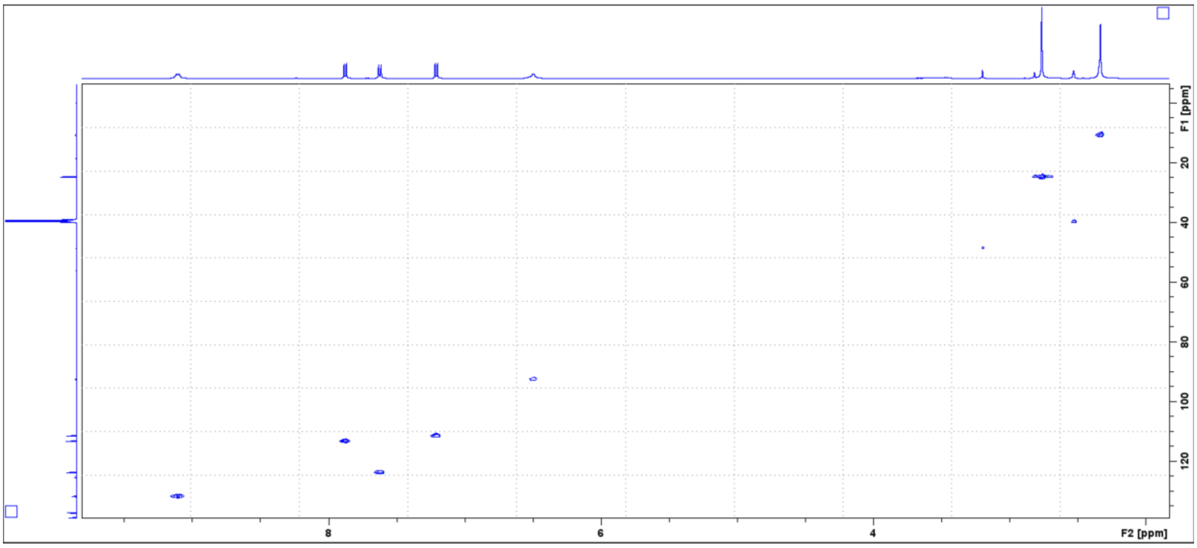

Figure S27 *^{1}H-^{13}C HSQC spectrum of the compound* ***(4b)***

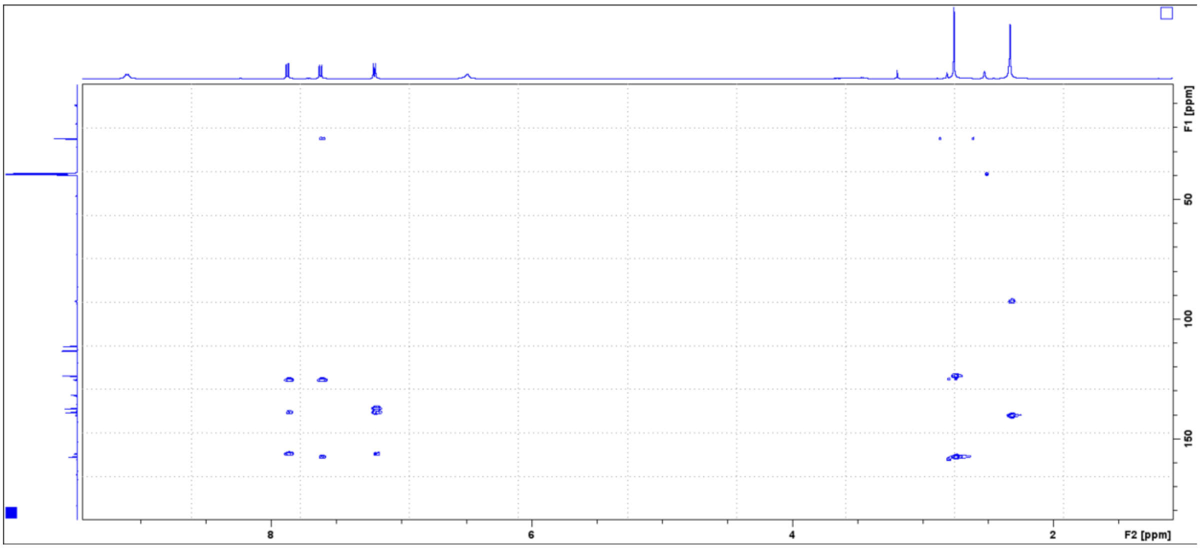

Figure S28. *^{1}H-^{13}C HMBC spectrum of the compound* ***(4b)***

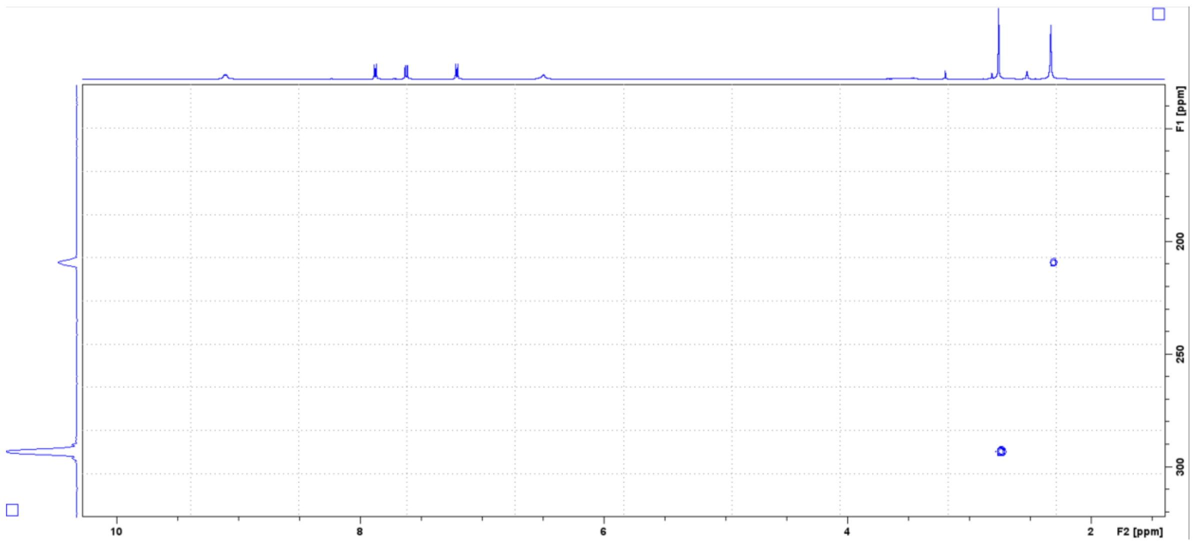

Figure S29. *^{1}H-^{15}N HMBC spectrum of the compound* ***(4b)***

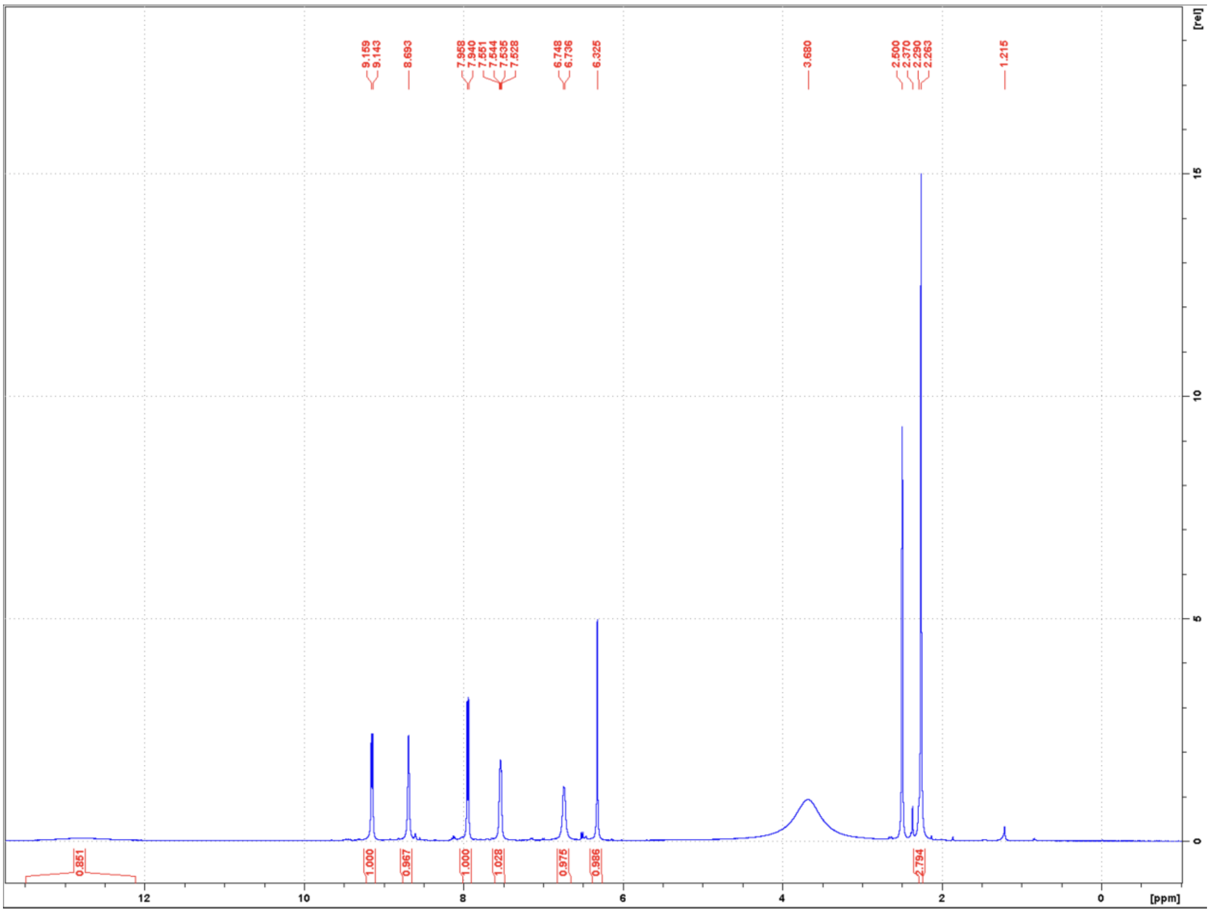

Figure S30. *^{1}H NMR spectrum of the compound* ***(5a)***

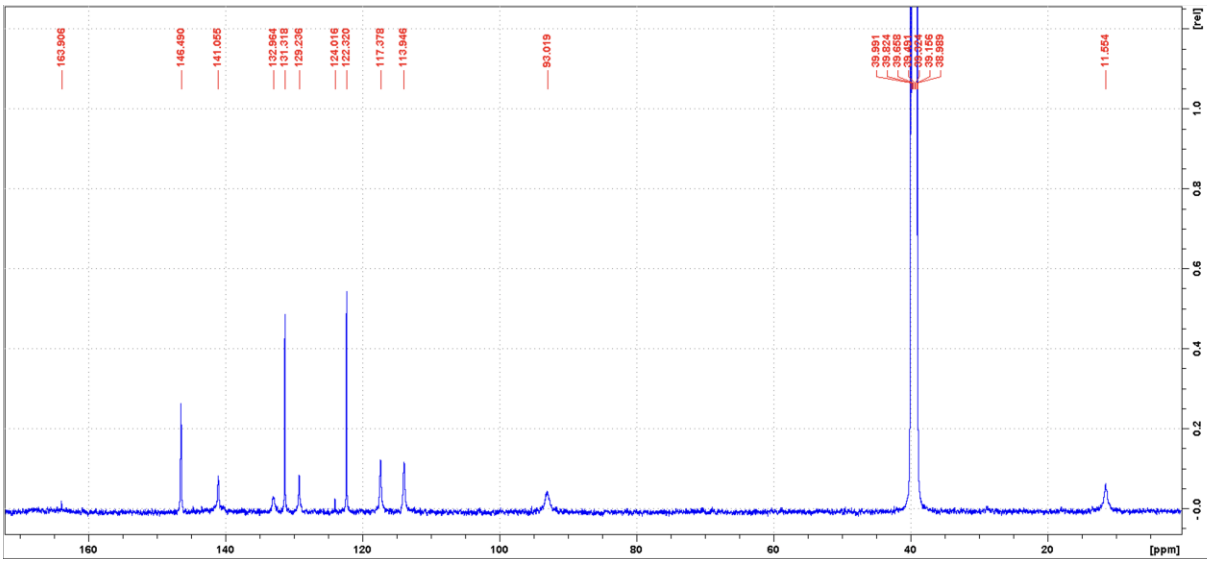

Figure S31. *¹³C NMR spectrum of the compound* ***(5a)***

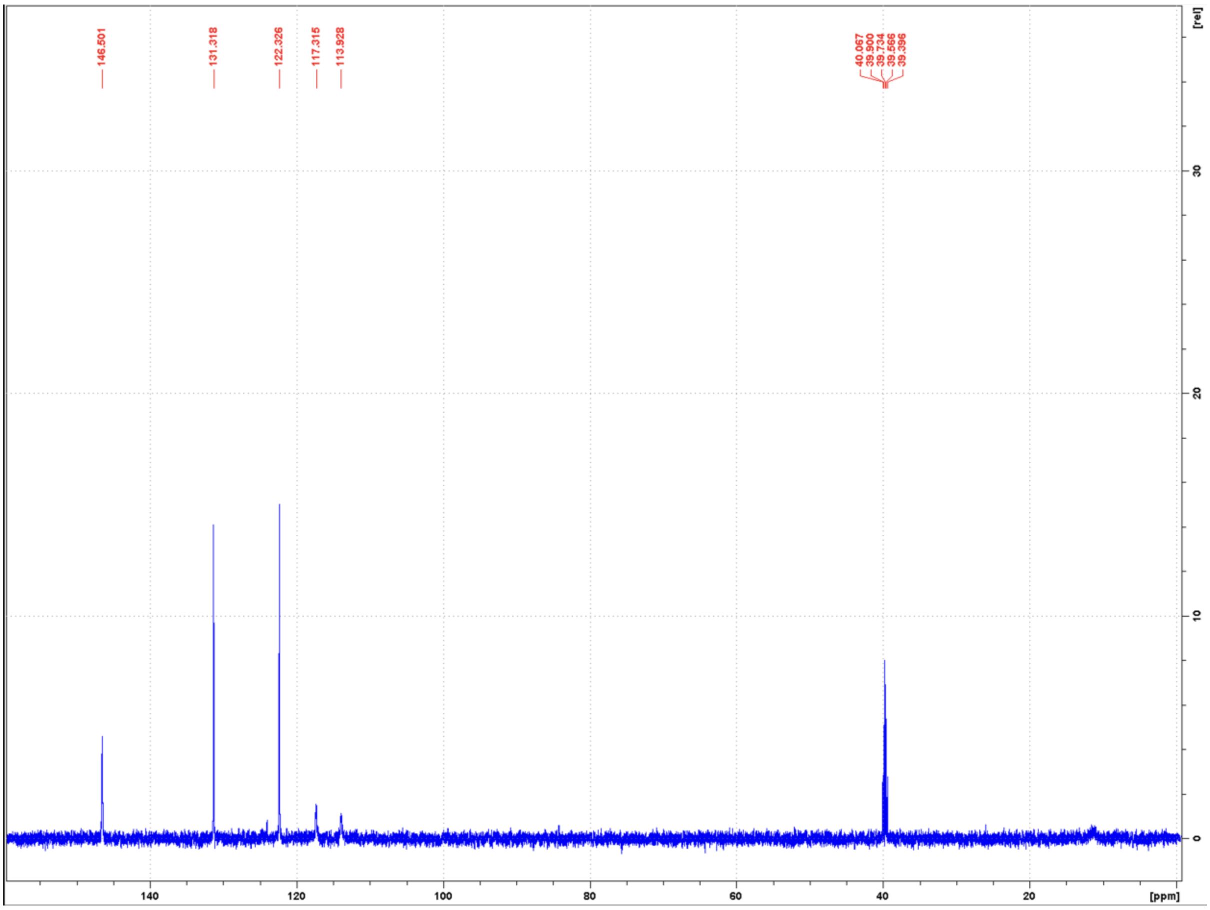

Figure S32. *¹³C DEPT135 spectrum of the compound* ***(5a)***

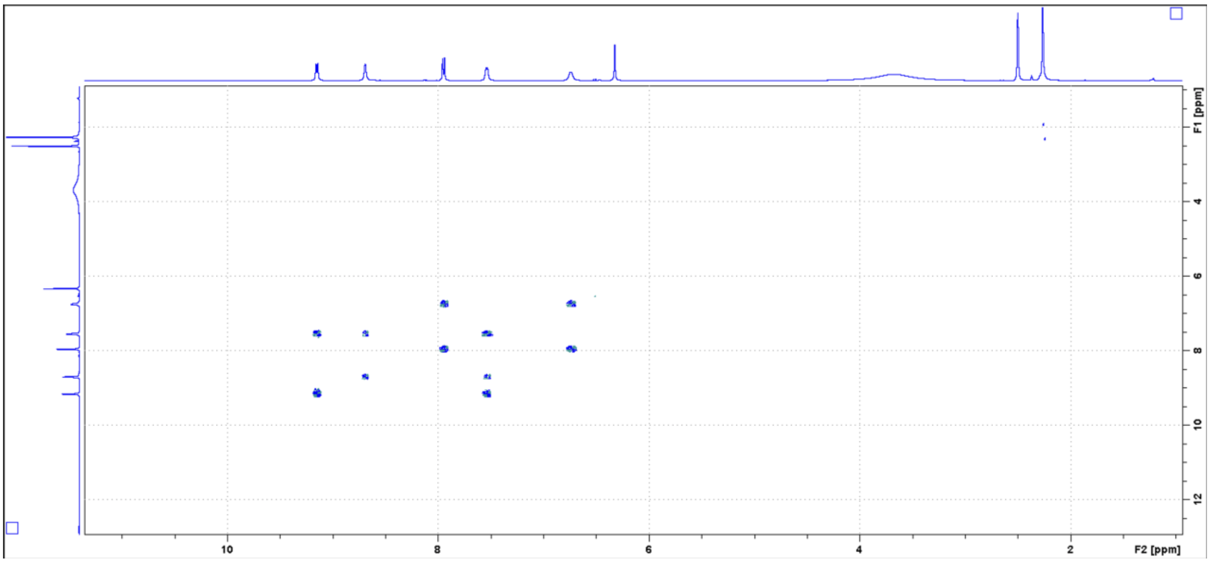

Figure S33. *^{1}H-^{1}H COSY spectrum of the compound* ***(5a)***

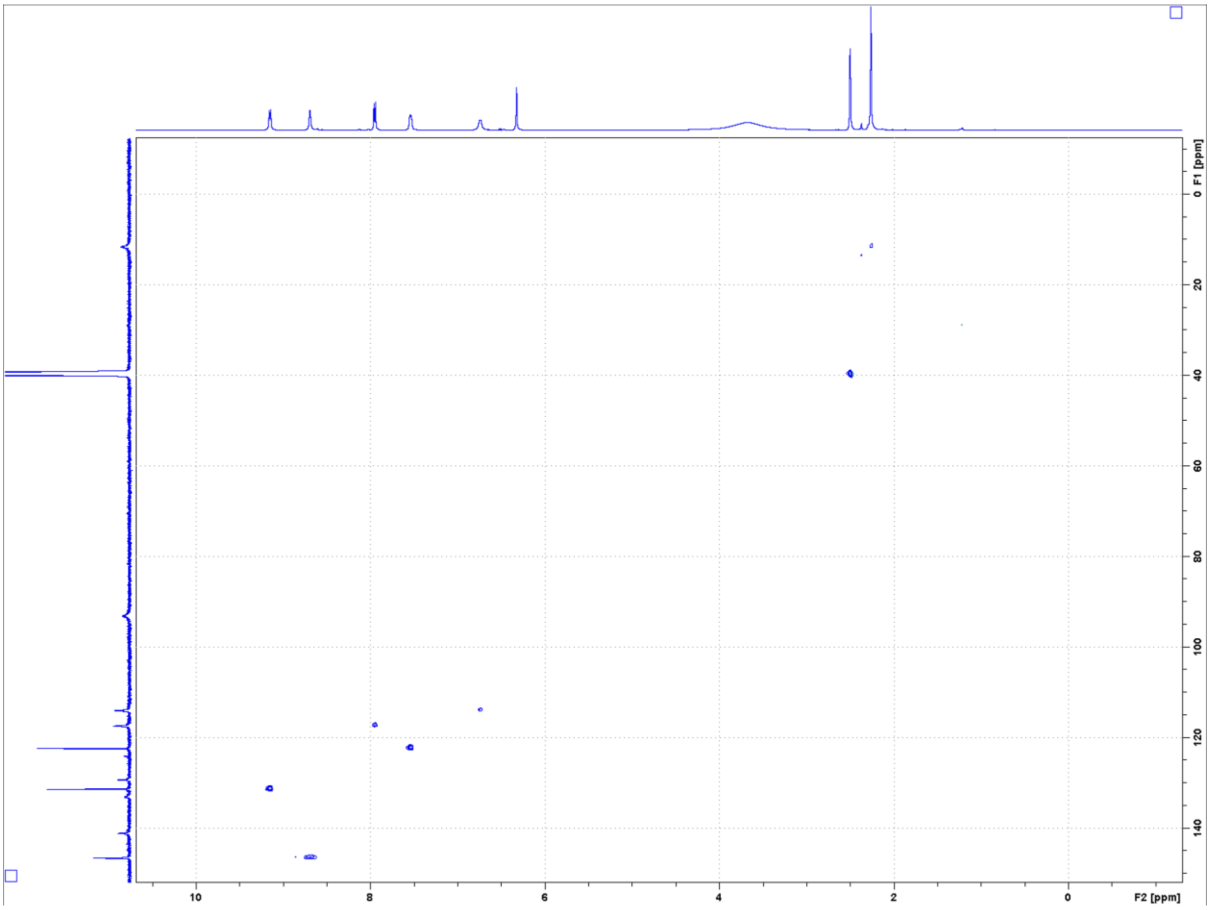

Figure S34. *^{1}H-^{13}C HSQC spectrum of the compound* ***(5a)***

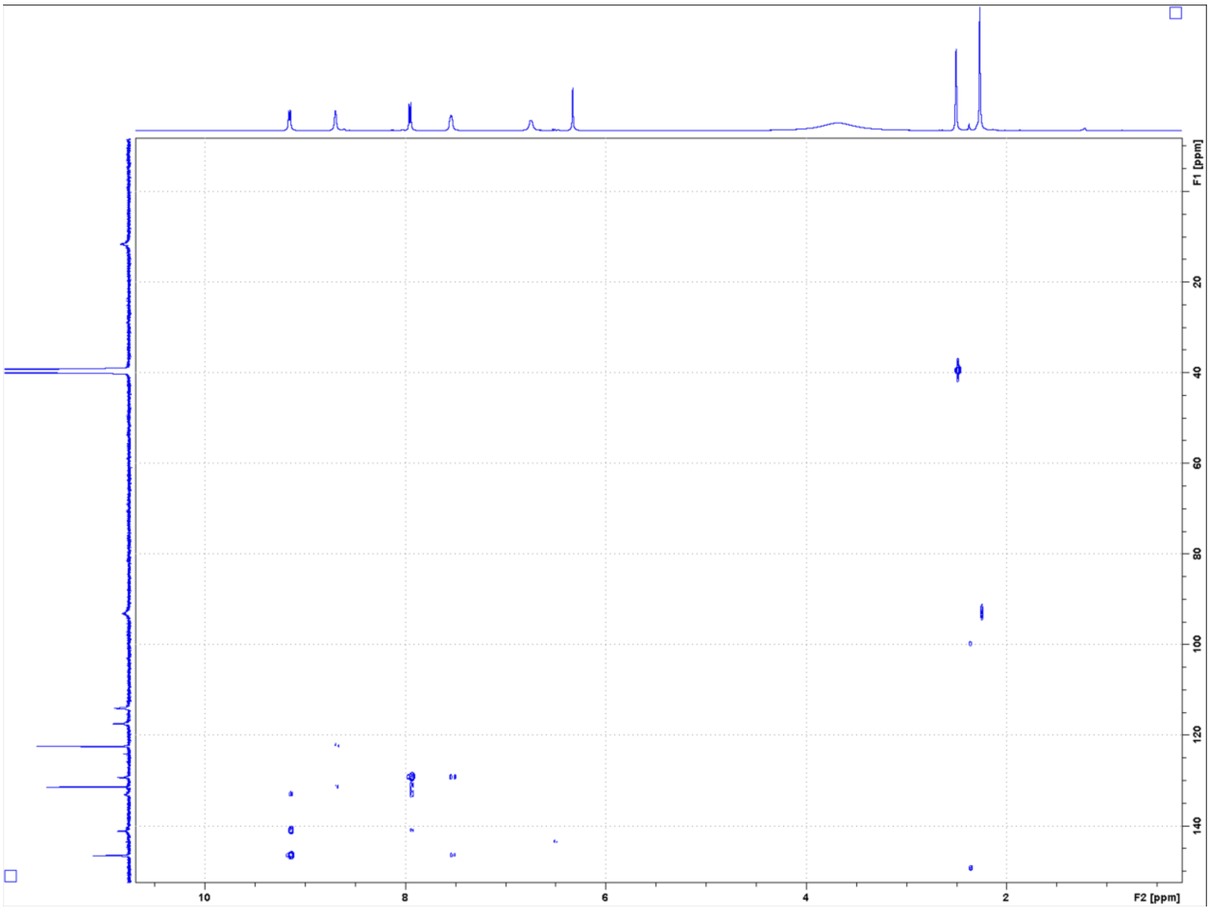

Figure S35. *^1H-^{13}C HMBC spectrum of the compound* ***(5a)***

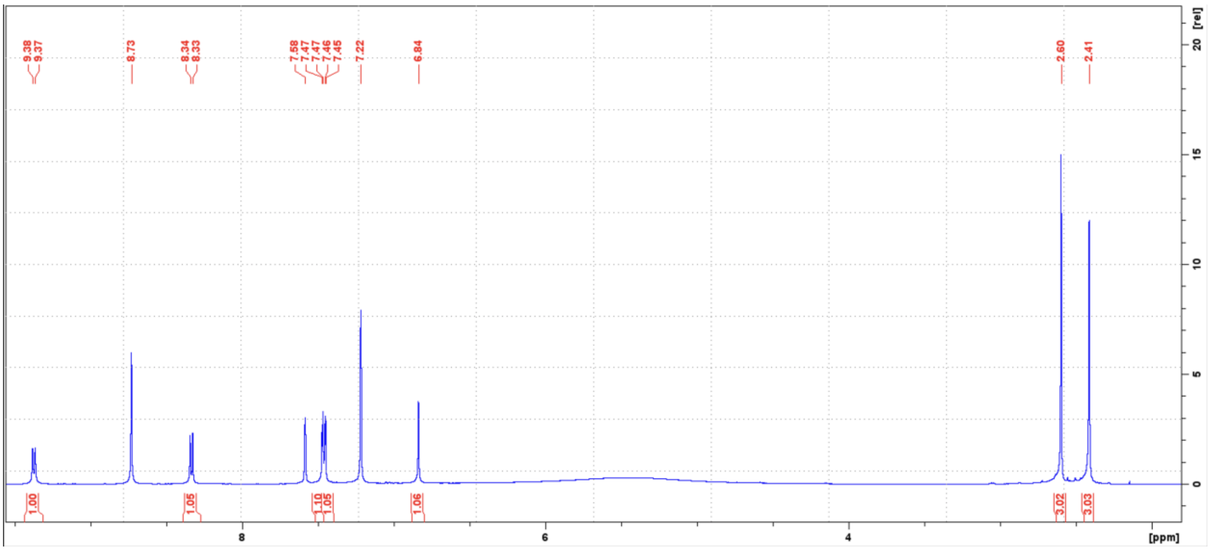

Figure S36. *^{1}H NMR spectrum of the compound* ***(5b)***

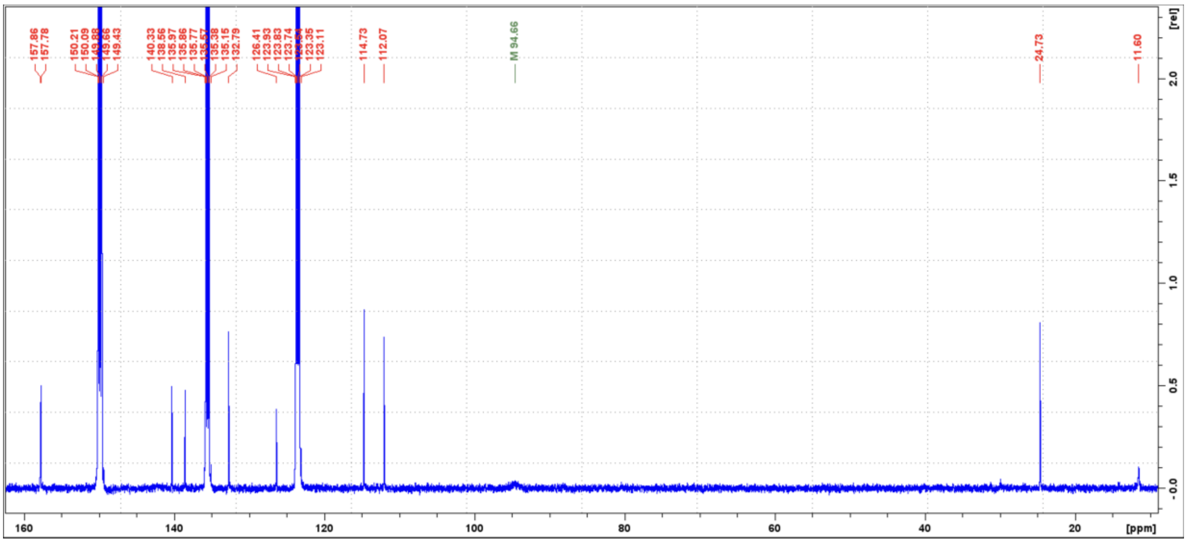

Figure S37. *^{13}C NMR spectrum of the compound* ***(5b)***

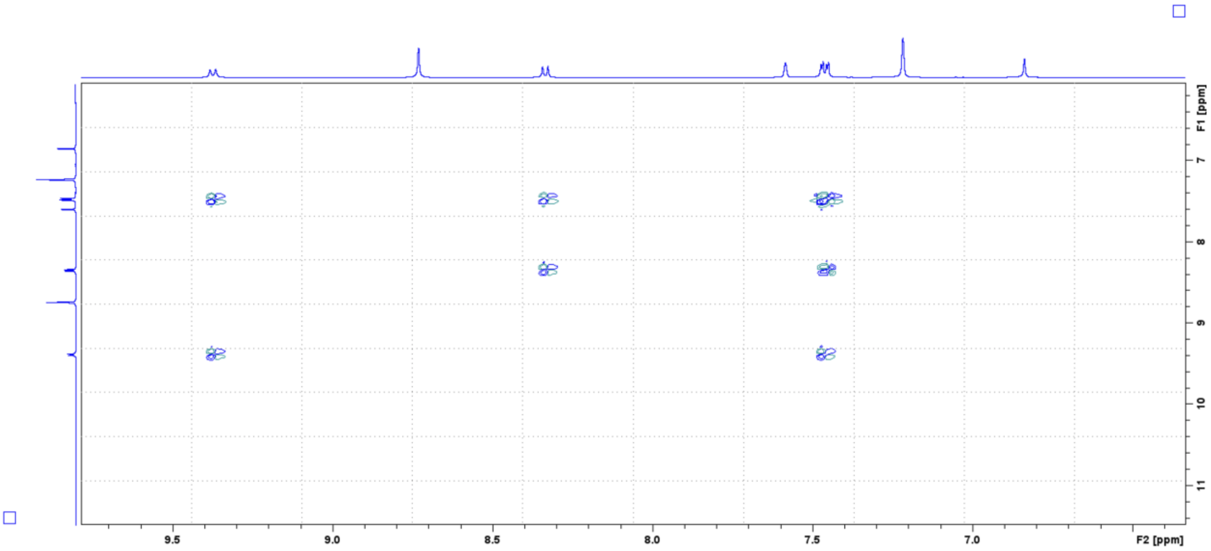

Figure S38. *^{1}H-^{1}H COSY spectrum of the compound* ***(5b)***

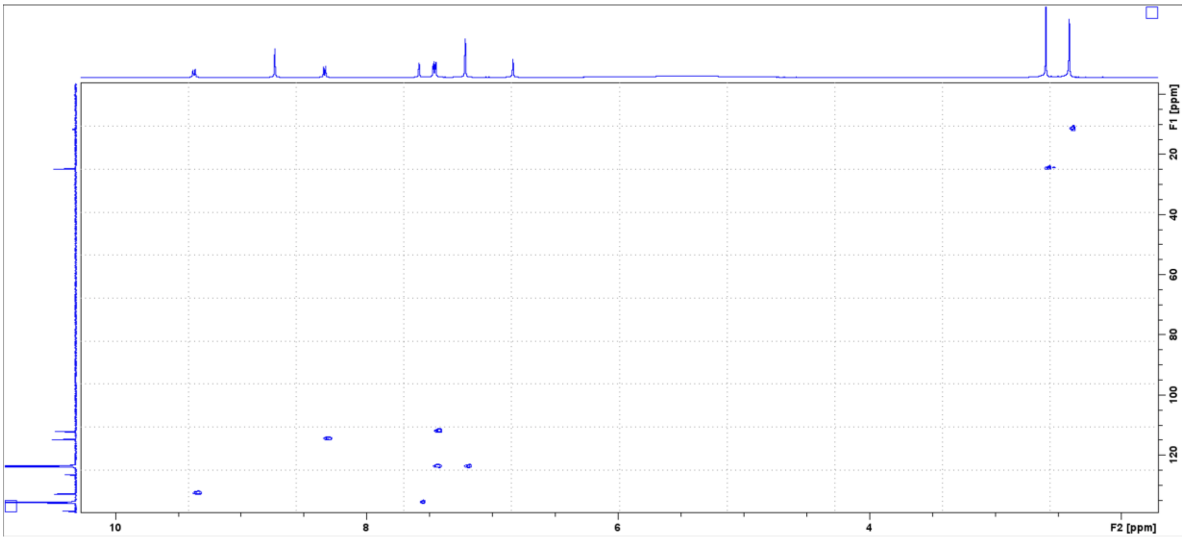

Figure S39. *¹H-¹³C HSQC spectrum of the compound* ***(5b)***

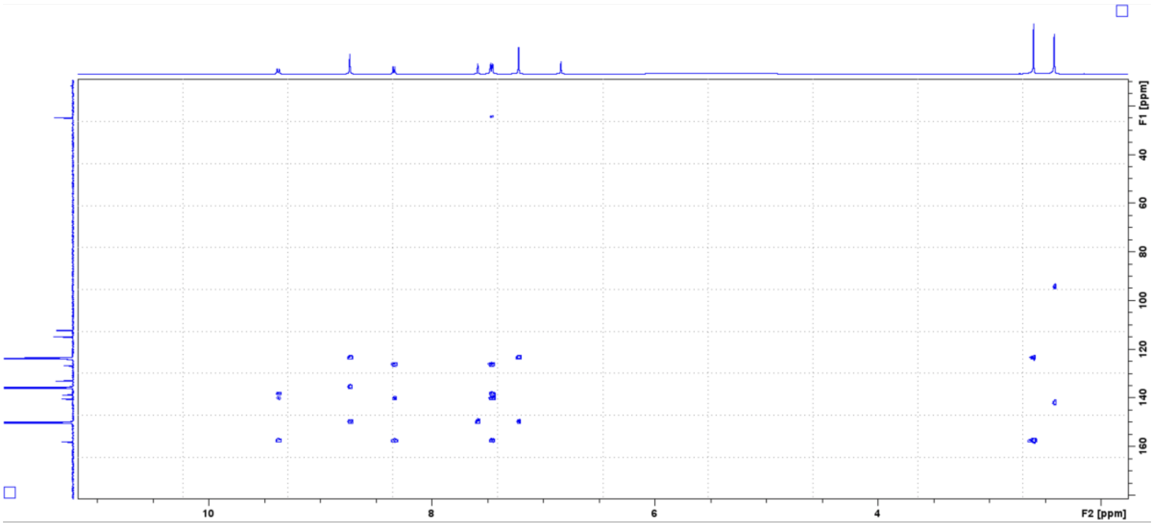

Figure S40. *¹H-¹³C HMBC spectrum of the compound* ***(5b)***

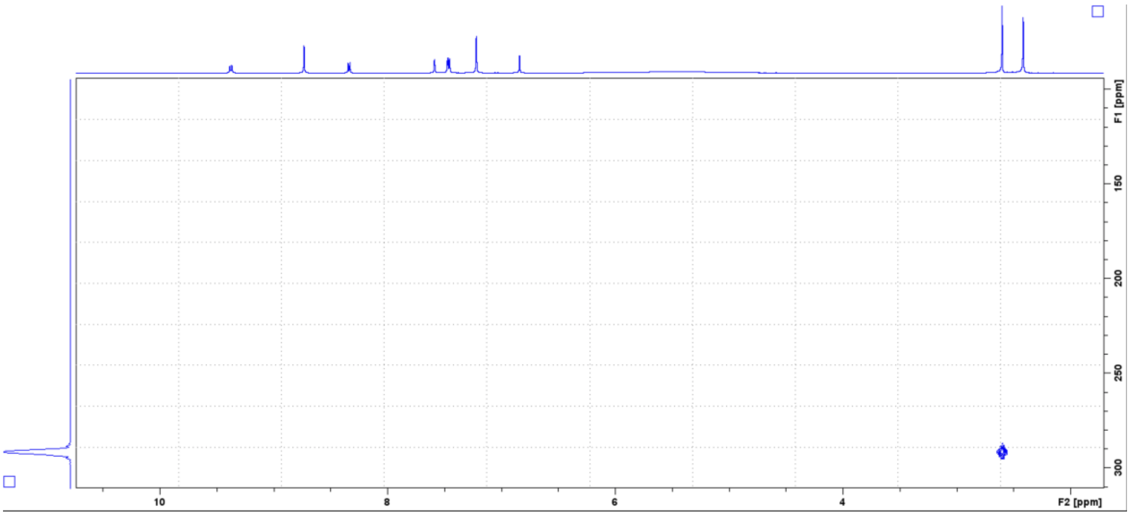

Figure S41. *¹H-¹⁵N HMBC spectrum of the compound* ***(5b)***

IR spectra for compounds (3a), (3b), (4a), (4b), (5a), (5b)

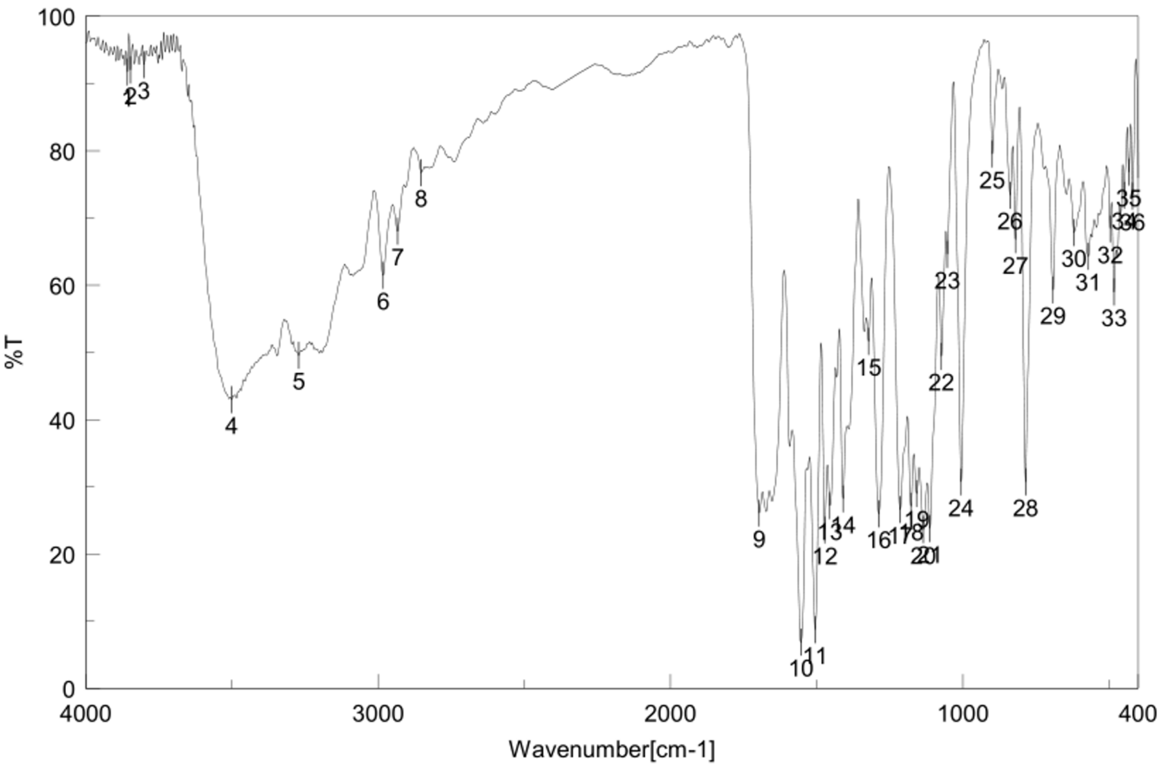

1: 3857.9, 91.722	2: 3847.29, 92.0184	3: 3801.97, 92.7914
4: 3501.13, 42.9901	5: 3271.64, 49.5887	6: 2982.37, 61.4759
7: 2933.2, 68.0277	8: 2853.17, 76.7383	9: 1697.05, 26.1179
10: 1553.38, 6.89192	11: 1505.17, 8.76215	12: 1472.38, 23.6004
13: 1455.03, 27.2191	14: 1409.71, 28.2242	15: 1322.93, 51.6323
16: 1287.25, 25.9858	17: 1213.97, 26.604	18: 1177.33, 27.1107
19: 1158.04, 29.028	20: 1134.9, 23.6217	21: 1112.73, 23.8185
22: 1073.19, 49.3924	23: 1052.94, 64.5993	24: 1005.7, 30.7203
25: 898.666, 79.534	26: 837.919, 73.3828	27: 819.598, 66.774
28: 784.886, 30.7359	29: 691.355, 59.2866	30: 619.038, 67.845
31: 569.862, 64.2952	32: 495.616, 68.3892	33: 482.117, 59.0185
34: 447.404, 73.3659	35: 431.977, 76.8088	36: 419.442, 73.2752

Figure S42. *IR spectrum of the compound* ***(3a)***

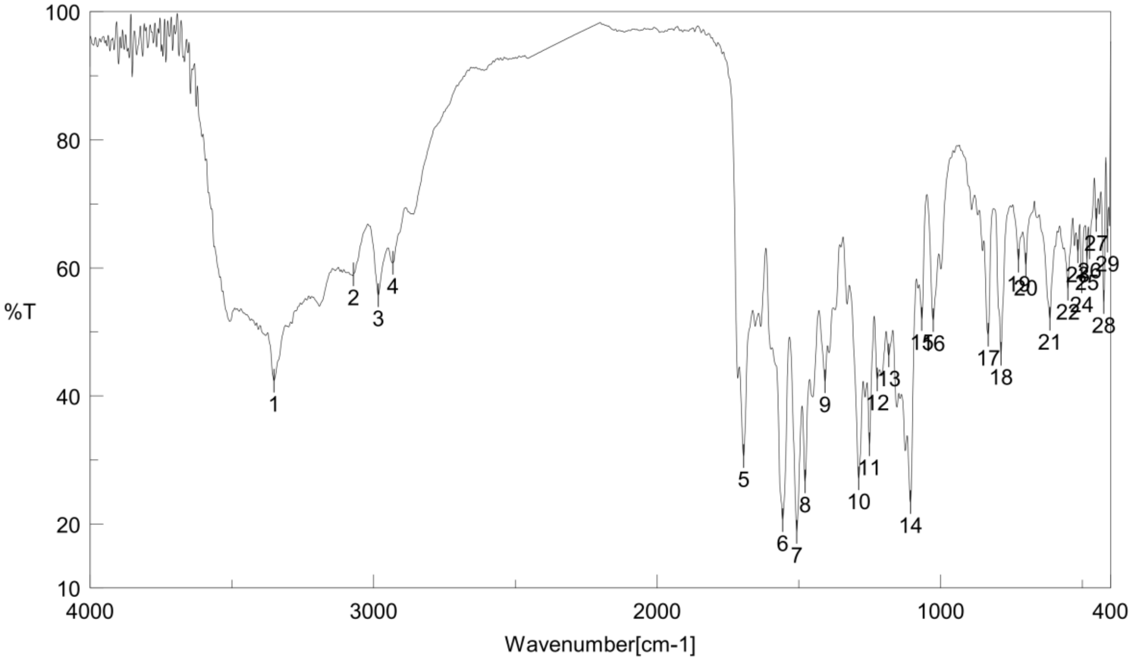

1: 3351.68, 42.3939	2: 3071.08, 58.9972	3: 2983.34, 55.7331
4: 2931.27, 60.8187	5: 1694.16, 30.6361	6: 1557.24, 20.6275
7: 1507.1, 18.783	8: 1477.21, 26.6797	9: 1407.78, 42.3125
10: 1288.22, 27.145	11: 1250.61, 32.4618	12: 1222.65, 42.575
13: 1182.15, 46.3547	14: 1105.98, 23.4424	15: 1066.44, 52.0252
16: 1025.94, 51.8378	17: 832.133, 49.5755	18: 786.815, 46.6062
19: 725.104, 61.1255	20: 699.069, 60.5129	21: 614.217, 52.0438
22: 550.577, 56.7596	23: 514.901, 62.5925	24: 501.401, 57.8872
25: 483.081, 61.3679	26: 473.439, 63.2208	27: 450.297, 67.5158
28: 424.263, 54.6872	29: 410.763, 64.2749	

Figure S43. *IR spectrum of the compound* ***(3b)***

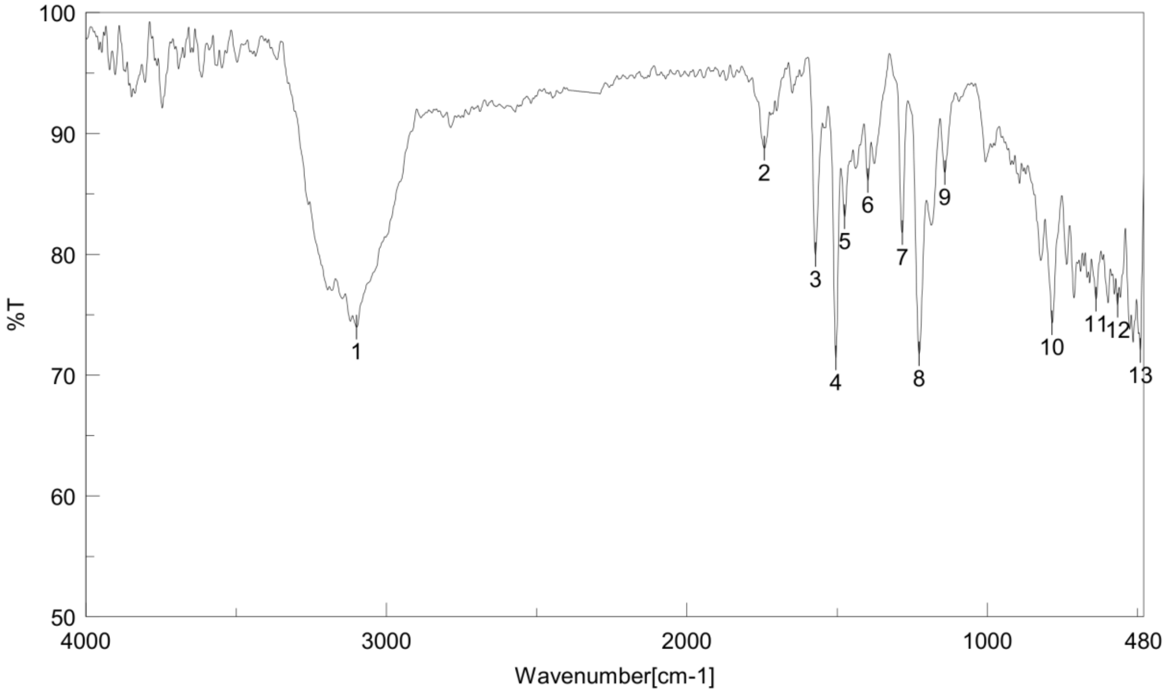

1: 3099.05, 73.9795	2: 1742.37, 88.7889	3: 1571.7, 79.987
4: 1504.2, 71.4487	5: 1475.28, 83.1244	6: 1398.14, 86.1162
7: 1283.39, 81.8045	8: 1227.47, 71.7804	9: 1141.65, 86.7676
10: 784.886, 74.3334	11: 638.323, 76.2824	12: 566.969, 75.8039
13: 490.795, 72.062	14: 451.261, 69.2934	15: 427.155, 50.2432

Figure S44. *IR spectrum of the compound* ***(4a)***

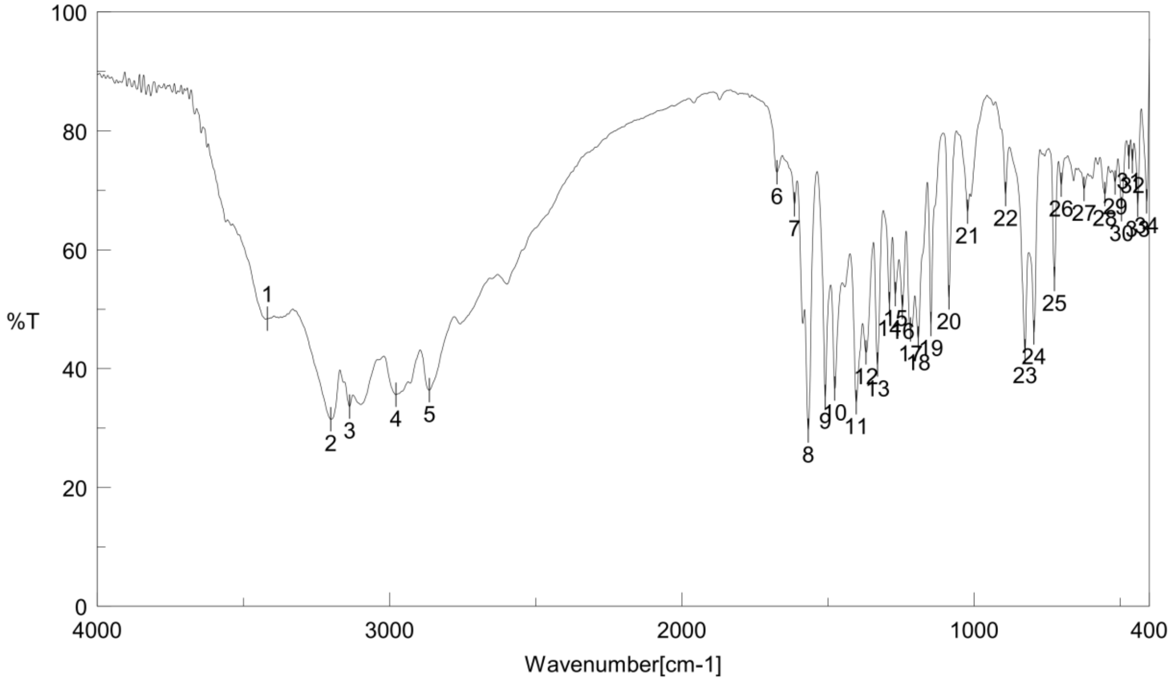

1: 3418.21, 48.4292	2: 3201.26, 31.4804	3: 3136.65, 33.6317
4: 2977.55, 35.6098	5: 2863.77, 36.4059	6: 1673.91, 73.0614
7: 1614.13, 67.6561	8: 1566.88, 29.5909	9: 1509.03, 35.2555
10: 1476.24, 36.653	11: 1402.96, 34.3361	12: 1369.21, 42.7393
13: 1329.68, 40.6799	14: 1289.18, 50.8477	15: 1268.93, 52.5552
16: 1244.83, 50.3442	17: 1217.83, 46.618	18: 1190.83, 45.1071
19: 1147.44, 47.5022	20: 1085.73, 52.0148	21: 1022.09, 66.4227
22: 891.916, 69.3775	23: 825.384, 42.9118	24: 794.528, 46.115
25: 725.104, 55.0862	26: 701.962, 70.9806	27: 622.895, 70.2221
28: 552.506, 69.384	29: 516.829, 71.3235	30: 494.652, 66.8523
31: 470.546, 75.6263	32: 458.011, 74.9257	33: 439.69, 67.5809
34: 408.835, 68.1522		

Figure S45. *IR spectrum of the compound* ***(4b)***

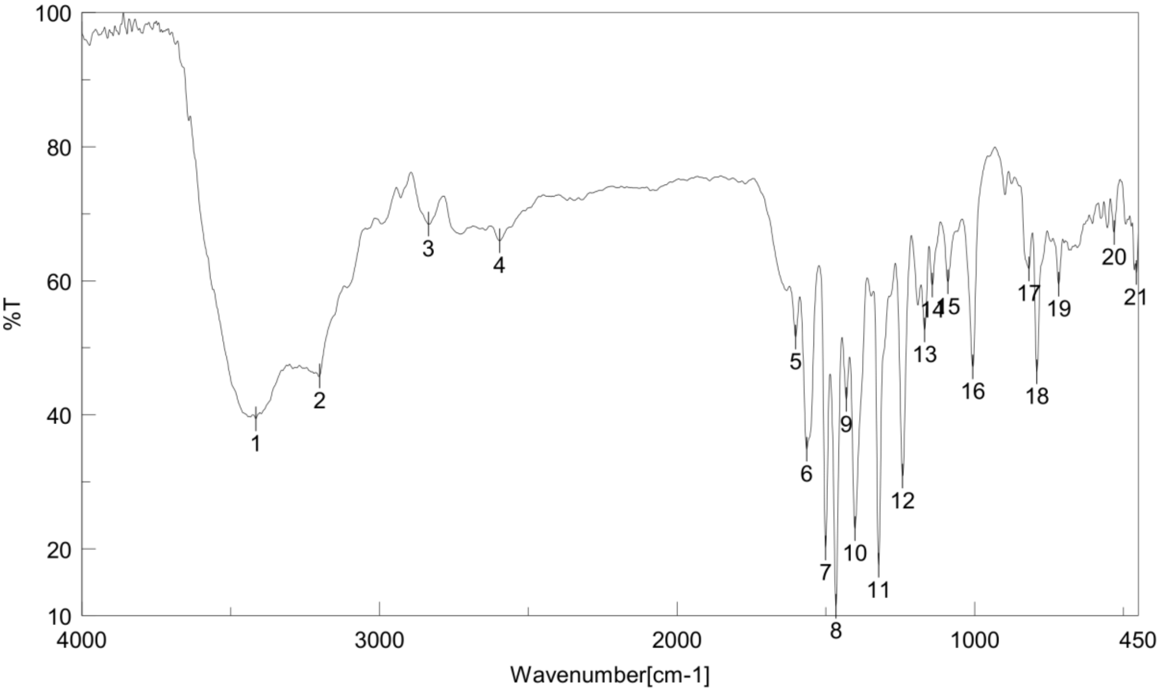

1: 3415.31, 39.3974	2: 3201.26, 45.8214	3: 2835.81, 68.4679
4: 2596.68, 65.9889	5: 1601.59, 51.617	6: 1563.99, 34.858
7: 1500.35, 20.1408	8: 1465.63, 11.4156	9: 1430.92, 42.3149
10: 1402, 23.0131	11: 1321.96, 17.5563	12: 1241.93, 30.887
13: 1167.69, 52.6757	14: 1142.62, 59.3783	15: 1089.58, 59.8646
16: 1005.7, 47.1582	17: 817.67, 61.7921	18: 790.671, 46.4292
19: 717.39, 59.4829	20: 531.293, 67.2295	21: 456.082, 61.2441

Figure S46. *IR spectrum of the compound* ***(5a)***

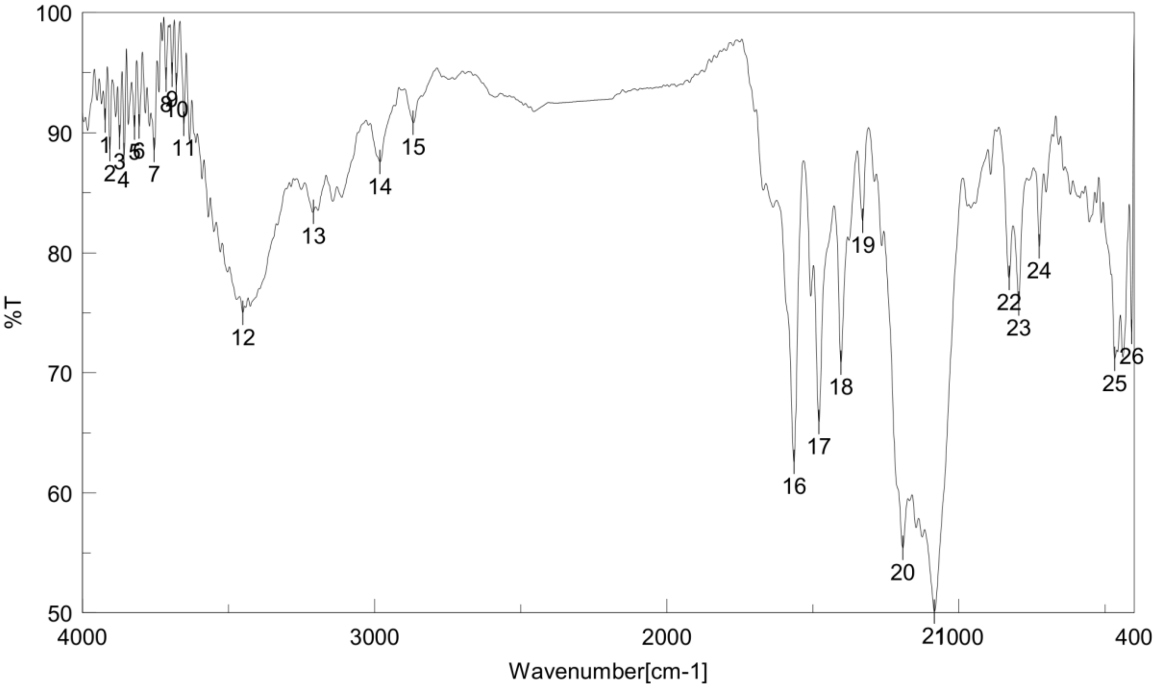

1: 3922.5, 91.0275	2: 3906.11, 88.6319	3: 3873.33, 89.6219
4: 3857.9, 88.1398	5: 3822.22, 90.4332	6: 3805.83, 90.512
7: 3754.73, 88.5664	8: 3714.23, 94.4115	9: 3693.01, 94.8205
10: 3679.51, 93.9758	11: 3653.48, 90.7471	12: 3450.99, 75.0104
13: 3209.93, 83.4105	14: 2982.37, 87.5655	15: 2868.59, 90.8453
16: 1563.99, 62.5884	17: 1479.13, 65.8927	18: 1403.92, 70.8595
19: 1329.68, 82.6706	20: 1192.76, 55.4238	21: 1083.8, 50.1227
22: 828.277, 77.8987	23: 795.493, 75.7738	24: 725.104, 80.5372
25: 466.689, 71.1668	26: 408.835, 73.423	

Figure S47. *IR spectrum of the compound* ***(5b)***

__Table S1.__ Summary IR data for the synthesized compounds

Compound	IR bands and assignments
3a	3501 (ν_{OH}), 3272 (ν_{NH}), 2982 ($\nu_{Car\text{-}H}$), 1697($\nu_{C=O}$), 1553 ($\nu_{Car.=Car.}$), 1505 (δ_{NH}), 1472, 1455 (δ_{CH})
3b	3352 (ν_{OH}), 3071 ($\nu_{Car\text{-}H}$), 2983 ($\nu_{Car\text{-}H}$), 2931 ($\nu_{C\text{-}H}$), 1694 ($\nu_{C=O}$), 1557 ($\nu_{Car.=Car.}$), 1507 (δ_{NH}), 1477, 1407 (δ_{CH})
4a	3099 ($\nu_{Car\text{-}H}$), 1572 (δ_{NH}), 1504 ($\delta_{C\text{-}NH}$), 1475 (δ_{CH})
4b	3418 (ν_{OH}), 3201 ($\nu_{Car\text{-}H}$), 3137 ($\nu_{Car\text{-}H}$), 2978 (ν_{CH}), 1567 (δ_{NH}), 1476 (δ_{CH})
5a	3415 (ν_{OH}), 3201 (ν_{NH}), 2836 ($\nu_{Car\text{-}H}$), 1600 ($\nu_{Car.=Car.}$), 1536 (δ_{NH}), 1550 ($\nu_{Car.=Car.}$), 1465, 1431 (δ_{CH})
5b	3451 (ν_{OH}), 3210 (ν_{NH}), 2983, 2869 (ν_{CH}), 1564 (δ_{NH}), 1479 ($\nu_{C\text{-}C}$), 1403 (δ_{CH})

UV-Vis spectra for compounds (3a), (3b), (4a), (4b), (5a), (5b)

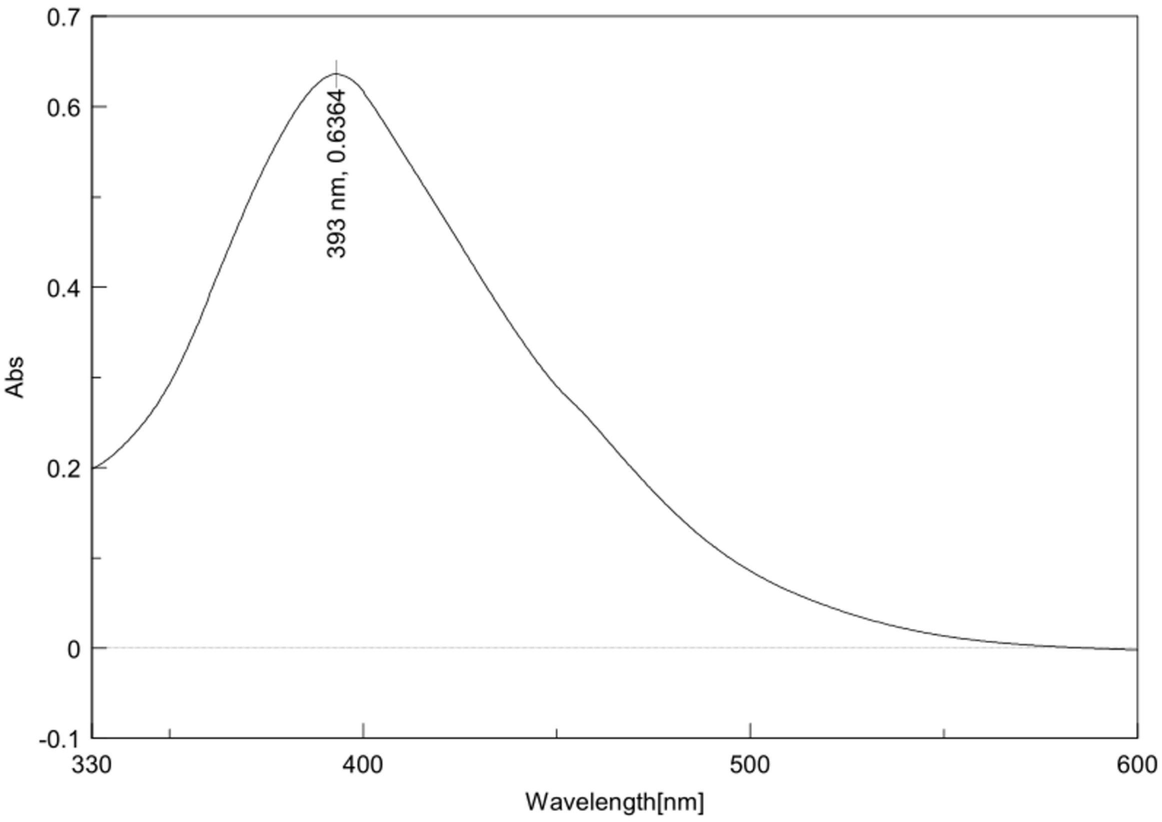

__Figure S48.__ UV-Vis spectrum of the compound __(3a)__

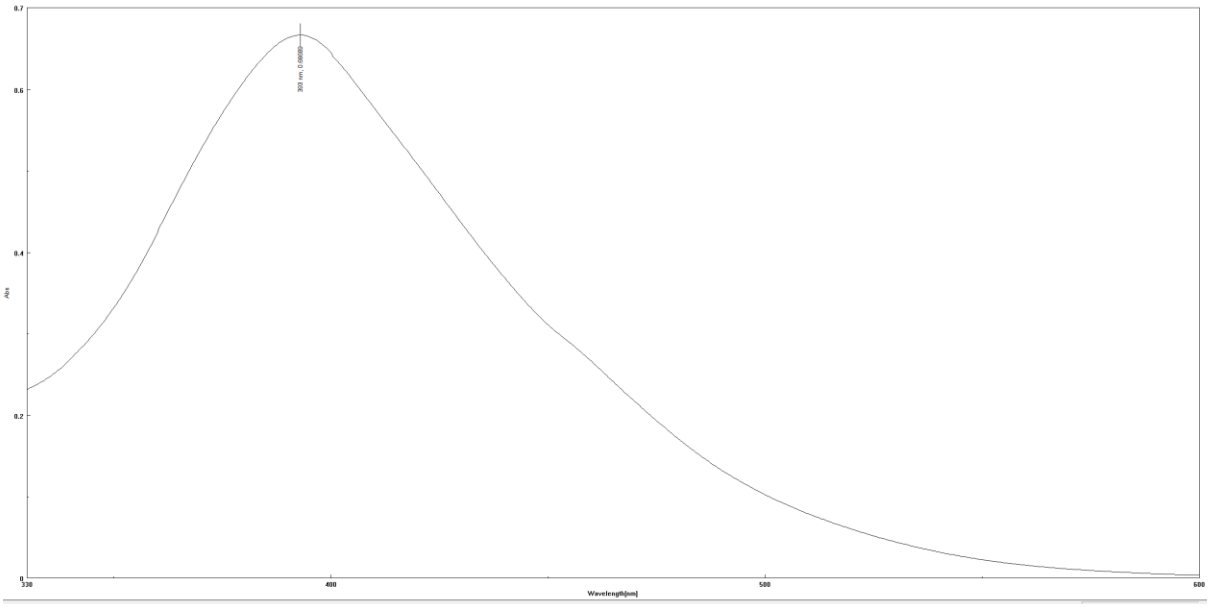

Figure S49. *UV-Vis spectrum of the compound* ***(3b)***

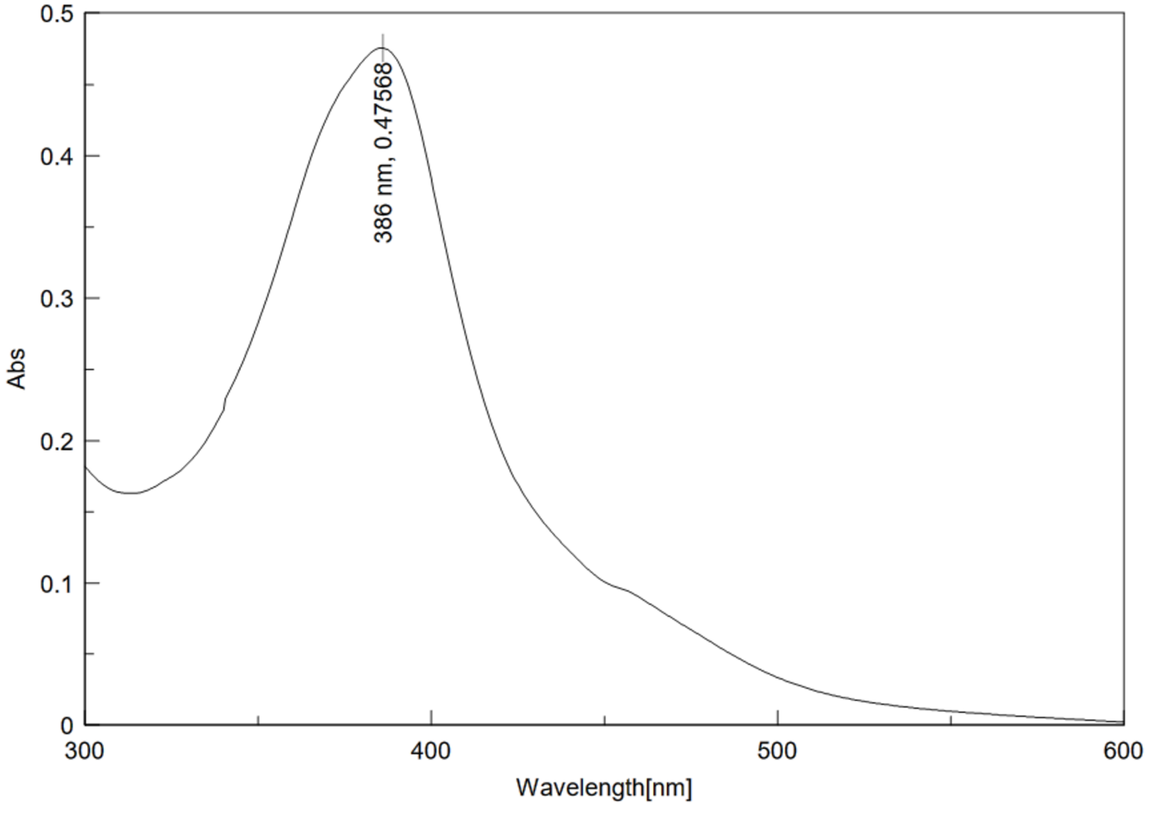

Figure S50. *UV-Vis spectrum of the compound* ***(4a)***

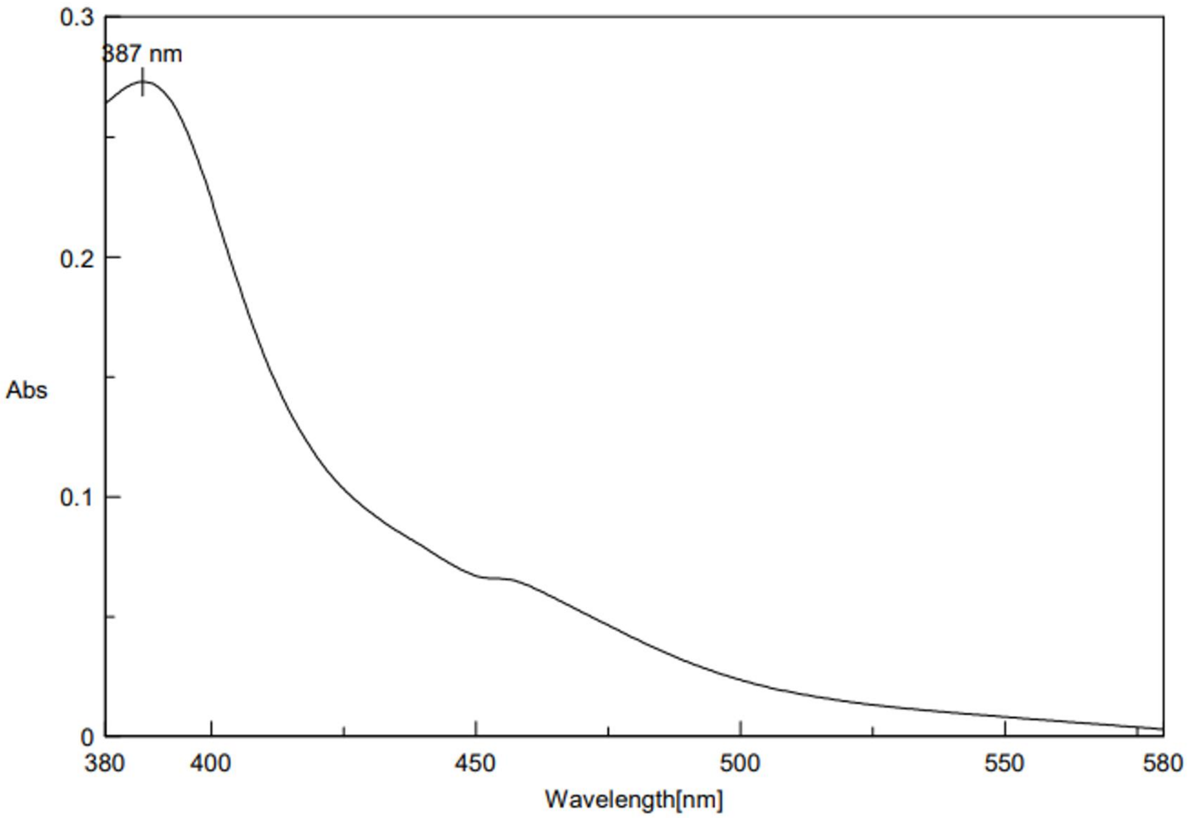

Figure S51. *UV-Vis spectrum of the compound* ***(4b)***

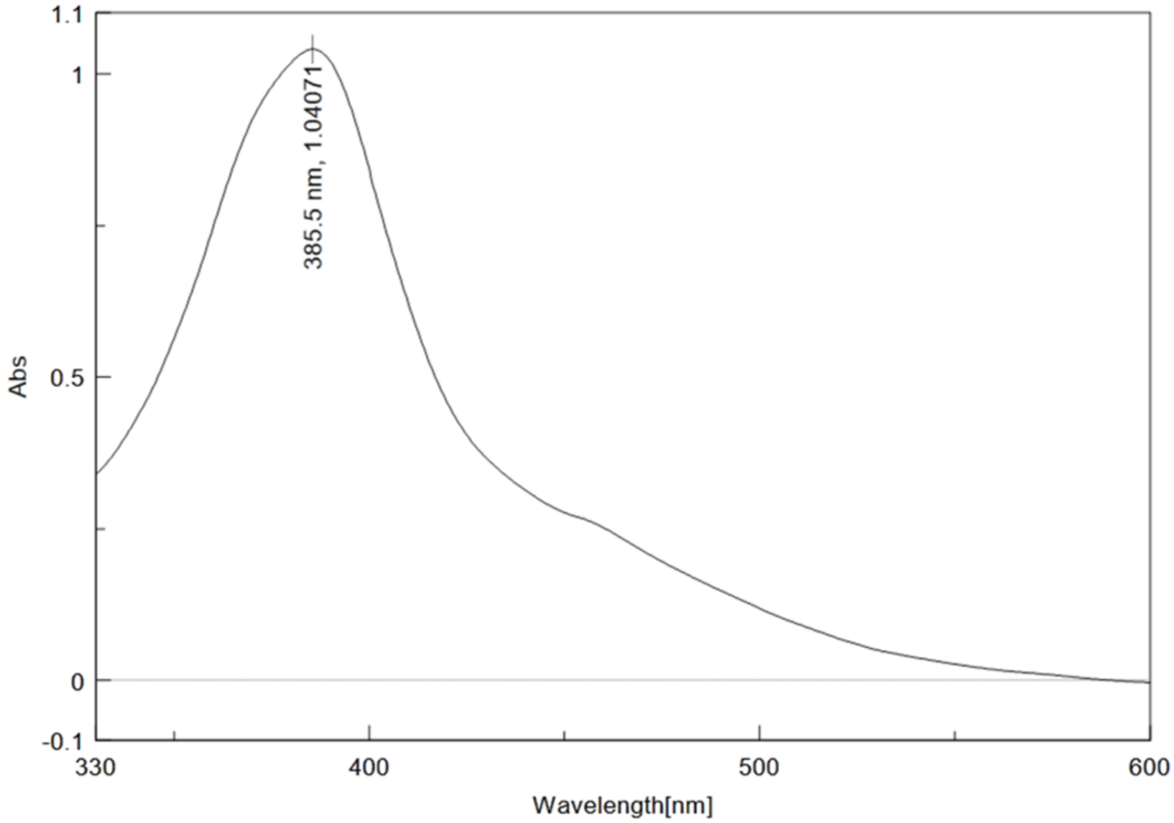

Figure S52. *UV-Vis spectrum of the compound* ***(5a)***

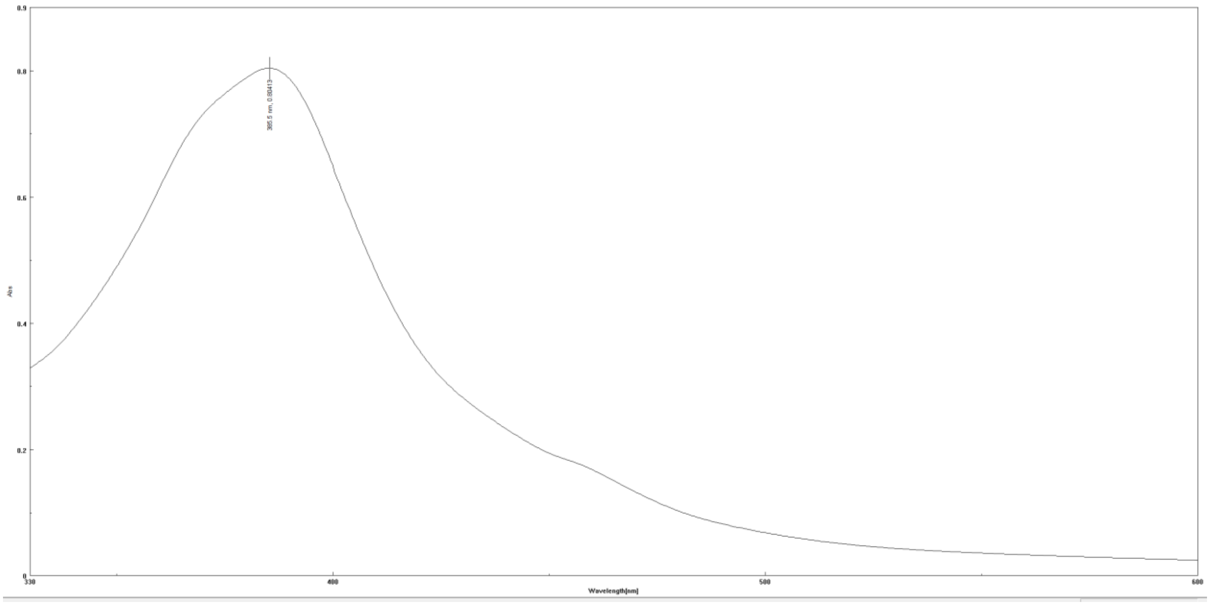

Figure S53. *UV-Vis spectrum of the compound* ***(5b)***

Table S2. *Summary UV-Vis data for the synthesized compounds*

Compound	Molar concentration [M]	λ_{max} [nm]	$\varepsilon_{\lambda max}$ [M^{-1} cm^{-1}]
3a	5.533×10^{-5}	393	11502
3b	4.130×10^{-5}	393	16148
4a	2.299×10^{-5}	386	20691
4b	9.983×10^{-6}	387	30400
5a	6.728×10^{-5}	385.5	15469
5b	$3.240 x 10^{-5}$	385.5	24818

LC-HRMS data for compounds (3a), (3b), (4a), (4b), (5a), (5b)

Compound Details

Cpd. 1: C16 H15 N5 O3

Formula	RT	Mass	Mass (Tgt)	Abund	Algorithm
C16 H15 N5 O3	1.121	325.1172	325.1175	1575030	FBF

Compound Spectra (overlaid)

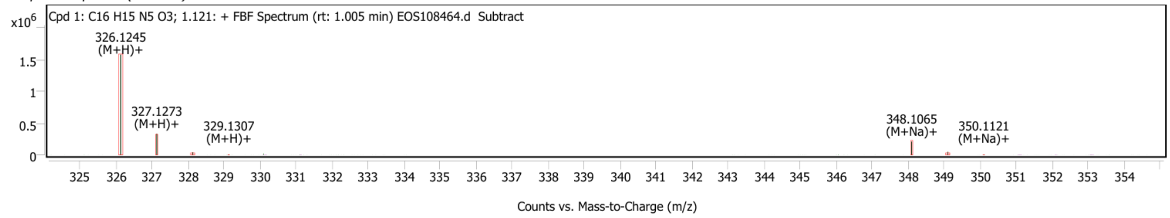

Spectrum Peaks

m/z	m/z (Calc)	Diff (ppm)	Abund	Height %	Height % (Calc)	Ion Species	Z
326.1245	326.1248	-0.96	1575030	100.00	100.00	(M+H)+	1
327.1273	327.1276	-0.89	330103	20.96	19.43	(M+H)+	1
328.1292	328.1300	-2.42	41913	2.66	2.41	(M+H)+	1
329.1307	329.1324	-5.02	4469	0.28	0.22	(M+H)+	1
348.1065	348.1067	-0.56	207850	100.00	100.00	(M+Na)+	1
349.1084	349.1095	-3.21	48202	23.19	19.42	(M+Na)+	1
350.1121	350.1119	0.46	4816	2.32	2.40	(M+Na)+	1

Figure S54. *LC-HRMS data for compound* ***(3a)***

Compound Details

Cpd. 1: C17 H17 N5 O3

Formula	RT	Mass	Mass (Tgt)	Abund	Algorithm
C17 H17 N5 O3	1.190	339.1328	339.1331	1444334	FBF

Compound Spectra (overlaid)

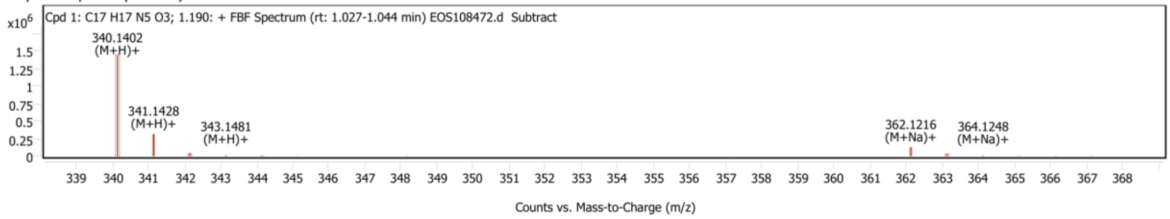

Spectrum Peaks

m/z	m/z (Calc)	Diff (ppm)	Abund	Height %	Height % (Calc)	Ion Species	Z
340.1402	340.1404	-0.73	1444334	100.00	100.00	(M+H)+	1
341.1428	341.1432	-1.30	317724	22.00	20.53	(M+H)+	1
342.1445	342.1457	-3.54	43488	3.01	2.62	(M+H)+	1
343.1481	343.1481	-0.16	5129	0.36	0.25	(M+H)+	1
344.1475	344.1505	-8.87	451	0.03	0.02	(M+H)+	1
362.1216	362.1224	-2.22	121209	100.00	100.00	(M+Na)+	1
363.1242	363.1252	-2.62	24665	20.35	20.52	(M+Na)+	1
364.1248	364.1277	-7.83	3612	2.98	2.62	(M+Na)+	1

Figure S55. *LC-HRMS data for compound* ***(3b)***

Compound Details

Cpd. 1: C13 H11 N5 O

Formula	RT	Mass	Mass (Tgt)	Abund	Algorithm
C13 H11 N5 O	1.036	253.0956	253.0964	1031472	FBF

Compound Spectra (overlaid)

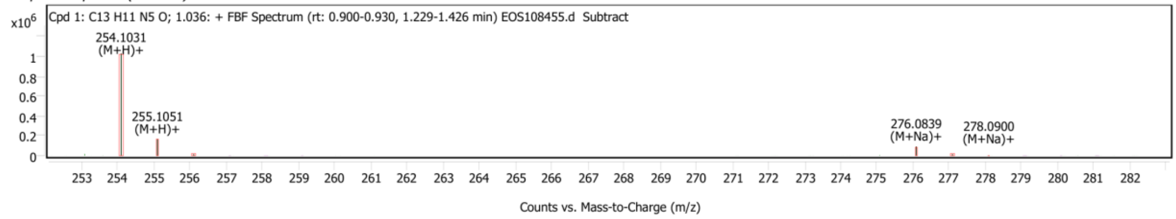

Spectrum Peaks

m/z	m/z (Calc)	Diff (ppm)	Abund	Height %	Height % (Calc)	Ion Species	Z
254.1031	254.1036	-2.12	1031472	100.00	100.00	(M+H)+	1
255.1051	255.1063	-4.89	164232	15.92	16.06	(M+H)+	1
256.1072	256.1088	-6.01	13966	1.35	1.42	(M+H)+	1
276.0839	276.0856	-5.96	92634	100.00	100.00	(M+Na)+	1
277.0871	277.0882	-4.18	15690	16.94	16.05	(M+Na)+	1
278.0900	278.0907	-2.59	1071	1.16	1.41	(M+Na)+	1

Figure S56. *LC-HRMS data for compound* ***(4a)***

Compound Details

Cpd. 1: C14 H13 N5 O

Formula	RT	Mass	Mass (Tgt)	Abund	Algorithm
C14 H13 N5 O	1.087	267.1113	267.1120	837035	FBF

Compound Spectra (overlaid)

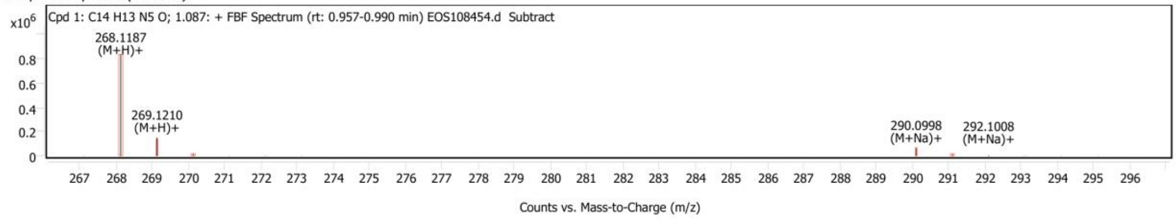

Spectrum Peaks

m/z	m/z (Calc)	Diff (ppm)	Abund	Height %	Height % (Calc)	Ion Species	Z
268.1187	268.1193	-2.20	837035	100.00	100.00	(M+H)+	1
269.1210	269.1220	-3.53	150196	17.94	17.17	(M+H)+	1
270.1221	270.1245	-9.08	14858	1.78	1.59	(M+H)+	1
290.0998	290.1012	-4.79	61493	100.00	100.00	(M+Na)+	1
291.1022	291.1039	-6.09	11161	18.15	17.16	(M+Na)+	1
292.1008	292.1065	-19.39	1763	2.87	1.59	(M+Na)+	1

Figure S57. *LC-HRMS data for compound* ***(4b)***

Compound Details

Cpd. 1: C14 H11 N5 O3

Formula	RT	Mass	Mass (Tgt)	Abund	Algorithm
C14 H11 N5 O3	0.746	297.0861	297.0862	82776	FBF

Compound Spectra (overlaid)

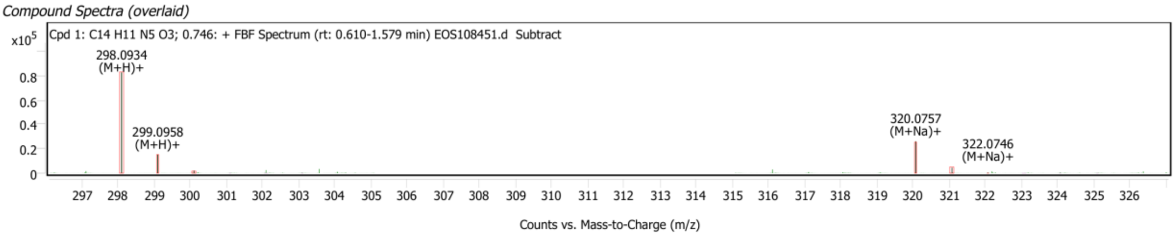

Spectrum Peaks

m/z	m/z (Calc)	Diff (ppm)	Abund	Height %	Height % (Calc)	Ion Species	Z
298.0934	298.0935	-0.34	82776	100.00	100.00	(M+H)+	1
299.0958	299.0962	-1.38	15287	18.47	17.22	(M+H)+	1
300.0975	300.0985	-3.36	2093	2.53	2.01	(M+H)+	1
320.0757	320.0754	0.94	26162	100.00	100.00	(M+Na)+	1
321.0776	321.0781	-1.52	4433	16.94	17.21	(M+Na)+	1
322.0746	322.0804	-18.23	467	1.79	2.01	(M+Na)+	1

Figure S58. *LC-HRMS data for compound* ***(5a)***

Compound Details

Cpd. 1: C15 H13 N5 O3

Formula	RT	Mass	Mass (Tgt)	Abund	Algorithm
C15 H13 N5 O3	0.841	311.0997	311.1018	988	FBF

Compound Spectra (overlaid)

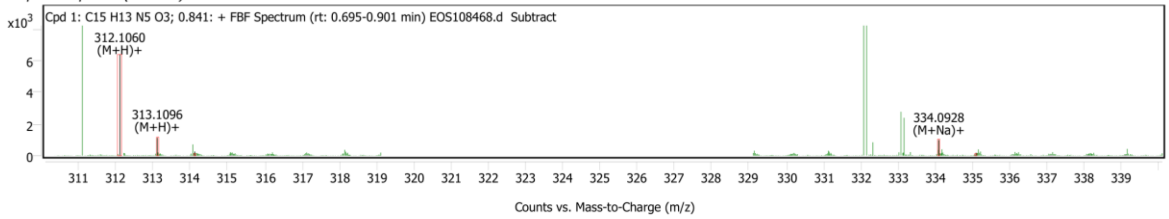

Spectrum Peaks

m/z	m/z (Calc)	Diff (ppm)	Abund	Height %	Height % (Calc)	Ion Species	Z
312.1060	312.1091	-10.11	6419	100.00	100.00	(M+H)+	1
313.1096	313.1119	-7.20	1130	17.61	18.33	(M+H)+	1
314.1204	314.1142	19.67	261	4.07	2.20	(M+H)+	1
334.0928	334.0911	5.20	988	100.00	100.00	(M+Na)+	1
335.0937	335.0938	-0.48	186	18.82	18.31	(M+Na)+	1

Figure S59. *LC-HRMS data for compound* ***(5b)***

Table S3. *Summary MS data for the synthesized compounds*

Compound	Structure	Calculated mass $[M+H]^+$	Found mass $[M+H]^+$	Calculated mass $[M+Na]^+$	Found mass $[M+Na]^+$
3a	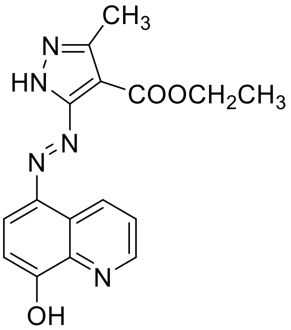	326.1248	326.1245	348.1067	348.1065
3b	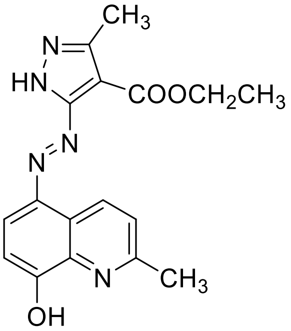	340.1404	340.1402	362.1224	362.1216
4a	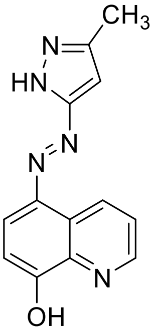	254.1036	254.1031	276.0856	276.0839

4b	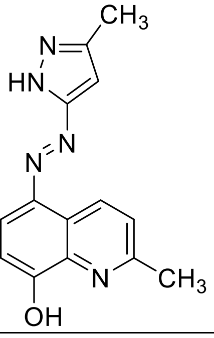	268.1193	268.1187	290.1012	290.0998
5a	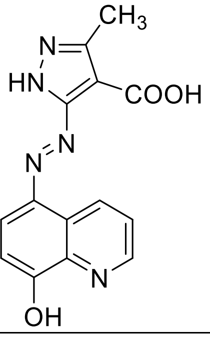	298.0935	298.0934	320.0754	320.0757
5b	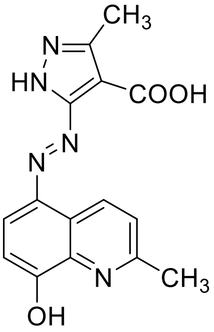	312.1091	312.1060	334.0911	334.0928

3. Biological activity evaluation data for compounds (3a), (3b), (4a), (4b), (5a), (5b)

Table S4. Antifungal, antibacterial and anticancer activities, assessed according to the OPENSCREEN protocols

**Overview**										
Compound	1. *Candida albicans* Conc./ % inhib/ Result	2. *Aspergillus fumigatus* Conc./ % inhib./ Result	3.*Candida auris* Conc./ % inhib./ Result	4. *Enterococcus faecalis* ATCC Conc./ % inhib./ Result	5. *Staphylococcus aureus* Conc./ % inhib./ Result	6. *Pseudomonas aeruginosa* Conc./ % inhib./ Result	7. *Escherichia coli* Conc./ % growth inhib/ Result	8. *Klebsiella pneumoniae* Conc./ % inhib./ Result	9. *Acinetobacter baumannii* Conc./ % inhib./ Result	10. Hep-G2 cells Conc./ % inhib./ Result/ IC_{50}
3a	50.0 µM/ 94.63/ **active**	50.0 µM/ 102.07/ **active**	50.0 µM/ 99.10/ **active**	50.0 µM/ 4.77/ inactive	50.0 µM/ 9.35/ inactive	50.0 µM/ -0.27/ inactive	50.0 µM/ -7.90 / inactive	50.0 µM/ -6.40 / inactive	50.0 µM/ -22.85 / inactive	10.0 µM/ 65.70/ **active**/ 7.649 µM
5b	50.0 µM/ 94.45/ **active**	50.0 µM/ 45.10/ inactive	50.0 µM/ 99.48/ **active**	50.0 µM/ 2.05/ inactive	50.0 µM/ 15.20/ inactive	50.0 µM/ -21.90/ inactive	50.0 µM/ -12.16 / inactive	50.0 µM/ 7.40 / inactive	50.0 µM/ -5.02 / inactive	10.0 µM/ 52.50/ **active**/ 7.324 µM
3b	50.0 µM/ 82.35/ **active**	50.0 µM/ 13.71/ inactive	50.0 µM/ 97.01/ **active**	50.0 µM/ 1.34/ inactive	50.0 µM/ 16.50/ inactive	50.0 µM/ -13.20/ inactive	50.0 µM/ -12.16 / inactive	50.0 µM/ -26.00 / inactive	50.0 µM/ -22.10 / inactive	10.0 µM/ 46.80/ **active**/ 7.614 µM
5a	50.0 µM/ 94.90/ **active**	50.0 µM/ 102.54/ **active**	50.0 µM/ 97.92/ **active**	50.0 µM/ 3.97/ inactive	50.0 µM/ 51.29/ inconclusive	50.0 µM/ 6.04/ inactive	50.0 µM/ -0.71/ inactive	50.0 µM/ 36.00/ inactive	50.0 µM/ -7.34/ inactive	10.0 µM/ 71.80/ **active**/ 2.708 µM
4a	50.0 µM/ 96.71/ **active**	50.0 µM/ 102.14/ **active**	50.0 µM/ 98.37/ **active**	50.0 µM/ -0.97/ inactive	50.0 µM/ 30.99/ inactive	50.0 µM/ 7.62/ inactive	50.0 µM/ -0.27/ inactive	50.0 µM/ 37.00/ inactive	50.0 µM/ -15.72/ inactive	10.0 µM/ 69.60/ **active**/ 2.989 µM
4b	50.0 µM/ 96.84/ **active**	50.0 µM/ 69.65/ inconclusive	50.0 µM/ 98.74/ **active**	50.0 µM/ 15.51/ inactive	50.0 µM/ 23.42/ inactive	50.0 µM/ 1.46/ inactive	50.0 µM/ -2.39/ inactive	50.0 µM/ -24.00/ inactive	50.0 µM/ -11.24/ inactive	10.0 µM/ 59.60/ **active**/ 7.238 µM

Table S5. Biological activity values, ordered for the individual compounds

**Compound 3a**	**% Inhib. (mean values)**	**Standard deviation [%]**
C. albicans	94.63	±5
C. auris	99.10	±5
A. fumigatus	102.07	±5
E. faecalis	4.77	±6
S. aureus	9.35	±6
P. aeruginosa	-0.27	±8
E.coli	-7.90	±8
K. pneumoniae	-6.40	±8
A. baumannii	-22.85	±8
Cell line permanent Hep-G2	65.70	±10
**Compound 3b**	**% Inhib. (mean values)**	**Standard deviation [%]**
C. albicans	82.35	±5
C. auris	97.01	±5
A. fumigatus	13.71	±5
E. faecalis	1.34	±6
S. aureus	16.50	±6
P. aeruginosa	-13.20	±8
E.coli	-12.16	±8
K. pneumoniae	-26.00	±8
A. baumannii	-22.10	±8
Cell line permanent Hep-G2	46.80	±10
**Compound 4a**	**% Inhib. (mean values)**	**Standard deviation [%]**
C. albicans	96.71	±5
C. auris	98.37	±5
A. fumigatus	102.14	±5
E. faecalis	-0.97	±6
S. aureus	30.99	±6
P. aeruginosa	7.62	±8
E.coli	-0.27	±8
K. pneumoniae	37.00	±8
A. baumannii	-15.72	±8
Cell line permanent Hep-G2	69.60	±10

**Compound 4b**	**% Inhib. (mean values)**	**Standard deviation [%]**
C. albicans	96.84	±5
C. auris	98.74	±5
A. fumigatus	69.65	±5
E. faecalis	15.51	±6
S. aureus	23.42	±6
P. aeruginosa	1.46	±8
E.coli	-2.39	±8
K. pneumoniae	-24.00	±8
A. baumannii	-11.24	±8
Cell line permanent Hep-G2	59.60	±10
**Compound 5a**	**% Inhib. (mean values)**	**Standard deviation [%]**
C. albicans	94.90	±5
C. auris	97.92	±5
A. fumigatus	102.54	±5
E. faecalis	3.97	±6
S. aureus	51.29	±6
P. aeruginosa	6.04	±8
E.coli	-0.71	±8
K. pneumoniae	36.00	±8
A. baumannii	-7.34	±8
Cell line permanent Hep-G2	71.80	±10
**Compound 5b**	**% Inhib. (mean values)**	**Standard deviation [%]**
C. albicans	94.45	±5
C. auris	99.48	±5
A. fumigatus	45.10	±5
E. faecalis	2.05	±6
S. aureus	15.20	±6
P. aeruginosa	-21.90	±8
E.coli	-12.16	±8
K. pneumoniae	7.40	±8
A. baumannii	-5.02	±8
Cell line permanent Hep-G2	52.50	±10
